# Photoinduced stepwise charge hopping in π-stacked perylene bisimide donor–bridge–acceptor arrays

**DOI:** 10.1038/s41557-025-01770-7

**Published:** 2025-03-14

**Authors:** Leander Ernst, Hongwei Song, Dongho Kim, Frank Würthner

**Affiliations:** 1 Universität Würzburg, Institut für Organische Chemie, Würzburg, Germany; 2 Spectroscopy Laboratory for Functional π-Electronic Systems and Department of Chemistry, Yonsei University, Seoul, Republic of Korea; 3 Universität Würzburg, Center for Nanosystems Chemistry, Würzburg, Germany

**Keywords:** Light harvesting, Supramolecular polymers

## Abstract

The mechanistic understanding of light-driven charge separation and charge-carrier transport within the frameworks of π-conjugated molecules is imperative to mimic natural photosynthesis and derive synthetic materials for solar energy conversion. In this regard, since the late 1980s, the distance and solvent dependence of stepwise (incoherent) charge-carrier hopping versus single-step (coherent) superexchange transport (tunnelling) have been studied in detail. Here we introduce structurally highly defined cofacially stacked donor–acceptor perylene bisimide arrays, which offer a high resemblance to natural systems. Similarity is achieved through controlling energy and electron transfer processes via intermolecular interactions between the π-stacked perylene bisimide subunits. Selective excitation of the donor induces electron transfer to the acceptor unit in polar solvents, facilitated by a ‘through-stack’ wire-like charge hopping mechanism with a low attenuation factor *β* = 0.21 Å^−1^, which suggests through-stack as being equally supportive for long-distance sequential electron transfer compared to the investigated ‘through-bond’ transfer along π-conjugated bridges.

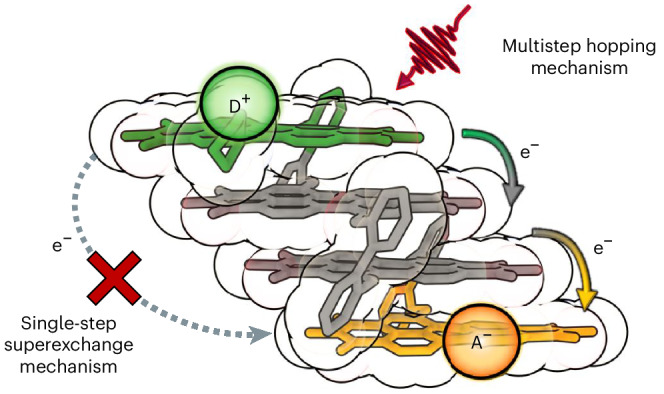

## Abstract (web summary)

Understanding the distance dependence and mechanistic behaviour of light-driven charge separation is crucial to advance artificial photosynthesis and solar energy conversion. It has now been shown that donor–bridge–acceptor null-coupled perylene bisimide arrays are good candidates for generating long-lived excitons and display ‘through-stack’ photoinduced electron transfer through pronounced π-orbital overlap.

## Main

Natural photosynthesis serves as a biological blueprint for the efficient conversion of solar excitation energy into electrochemical potential within the reaction centres of pigment–protein complexes^1,2^. The implementation of long-distance electron transfer (ET) minimizes any charge recombination processes, thereby generating long-lived charge-separated states alongside high quantum efficiencies^3,4^.

Artificial photosynthesis, aiming to replicate the natural photosynthetic apparatus, has been a long-standing scientific goal, which led to the development of artificial light-harvesting antennas and reaction centres^5–9^. Since the 1980s, a primary goal has been to clarify the role of distance and interconnecting matter in the (multistep) charge transfer process from the electron donor to the electron acceptor unit and the generation of long-lived charge-separated states^10^. Systematic investigations of distance-dependent photoinduced ET processes in D–B–A systems, where D is the electron donor, B is the bridge and A is the electron acceptor, with a variety of bridging units consisting of π-conjugated spacers^11–14^, DNA base pairs^15^ and peptides in proteins^16^, have provided crucial insights, suggesting that the ET from D to A may occur through coherent superexchange or incoherent charge hopping mechanisms.

Distinguishing between these mechanisms remains intricate and subject to ongoing debates dependent on molecular structure, electronic coupling and environmental conditions^15–20^. Multistep electron hopping enabling the transfer of charges over larger distances typically entails incoherent transfers between adjacent redox sites, influenced by the spatial arrangement of donor and acceptor units. This mechanism typically involves localized bridging states, resonant or lower in energy than the initial state, and exhibits a linear distance dependence. Regarding the wire-like ET between electron donor and acceptor, the charge is spread over the substantially populated intermediate bridge^20–23^. Conversely, superexchange single-step tunnelling involves a coherent electron exchange between donor and acceptor sites via mixing of virtual bridge states that must be energetically higher than the donor state. In addition, the charge is not spread over the entire bridge, and the intermediate bridging units are not substantially populated^20–23^. Understanding the relative contributions of these mechanisms and their dependence on structural and environmental factors is pivotal for tailoring the functionality of nanoscale biological counterparts, as well as the performance of functional materials in applications such as organic photovoltaics and optoelectronics^21,23^.

Perylene bisimide (PBI) dyes have emerged as promising contenders in D–A initiated charge separation and artificial photosynthesis^24–27^ due to their notable thermal, (electro)chemical and photophysical stability, alongside high molar extinction coefficients in the visible region^28^. While conventional molecular dimers have been extensively studied over the past decade, attention has been directed towards aggregates featuring no exciton coupling^29,30^. As such, null-coupled aggregates maintain their first excited singlet state at high energy, enabling unique photophysical properties such as singlet fission^31,32^, charge filtration^33,34^ and symmetry-breaking charge separation^35–37^, alongside the general suppression of undesired energy loss channels. Supramolecular engineering of null-coupled PBI π-stacks can be achieved via precise chromophore orientation, resulting in monomer-like absorption and emission features^31,38^. According to Spano’s extended exciton theory, the total exciton coupling in tightly stacked systems is determined by a combination of long-range Coulomb (dipole–dipole interaction) and short-range charge transfer-mediated (orbital overlap) couplings^39,40^. Whereas for most cofacially stacked π-systems one contribution dominates, for null-type aggregates destructively competing charge transfer-mediated and Coulomb coupling values are observed, leading to a negligible total coupling value. For PBIs, it has been demonstrated that the desired photophysical properties can be tailored by their π*–*π stacking geometries, as well as versatile core substitution^31,41^.

This study introduces D–B–A PBI (DA-PBI) arrays consisting of null-coupled PBI subunits as ideal systems for artificial photosynthesis by offering weak inter-PBI couplings, thereby reducing charge recombination processes and generating long-lived excitons, crucial for harnessing the generated charge carriers. With this class of arrays, we introduce beyond the widely studied ‘through-space’ and ‘through-bond’ pathways for photoinduced ET, a scarcely investigated ‘through-stack’ alternative. Here, we demonstrate that pronounced π-orbital overlap facilitates hole/electron transfer while avoiding exciton trap states, a prerequisite for active materials for light harvesting in solar cells. Our findings reveal that selective photoexcitation of the donor at 650 nm within **DA-PBI2** to **DA-PBI4** results in an ultrafast ET process. For **DA-PBI3** and **DA-PBI4**, we provide definitive experimental evidence that the ET to the acceptor unit in polar solvents occurs via a wire-like conduction along the π-stacked PBI units, with a small attenuation factor (*β*) of 0.2 Å^−1^. Notably, in non-polar solvents, the electron does not transfer to the final acceptor and remains trapped within the bridging units.

## Results and discussion

### Synthesis

**Fig. 1:**
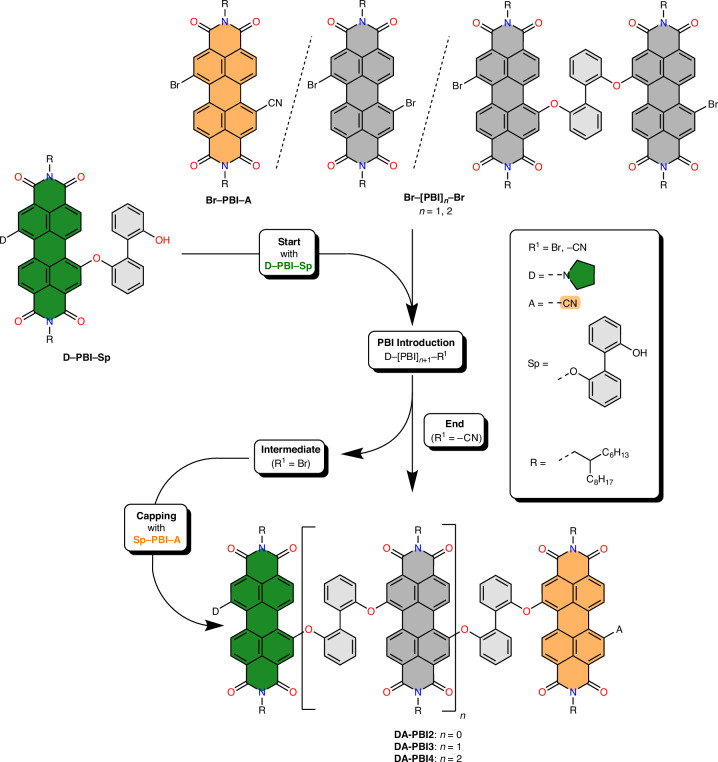
Synthetic procedure for the preparation of DA-PBI arrays. Starting with **D–PBI–Sp** (green), the centre units **Br–[PBI]**_***n***_**–Br** (grey) and end units **Br–PBI–A** or **Sp–PBI–A** (orange), the DA-dyad (**DA-PBI2**) up to the DA-tetrad (**DA-PBI4**) were obtained.

To specifically introduce charge-separated states in null-type PBI arrays, the outer PBI units were equipped with electron-donating pyrrolidine and electron-withdrawing cyano groups in the bay position of the perylene core, respectively, following the sequence shown in Fig. 1. The procedure relies on precise variation of monomeric excess without any necessity for protection groups. The synthetic sequence is initiated by a Williamson ether-like reaction of the starting unit (**D–PBI–Sp**, donor, green) with an excess of either **Br–PBI–A** to afford dyad **DA-PBI2** or one of the central PBI building blocks (**Br–[PBI]**_***n***_**–Br**, bridge, grey), followed by the acceptor end-group **Sp–PBI–A** (acceptor, orange) to give **DA-PBI3** and **DA-PBI4**, respectively (Sp = 2,2′-biphenol spacer).

### Optical and structural characterization

**Fig. 2:**
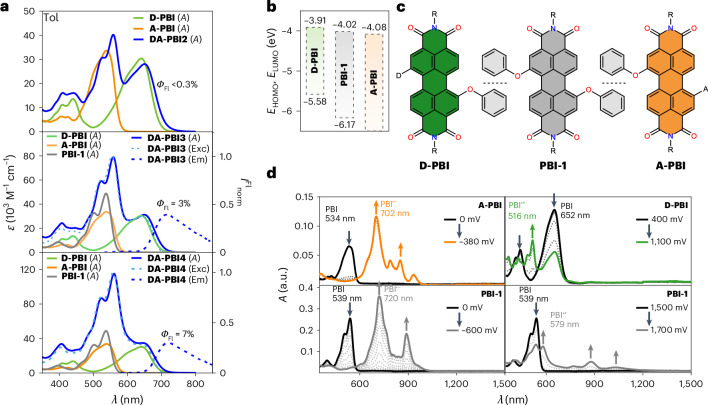
Optical and (spectro-)electrochemical characterization. **a**, The steady-state absorption (*A*) (blue, solid line), fluorescence (Em) (blue, dashed line) and excitation (Exc) spectra (light blue, dashed line) of **DA-PBI2** (top), **DA-PBI3** (middle), **DA-PBI4** (bottom) and their reference molecules **D-PBI** (green, solid line), **PBI-1** (grey, solid line) and **A-PBI** (orange, solid line) in toluene (Tol). *Φ*_Fl_ (Fluorescence quantum yield) and *I*^Fl^_norm_ (normalized fluorescence spectra). **b**, HOMO/LUMO energy levels of **D-PBI** (green), **PBI-1** (grey) and **A-PBI** (orange). All the potentials were calculated from voltammetry data referenced to the Fc^0/+^ redox couple (−5.15 eV versus vacuum level, potential/V versus Fc^0/+^) using the equations *E*_LUMO_ = −[*E*(M/M^−^) + 5.15 eV] and *E*_HOMO_ = −[*E*(M/M^+^) + 5.15 eV]. **c**, The molecular structures of the reference molecules **D-PBI** (green), **PBI-1** (grey) and **A-PBI** (orange). **d**, The spectroelectrochemical absorption spectra of the first reduction of **A-PBI** (orange) and oxidation of **D-PBI** (green) (top) and the first reduction and oxidation process of **PBI-1** (grey) (bottom) referenced to Ag/AgCl. The arrows indicate increasing or decreasing signal intensity. Source data

In the initial exploration of electronic coupling within the **DA-PBI2** to **DA-PBI4** assemblies, steady-state absorption and fluorescence measurements were conducted in solvents of varying polarity (Supplementary Fig. 13). These measurements were compared with those of corresponding reference compounds (**D-PBI**, **PBI-1** and **A-PBI**; Fig. 2c). Analogous to previously reported PBI arrays, interconnected through 2,2′-biphenol moieties^31,32^, a superposition-like behaviour with monomer-like absorption bands for the DA array substantiates the anticipated null-type excitonic coupling. The marginal bathochromic shift, observed in the absorption maxima of the DA-oligomers (Fig. 2a, blue solid lines) relative to the monomers, is explicable by polarization effects in the stacked conformation, as reported in prior studies^31,32^. In addition, a reduction in the extinction coefficient, observed for the absorption spectra of the DA arrays, can be attributed to the concomitant decrease in the absorption cross-section in the folded structural arrangement.

As an integral part of our molecular design strategy, the donor moieties within all DA-oligomers were meticulously engineered for selective excitation at longer wavelengths. Thus, while acceptor (**A-PBI**) and bridge (**PBI-1**) reference molecules exhibit closely aligned absorption regions in the PBI-typical spectral range (~540 nm, orange-to-red-coloured solutions), the intentionally chosen pyrrolidine-substituted PBI donor (**D-PBI**) exhibits strong absorption beyond 600 nm, leading to green-coloured solutions (Fig. 2a). Moreover, owing to pyrrolidine substitution in the bay position, these ‘green’ PBI chromophores are renowned for their charge transfer characteristics, rendering them particularly useful for photoinduced charge injection into the PBI arrays^28,42^. The substantially decreased emission of **DA-PBI2** (<0.3%), **DA-PBI3** (3%) and **DA-PBI4** (7%), relative to the **D-PBI** monomer (21%) in toluene, provides compelling evidence for the anticipated charge separation process. Moreover, the emission of all species is nearly entirely quenched in solvents of higher polarity. Furthermore, the excitation spectra of all dyads (Fig. 2a) closely match the absorption spectra, thereby indicating a highly efficient energy transfer (EnT) process to the lowest energy chromophore (**D-PBI**). Consequently, the emission spectra of all DA arrays closely resemble that of the **D-PBI** chromophore.

To further elucidate the proper alignment of molecular orbitals conducive to charge separation, cyclic voltammetry and differential pulse voltammetry were conducted on the reference molecules in dichloromethane (DCM) (Supplementary Fig. 14). The highest occupied molecular orbital (HOMO)/lowest unoccupied molecular orbital (LUMO) levels (Fig. 2b), derived from the redox data, reveal an energy cascade favouring charge separation upon excitation of the donor moiety (−3.91 eV), followed by a subsequent electron hopping transport via the **PBI-1** bridge to the acceptor (−4.08 eV). To corroborate any charge transfer via the PBI spectroelectrochemistry (SEC) has been performed for the reference molecules in dry DCM under argon (Fig. 2d and Supplementary Figs. 15–17). Upon increasing negative or positive potentials, characteristic signals of radical anions (**A-PBI**, 702 nm; **PBI-1**, 720 nm) and radical cations (**PBI-1**, 579 nm; **D-PBI**, 516 nm) could be recorded. Their stability and distinctive spectral features over extended time periods provide a robust situation for time-resolved spectroscopy studies.

Considering the limitations of ultraviolet–visible light absorption spectra in providing comprehensive structural insights, especially for null-coupled dye arrays, the packing arrangement of the PBI units in the arrays was subjected to thorough ^1^H nuclear magnetic resonance investigations by two-dimensional rotating frame nuclear Overhauser effect spectroscopy and homonuclear correlation spectroscopy in deuterated 1,1,2,2-tetrachloroethane (TCE-*d*_2_) at 384 K (Supplementary Figs. 1–3 and 31–58). The vicinity of the chromophores, even at elevated temperature, is evidenced by intense cross-signals between perylene core protons, indicating the tightly stacked geometry. These can be unequivocally identified as true couplings through space, exclusively arising from the intramolecular proximity of the PBI chromophores. Furthermore, the perylene core protons of the DA arrays exhibited pronounced up-field shifts compared with the reference monomers, owing to increasing shielding effects resulting upon π-stacking. Finally, diffusion ordered spectroscopy has been conducted, revealing a progressive increase in hydrodynamic radii from **DA-PBI2** (11.7 Å) to **DA-PBI4** (15.1 Å) consistent with previously reported values for stacked null-type aggregates and the radii obtained from density functional theory (DFT) calculations^31^ (Supplementary Figs. 4–6).

### Quantum chemical simulation and Weller calculations

**Fig. 3:**
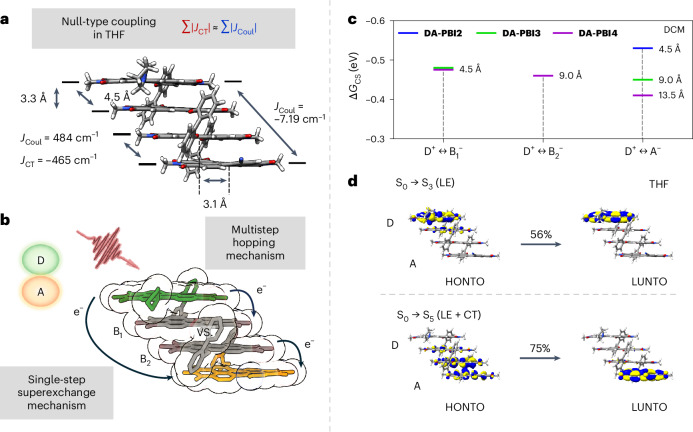
Quantum chemical and Gibbs free energy calculations. **a**, The molecular structure of **DA-PBI4** with long-range Coulomb and short-range charge transfer coupling energies calculated in THF. **b**, The illustration of possible pathways for the photoinitiated ET via multistep hopping (right) and a single-step superexchange (left) mechanism for **DA-PBI4** from the donor (D, green), over the bridging units (B_1_ and B_2_, grey) to the acceptor (A, orange). **c**, A plot of the Gibbs free energy values for the charge separation processes in DCM according to Weller calculations. **d**, The highest occupied (HONTO) (left) and lowest unoccupied (LUNTO) (right) NTOs (isovalue of 0.02) for the main contribution of the local exciton (LE) S_0_–S_3_ (top) and charge transfer (CT) S_0_–S_5_ (bottom) transitions of the **DA-PBI4** array in THF. Source data

To substantiate the optical null-type features and the tightly stacked geometry, further in-depth structural insights were derived from geometry optimized structures at the level of long-range-corrected DFT calculations (wB97X-D/def2-SVP) (Fig. 3 and Supplementary Table 1). The tightly stacked configurations obtained for the DA series and exemplary discussed for **DA-PBI4**, reveal a longitudinal slip of 3.1 Å between the π-stacked adjacent PBI moieties, characterized by van der Waals distances of 3.3 Å (Fig. 3a). In combination with a negligible transverse shift, a substantial charge transfer coupling is anticipated due to a pronounced HOMO/LUMO orbital overlap. These results are comparable with already known and structurally related null-type PBI aggregates without peripheral donor and acceptor functionalization^31,32^.

Upon performing time-dependent DFT (TD-DFT) calculations, an intermediate ET integral (*t*_e_ = 677 cm^−1^) was identified for the given spacer-controlled structure in tetrahydrofuran (THF). According to the classical Marcus theory for coupled chromophores, ET rates are strongly dependent on high electron (or hole) transfer integrals, free Gibbs and low reorganization energies^23,43^. Larger longitudinal displacements of around 4.5 Å promise even higher ET integrals with presumably also lower *β* values^23,44^ (Supplementary Fig. 9). For **DA-PBI4**, the calculations facilitated the determination of long-range Coulomb (*J*_Coul_ = 484 cm^−1^) and short-range charge transfer (*J*_CT_ = −465 cm^−1^) coupling values between neighbouring PBI units in THF (Fig. 3a). Furthermore, the total Coulomb coupling is complemented by weaker couplings between PBI dyes at distances of three (−91.9 cm^−1^) and four (−7.19 cm^−1^) units (Supplementary Tables 2 and 3). These quantum chemical simulations support the destructively competing Coulomb (positive) and charge transfer (negative) coupling values on the total coupling between adjacent dyes. The negligible effect of the total coupling on the optical properties is in good accordance with the monomer-like steady-state absorption spectra (Fig. 2a) and similar symmetric null-type π-stacked systems reported in literature^31,32,45^.

For a first rough evaluation of the respective ET pathways upon photoexcitation of the **DA-PBI4** array, the free Gibbs energies for the charge separation (Δ*G*_CS_) and charge recombination (Δ*G*_CR_) were calculated applying a supplemented Weller equation^46^ (equations (1) and (2))

1 $$\begin{array}{l}\Delta {G}_{{\rm{CS}}}={\mathrm{e}}\left[{E}_{{\rm{Ox}}({\rm{D}})}-{E}_{{\rm{Red}}({\rm{A}})}\right]-{E}_{00}-\frac{1}{4\pi {\varepsilon }_{0}{\varepsilon }_{{\rm{B}}}}\mathop{\sum }\limits_{{{i}}}\mathop{\sum }\limits_{{{j}}}\frac{{q}_{{{i}}}^{\left({{\rm{a}}}\right)}\times {q}_{{{j}}}^{\left({{\rm{b}}}\right)}}{\left|{r}_{{{i}}}^{\left({{\rm{a}}}\right)}-{r}_{{{j}}}^{\left({{\rm{b}}}\right)}\right|}\\\qquad\qquad-\frac{{\mathrm{e}}^{2}}{8\pi {\varepsilon }_{0}}\left(\frac{1}{{r}_{{\rm{D}}}}+\frac{1}{{r}_{{\rm{A}}}}\right)\left(\frac{1}{{\varepsilon }_{{\rm{CV}}}}-\frac{1}{{\varepsilon }_{{\rm{S}}}}\right)\end{array}$$

2 $$\Delta {G}_{\rm{CR}}=-\left(\Delta {G}_{\rm{CS}}+{E}_{00}\right)$$

Here, *E*_Ox(D)_ and *E*_Red(A)_ denote the first oxidation and reduction potentials of **D-PBI** and **A-PBI**, respectively. *E*_00_ represents the excited-state energy, derived from the average value of absorption and emission maxima of the donor. The point charge on atom *i* of chromophore a is defined by *q*_*i*_^(a)^, and the position vector of the respective point charge is given by *r*_*i*_^(a)^ (refs. ^47–50^). The coordinates and Mulliken point charges of each atom are determined from a DFT geometry optimization of the analysed oligomer in DCM, followed by single point calculations of charged monolayers (A^−^, B^−^, D^+^) extracted from the geometry optimized structures (wB97X-D/def2-SVP). In addition, *r*_D_ and *r*_A_ correspond to the effective ionic radii of the donor and acceptor, respectively. *ε*_B_, *ε*_S_ and *ε*_CV_ denote the dielectric constants of the shielding solvent, spectroscopic solvent and the solvent employed for electrochemical measurements, respectively. Since the redox potentials were acquired in DCM, the final term (Born ionic solvation energy) in equation (1) can be neglected for this solvent. The dimer system **DA-PBI2** (Δ*G*_CS_ = −0.53 eV) exclusively provides a direct ET pathway from the donor moiety to the acceptor unit. The longer systems **DA-PBI3** (Δ*G*_CS_ = −0.45 eV, D^+^ → A^−^) and **DA-PBI4** (Δ*G*_CS_ = −0.41 eV, D^+^ → A^−^), however, allow both, a direct, through-stack mediated band-like and a hopping pathway from the acceptor to the donor (Fig. 3b). The free energy values are summarized in Fig. 3c and Supplementary Table 8. The Gibbs free energies for the through-stack charge separation and charge recombination processes across all DA-oligomers were determined to be exothermic in DCM, thereby confirming the thermodynamic feasibility of charge separation via both pathways^51,52^.

To gain deeper insights into the behaviour of excited states following initial vertical excitation and to support the findings from the Weller analysis, natural transition orbital (NTO) plots were generated^53,54^. As shown in Fig. 3d, for **DA-PBI4** in THF (third lowest excited state, S_3_), the main contributing NTO pair (56%) represents a partially delocalized electronic state primarily localized at the donor, which is selectively excitable within the donor absorption region. The fifth lowest calculated vertical excited state (Fig. 3d, bottom) also exhibits a partially delocalized electronic state but is primarily localized at the bridge/acceptor, displaying pronounced charge transfer character. Upon excitation of the donor PBI at 650 nm and subsequent solvent reorientation, charge-separated states (D^+^, A^−^) are expected to be accessed over time, either through incoherent stepwise charge hopping or a direct coherent process (Fig. 3b).

### Transient absorption measurements

Femtosecond transient absorption spectroscopy (fs-TA) has been applied to investigate the excited-state dynamics in the molecular systems **DA-PBI2** to **DA-PBI4** and their respective monomers (**D-PBI** and **A-PBI**; Supplementary Figs. 18–21) in Tol and THF. The thermodynamic analysis indicates that the transition from D^*^–(B)_*n*_–A (*n* = 0, 1, 2) via (D^+^–(B)_*n*_^−^–A) to (D^+^–(B)_*n*_–A^−^) is an exergonic and thermodynamically allowed process (Fig. 3c). To experimentally validate these calculations, fs-TA spectra of **DA-PBI2** to **DA-PBI4** in Tol and THF were acquired using a 650 nm pump pulse to selectively excite the donor absorption band. The transient absorption spectra in Supplementary Fig. 22 and global fitting analysis in Supplementary Fig. 23 illustrate the ET from the donor moiety to the acceptor unit for **DA-PBI2**, occurring on ultrafast timescales of 2.0 and 0.9 ps in Tol and THF, respectively. This results in the formation of the (D^+^–A^−^) state, as indicated by the emergence of the PBI acceptor bleach at around 550 nm and the radical anion absorption at 702 nm. The charge recombination process from D^+^–A^−^ back to the ground state exhibits time constants of 143 and 7.7 ps in Tol and THF, respectively, with no discernible long-lived signals.

**Fig. 4:**
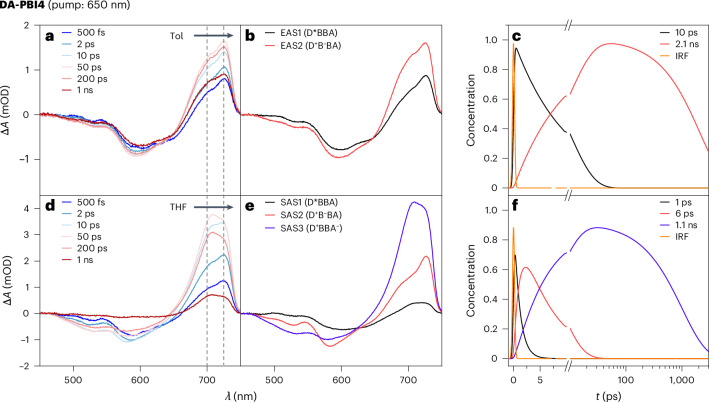
Transient absorption spectroscopy with 650 nm pump. **a**–**c**, Transient absorption spectra (**a**), evolution-associated spectra (**b**) and the associated population evolution curves (**c**) of different components for **DA-PBI4** in Tol. **d**–**f**, Transient absorption spectra (**d**), species-associated spectra (**e**) and the associated population evolution curves (**f**) of different components for **DA-PBI4** in THF. The associated spectra are obtained from a global fitting analysis with a 650 nm excitation. The dotted lines (grey) indicate the main radical anion signals of **PBI-1** (725 nm) and **A-PBI** (702 nm). mOD, milli-optical density; IRF, instrument response function. Source data

Transient absorption measurements for **DA-PBI3** and **DA-PBI4** were conducted under identical conditions (Fig. 4 and Supplementary Fig. 24). In Tol, alongside the ground state bleach (GSB) contributions, the transient absorption spectra for **DA-PBI3** (Supplementary Fig. 24a) and **DA-PBI4** (Fig. 4a) reveal the emergence of an excited state absorption (ESA) band at 725 nm, corresponding to the **PBI-1** bridge radical anion absorption band (D^+^–(B)_*n*_^−^–A), corroborated by SEC spectra (Fig. 2d). Global fitting analysis, applying a sequential model (Fig. 4b,c and Supplementary Fig. 25a,b), yields a charge separation time constant (*τ*_CS1_) from the donor to the bridge of 7 ps and a charge recombination lifetime (*τ*_CR1_) of 900 ps for **DA-PBI3**, as well as *τ*_CS1_ = 10 ps and *τ*_CR1_ = 2,100 ps for **DA-PBI4** (Extended Data Table 1). In THF, the transient absorption spectra for **DA-PBI3** (Supplementary Fig. 24c) and **DA-PBI4** (Fig. 4d) exhibit more intricate evolution information. Within the first 2 ps, the transient absorption spectra resemble those in Tol, featuring a GSB from 500 to 650 nm and an ESA band from 650 to 750 nm with a maximum at 725 nm. However, after 2 ps, the ESA signal shifts from 725 to 702 nm, corresponding to the PBI acceptor radical anion absorption band (D^+^–(B)_*n*_–A^−^), corroborated by SEC spectra (Fig. 2d). Herein, a corresponding target model for global fitting is proposed to reproduce fs-TA data, whose resulting species-associated spectra and corresponding time-dependent concentration kinetics are provided in Fig. 4e,f (Supplementary Fig. 25c,d), and the corresponding time constants are listed in Extended Data Table 1. For **DA-PBI3** and **DA-PBI4**, the time constants of the population of the (D*–(B)_*n*_–A), (D^+^–(B)_*n*_^−^–A) and (D^+^–(B)_*n*_–A^−^) states were determined by global analysis, which yields 1.2, 2.4 and 80 ps and 1, 6 and 1,100 ps, respectively. These time constants evidence the ultrafast extraction of the electron from the initially populated radical anion state on **D-PBI** into the neighbouring PBI bridge units and its slower transfer to the final **A-PBI**.

**Fig. 5:**
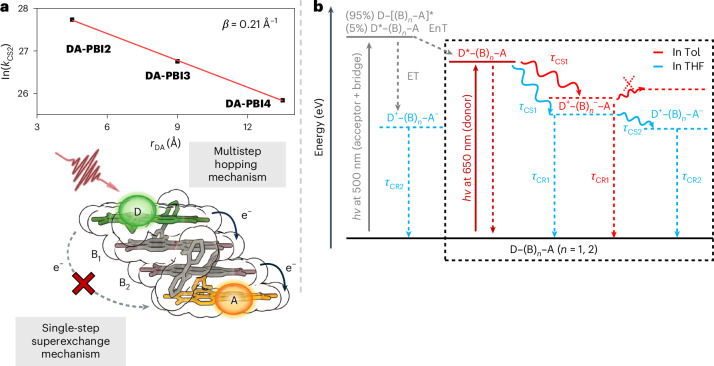
Attenuation factor and deactivation pathway illustration. **a**, Top: a plot of ln(*k*_CS2_) versus *r*_DA_ for **DA-PBI2**, **DA-PBI3** and **DA-PBI4** in THF. The red line is the linear fit to the data. Bottom: an illustration of the observed photoinitiated ET via stepwise hopping (right) instead of single-step superexchange (left) mechanism for **DA-PBI4** in THF from the donor (D, green), over the bridging units (B_1_ and B_2_, grey) to the acceptor (A, orange). **b**, The energy levels and deactivation pathways for the electronic states relevant to the energy-coupled ET pathways for **DA-PBI3** and **DA-PBI4** in Tol and THF upon excitation at 650 nm (inside the box) and 500 nm (outside the box).

Figure 5a presents a logarithmic plot illustrating *τ*_CS2_ in THF as a function of the donor–acceptor distance (*r*_DA_). The exponential dependence of the rate constant for the ET to the PBI acceptor with respect to *r*_DA_ is evident, with a phenomenological attenuation factor of *β* = 0.21 Å^−1^. The value of *β* is often used to distinguish between the different charge migration mechanisms that have been proposed for donor–bridge–acceptor systems. A large value of *β* (~1 Å^−1^) represents a strong dependence of the ET rate on the donor–acceptor distance and is characteristic for a superexchange single-step tunnelling process, which has been found for ET across ‘isolating matter’ in proteins and rigid alkane σ-bridges^55,56^. In addition, for DNA bridges, which are structurally more related to our π-stacks, the *β* values in the same range are most common, albeit some examples for a change of mechanism from tunnelling to hopping, enabling transfer of charges over longer distances, has been observed^56–60^. Owing to the presence of an inhomogeneous ‘conduction path’ (GC versus AT base pairs) and the special aqueous environment (highly polar, hydrogen bonding and counterions), this most-investigated class of π-stacked bridges are, however, not easily comparable with our PBI stacks. Apart from DNA, another example based on cofacially π-stacked bridges, that is, benzene stacks positioned between a porphyrin donor and a quinone acceptor, has been reported by Therien and coworkers. Here, a substantially smaller value of *β* = 0.43 Å^−1^ was observed for charge-carrier separation that was rationalized to originate from arene–arene compressions, thereby improving the tunnelling process^61^.

Otherwise, for smaller attenuation factors, in particular if measured over larger distances, there is evidence for a mechanistic change towards the stepwise incoherent hopping mechanism in which the transfer rate and attenuation factors are strongly governed by the electronic coupling among the building blocks utilized for the bridge, which can provide faster band-like or slower hopping transfers, similar as known for organic semiconductors^62,63^. In this regard, we see great similarities between our results for cofacially stacked PBI bridges and the results obtained by Wasielewski and coworkers^22^ for π-conjugated oligomeric 2,7-fluorenone bridges. For the latter, a value of *β* = 0.34 Å^−1^ was observed for the distance dependence of photoinduced ET from a anthracenyl-julolidine donor to a naphthalene bisimide acceptor. The fact that the through-stack charge transport across the cofacially stacked PBI π-bridge is less attenuated corroborates the more wire-like character, that is, a stronger electronic coupling between the PBI units compared with the through-bond π-conjugated fluorenone, thereby supporting ET. The same holds true if compared with another π-conjugated bridge, that is, oligomeric *para*-phenylene, for which attenuation factors between 0.32 and 0.67 Å^−1^ were reported^12,64^. We attribute the excellent wire-like conduction across our PBI stacks to the substantial ET integrals (Supplementary Table 3), which originate from a high LUMO–LUMO overlap for the arrangement of the PBIs that is indeed quite similar to packing arrangements observed for the best PBI based n-channel organic semiconductor materials with charge-carrier mobilities >1 cm^2^ V^−1^ s^−1^ (refs. ^65^). Moreover, as depicted in Supplementary Fig. 9, the ET integrals are highly sensitive to orientational changes and should be maximized upon increasing the longitudinal shift by varying spacer moieties in future projects. This should result in enhanced ET rates and even lower *β* values, probably outcompeting linear π-conjugated bridges.

The phenomenological *β* value observed for the incoherent stepwise ET process in our system indeed suggests a biased charge hopping mechanism, where the electrostatic interactions (D^+^ and B^−^) between charges introduce asymmetries in the forward and backward electron hopping rates. Notably, in the case of **DA-PBI3** and **DA-PBI4** in Tol, the electron is unable to reach the terminal PBI acceptor unit, resulting in the final excited state with the electron localized within the bridge. In this scenario, the electrostatic attraction between D^+^ and B^−^ leads to a higher rate of electron back hopping towards the positively charged donor compared with further hopping towards the neutral acceptor. In contrast, as illustrated in Fig. 5b, the behaviour in THF indicates that the energy level of the final charge-separated state (D^+^–(B)_*n*_–A^−^) is more sensitive to a polar environment than that of the intermediate charge-separated state (D^+^–(B)_*n*_^−^–A). Consequently, the observed stabilization of the final charge-separated state induces a substantial driving force towards a more band-like ET from the donor moiety to the acceptor unit in THF.

**Fig. 6:**
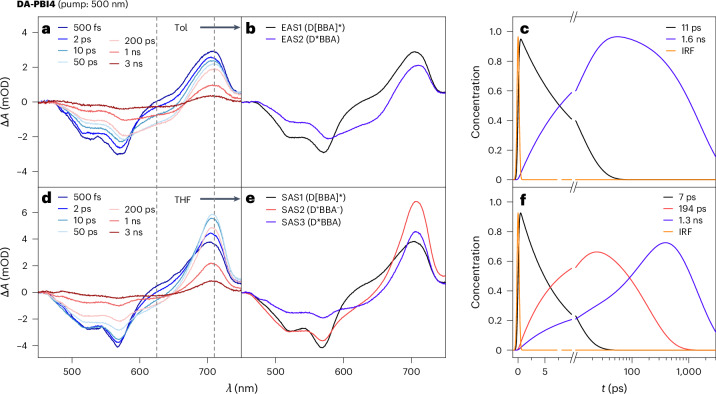
Transient absorption spectroscopy with 500 nm pump. **a**–**c**, Transient absorption spectra (**a**), evolution-associated spectra (**b**) and the associated population evolution curves (**c**) of different components for **DA-PBI4** in Tol. **d**–**f**, Transient absorption spectra (**d**), species-associated spectra (**e**) and the associated population evolution curves (**f**) of different components for **DA-PBI4** in THF. The associated spectra obtained are from a global fitting analysis with a 500 nm excitation. The dotted lines (grey) indicate the main radical anion signal of **PBI-1** (710 nm) and the GSB of **DA-PBI4** (625 nm). Source data

The red-shifted absorption of **D-PBI** introduces a spectral overlap between the emission of **A-PBI**/**PBI-1** and the absorption of **D-PBI** (Fig. 2). Consequently, an EnT decay pathway is anticipated for both **DA-PBI3** and **DA-PBI4** upon excitation of acceptor and bridge units. The fs-TA spectra for **DA-PBI2** to **DA-PBI4** in Tol and THF were recorded using a 500-nm pump pulse, selectively exciting the acceptor–bridge absorption band to a predominant extent (95%). As depicted in Supplementary Fig. 26, the fs-TA spectra of **DA-PBI2** in both Tol and THF, excited at 500 nm, exhibit almost identical behaviour to that observed at 650 nm. For **DA-PBI3**, beyond the formation of the ESA band at approximately 710 nm, the initial picoseconds of the fs-TA spectra (Supplementary Fig. 28) reveal a rapid generation of the GSB band centred at 625 nm in both solvents. This phenomenon is linked to the donor absorption, indicating an ultrafast and effective EnT process. The fs-TA spectra of **DA-PBI4** (Fig. 6d) in THF closely resemble those of **DA-PBI3**. In addition, Fig. 6a reveals that in Tol, there is no ESA band formation or ET process for **DA-PBI4**, only an EnT process is occurring. A global fitting analysis with a target model yields a solvent polarity-independent EnT time constant of 10 ps (Extended Data Table 2).

As depicted in Fig. 5b, upon excitation of **DA-PBI3** and **DA-PBI4** at 500 nm (corresponding to ~95% PBI acceptor–bridge and 5% PBI donor excitation), an energy-coupled ET process ensues, involving the transmission of energy from the acceptor–bridge units to the PBI donor. The corresponding TA kinetic traces (Supplementary Fig. 30) emphasize that both EnT and ET processes (occurring at 1, 3 and 9 ps for **DA-PBI2** to **DA-PBI4** in THF) exhibit a decelerated rate with an increasing length of the DA-PBI arrays. Simultaneously, charge recombination processes (at 9, 87 and 1,300 ps for **DA-PBI2** to **DA-PBI4** in THF) are also decelerated, resulting in an increasing charge separation lifetime. Furthermore, an examination of the ET (7.4 and 3.3 ps in Tol and THF) and charge recombination processes (941 and 87.5 ps in Tol and THF) for **DA-PBI3** reveals a dependence on solvent polarity. Conversely, the EnT process appears to be solvent polarity independent, as evidenced by time constants of 1/1 and 11/7 ps for **DA-PBI3** and **DA-PBI4** in Tol/THF (Extended Data Table 2).

## Conclusion

In summary, we reported a comprehensive exploration of ET dynamics for slip-stacked donor–acceptor-functionalized PBI arrays of up to four PBI subunits, providing a highly defined geometry with counteracting long-range Coulomb and short-range charge transfer interactions (‘null-coupled’ PBI arrays). Our meticulous analysis revealed an efficient and fast photoinduced ET from donor to acceptor units via cofacially stacked PBI units. Upon excitation of the donor moiety at 650 nm in a polar solvent (THF), a wire-like ET is observed with a smaller attenuation factor compared with commonly used π-conjugated bridges based on *para*-oligophenylene. Remarkably, this wire-like mechanism is found to be suppressed in a non-polar solvent (Tol) owing to the lack of driving force. Accordingly, we envision that with stronger PBI acceptor units, we may accomplish ET processes across far more extended PBI arrays based on the small attenuation factor. Our detailed studies of structurally well-defined π-stacked DA-PBI arrays constitutes an important fundamental step towards the understanding of competitive photophysical processes in structurally tailored nanosystems that serve as synthetic counterparts to natural photosynthetic complexes and models systems for the design of a variety of photofunctional materials.

## Methods

### Synthesis

To ensure a high solubility of all PBI arrays even in low-polarity solvents, branched 2-hexyldecyl alkyl chains were introduced as solubilizing imide substituents. The detailed synthetic procedures and characterization of all molecules are provided in the Supplementary Information (Supplementary Information, Schemes 1 and 2).

### Cyclic voltammetry

Cyclic voltammetry was performed in dry DCM under argon, yielding their redox potentials referenced to the Fc^0/+^ redox couple (potential/V versus Fc^0/+^).

### Quantum chemical calculations

To scrutinize the electronic coupling between PBI chromophores in their slip-stacked arrangement, TD-DFT (wB97X-D/def2-SVP) calculations were conducted, applying a polarizable continuum solvation model in THF and Tol. The effective electron and hole transfer integrals were derived applying the Amsterdam Density Functional (PW91/TZP) program. The effective electron and hole transfer integrals for functionalized, non-planar PBI chromophores of **DA-PBI2** were calculated and plotted as a function of the longitudinal shift from 0 to 11.4 Å at constant π-distances of 3.3 Å (Supplementary Fig. 9). It must be noted that structural fluctuations substantially influence coupling values, especially those derived from orbital overlap^45^. As the couplings are simply estimated to account the optically observed null-coupling behaviour, any fluctuation dependence has been neglected.

NTOs were generated using the MultiWFN 3.8 software package^53^. These NTO plots, specifically the highest occupied and lowest unoccupied orbitals (Fig. 3d and Supplementary Figs. 10–12), were obtained from TD-DFT calculations to qualitatively interpret the excited-state character. Due to the involvement of numerous molecular orbital transitions, the excited-state interpretation based solely on molecular orbital pairs is insufficient.

### Weller calculations

The Weller equation traditionally assumes point charges located on hard spheres, separated by the centre-to-centre distance between the donor and acceptor. However, for systems with large π-surfaces, the charges are delocalized over the donor and acceptor fragments, necessitating the use of distributed point charges^47^. Furthermore, the uniform solvation of neighbouring chromophores complicates the modelling of ionic interactions within the local electrostatic environment, making it challenging to apply a simple dielectric continuum model^48–50^. Despite these complexities, the dielectric constant of dichloromethane (*ε* = 8.93) was used for the shielding factor of the surrounding solvent and bridging units within the π-stack (*ε*_B_), to avoid introducing additional uncertainties.

## Online content

Any methods, additional references, Nature Portfolio reporting summaries, source data, extended data, supplementary information, acknowledgements, peer review information; details of author contributions and competing interests; and statements of data and code availability are available at 10.1038/s41557-025-01770-7.

## Supplementary information

Supplementary Information Supplementary Figs. 1–72, Schemes 1 and 2, Tables 1–14, text and discussion.

Supplementary Data 1 Source data for Supplementary Figs. 13, 15–17 and 18–30.

## Source data

Source Data Fig. 2 Source data for Fig. 2.

Source Data Fig. 3 Source data for Fig. 3.

Source Data Fig. 4 Source data for Fig. 4.

Source Data Fig. 6 Source data for Fig. 6.

## Figures and Tables

**Extended Data Table 1:**
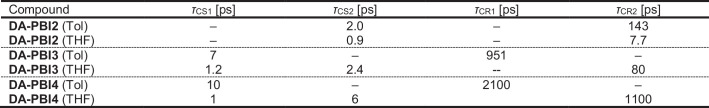
Transient absorption spectroscopy with 650 nm pump

Electron transfer and charge recombination time constants upon excitation at 650 nm.

*τ*_CS1_ and *τ*_CR1_ are related to the electron transfer and recombination processes from the donor to the bridge. *τ*_CS2_ and *τ*_CR2_ are related to the electron transfer and recombination processes from the donor to the acceptor.

**Extended Data Table 2:**
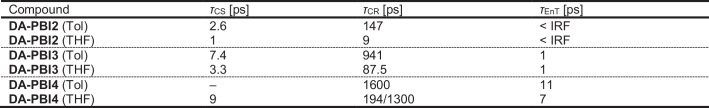
Transient absorption spectroscopy with 500 nm pump

Time constants for electron transfer, charge recombination and energy transfer upon 500 nm excitation.

## Data Availability

All experimental procedures, analytical data and computational data supporting the findings of this study are available within the Article and its Supplementary Information. Source data are provided with this paper and have been made available via Zenodo at 10.5281/zenodo.14526211 (ref. ^66^).
