# Supplementary material for: Photoinduced stepwise charge hopping in π-stacked perylene bisimide donor–bridge–acceptor arrays
Source: Nat Chem. 2025 Mar 14;17(5):767–76. doi: 10.1038/s41557-025-01770-7 (PMC12055566; doi:10.1038/s41557-025-01770-7)
Supplement: Supplementary file 1 — Supplementary Figs. 1–72, Schemes 1 and 2, Tables 1–14, text and discussion. [file 41557_2025_1770_MOESM1_ESM.pdf]

nature chemistry

Supplementary information https://doi.org/10.1038/s41557-025-01770-7

Photoinduced stepwise charge hopping in π-stacked perylene bisimide donor–bridge–acceptor arrays

In the format provided by the authors and unedited

Table of the contents

1. Materials and Methods 2

2. Synthesis 6

3. 2D NMR Spectroscopy 23

4. Quantum Chemical and Weller Calculations 28

5. UV/Vis Absorption and Fluorescence Spectroscopy 38

6. Cyclic Voltammetry 42

7. Spectroelectrochemistry 43

8. Time-Resolved Spectroscopy 45

9. NMR Spectroscopy 53

10. Mass Spectrometry 81

11. References 88

1. Materials and Methods

Chemicals

All commercial chemicals and reagents, unless otherwise stated, were used without further purification. All air or moisture sensitive reactions were carried out under nitrogen atmosphere by standard Schlenk techniques. The solubilizing 2-hexyldecylamine (**A-1**)[1] and 1,7-dibromoperylene-3,4:9,10-bis(dicarboximide) (**1**)[2,3] were synthesized according to literature known procedures.

Purification

Column chromatography was performed on silica-gel (particle size 0.040-0.063 mm) with freshly distilled solvents as eluents.

Thin layer chromatography (TLC) was performed on pre-coated TLC-sheets (ALUGRAM Xtra SIL G/UV254, MACHERY-NAGEL).

Recycling gel permeation chromatography (GPC) was performed on a Shimadzu Prominence CBM (LC-20AD Prominence Pump; SPDMA20A Prominence Diode Array Detector) with three preparative columns as stationary phase (Japan Analytical Industries Co., Ltd.; JAIGEL-1H, JAIGEL-2H and JAIGEL-2.5H) and chloroform (HPLC grade, stabilized with 0.1 % ethanol) as eluent with a flow rate of 5.0 mL min^{-1}.

Recycling semipreparative high performance liquid chromatography (HPLC) was carried out on a JAI LC-9105, with dichloromethane/hexane, 3/1 (HPLC grade) as eluent with a flow rate of 6.5 mL min^{-1}.

Characterization

Melting points were acquired with an Olympus BX41 polarization microscope (heating rate 5 K min^{-1}) and are uncorrected.

The kinematic viscosity of 1,1,2,2-tetrachloroethane was determined with a Schott AVS 360 Micro-Ubbelohde viscosimeter (501 00, Xylem Analytics Germany GmbH), a transparent thermostat (CT 52) and a Schott heating unit (CK 300). Corrected by the Hagenbach-Couette correction.

The density of 1,1,2,2-tetrachloroethane at 384 K was extracted from literature and extrapolated.[4,5]

^{1}H and ^{13}C NMR spectra were recorded on Bruker Avance DMX 600 and Avance III HD 400 spectrometers. Chemical shifts δ are given in parts per million (ppm) and J (coupling constants)

in Hertz (Hz). For all multiplicities, the following abbreviations were used: s = singlet, d = doublet, dd = doublet of doublets, t = triplet, m = multiplet, br = broad. Solvent signals used for calibration of ^{1}H NMR: in $CDCl_3$ $\delta = 7.26$ ppm, in deuterated 1,1,2,2-tetrachloroethane (TCE-d_2) $\delta = 6.00$ ppm. Solvent signals used for ^{13}C NMR: in $CDCl_3$ $\delta = 77.2$ ppm, in TCE-d_2 $\delta = 73.8$ ppm.

MALDI-TOF mass spectrometry was performed with a Burker Daltonics ultrafleXtreme mass spectrometer in positive-ion mode using *trans*-2-[3-(4-*tert*-butylphenyl)-2-methyl-2-propenylidene]malononitrile (DCTB) as matrix.

Steady-state absorption and fluorescence spectroscopy

Steady state absorption and fluorescence measurements were conducted with spectroscopy grade solvents. Steady-state absorption spectra in toluene (Tol), tetrahydrofuran (THF), DCM (dichloromethane), $CHCl_3$ (chloroform), TCE (1,1,2,2-tetrachloroethane) and benzonitrile (BCN) were measured on a UV/Vis/NIR V-770 spectrophotometer equipped with a JASCO PAC-743R Auto Peltier 6/8-cell changer system for temperature control. Standard Hellma quartz glass cuvettes of different path lengths were used.

Fluorescence studies were carried out on a FLS980 spectrometer from *Edinburgh Instruments*. Fluorescence spectra are spectrally corrected by using correction factor of the fluorescence spectrophotometer. Relative fluorescence quantum yields were measured by the optical dilution method ($A < 0.05$) using *N*,*N*-bis(2,6-diisopropylphenyl)-1,6,7,12-tetraphenoxy-perylene-3,4:9,10-bis(dicarboximide) ($\Phi_{fl} = 96$ % in chloroform) as standard. Absolut fluorescence quantum yields were measured using a Hamamatsu Ulbricht sphere A9924-01 with a continuous Xe lamp and a Hamamatsu photonic multi-channel analyzer C10027 and are reabsorption corrected.

Cyclic and Differential Pulse Voltammetry

Cyclic and differential pulse voltammetry were measured with a standard commercial electrochemical analyser (EC epsilon; BAS Instruments, UK) with a three-electrode single-compartment cell. Tetrabutylammonium hexafluorophosphate ($(n\text{-Bu})_4NPF_6$) was applied as supporting electrolyte with ferrocene (Fc) as an internal standard for the calibration of potentials, Ag/AgCl as reference electrode, Pt disc and Pt wire as working and auxiliary electrodes, respectively. Cyclic voltammetry (CV) and differential pulse voltammetry (DPV) were performed at a scan rate of 100 mV/s at room temperature under argon atmosphere.

Spectroelectrochemistry

Spectroelectrochemical experiments in reflection mode were performed using an Agilent Cary 5000 spectrophotometer in combination with a custom designed sample compartment consisting of a cylindrical PTFE cell with a sapphire window and an adjustable two-in-one electrode (6 mm platinum disc working electrode, 1 mm platinum wire counter electrode). The potentials were adjusted referencing to a silver/silver chloride leak free reference electrode. All experiments were performed at room temperature under an argon atmosphere. The optical path length was adjusted to 0.1 mm and 0.1 M $(n\text{-Bu})_4NPF_6$ was used as supporting electrolyte.

Time-correlated single photon counting

Time-resolved lifetime measurements (TCE) were performed with a ps laser diode at an excitation wavelength of 505 nm and a time-correlated single photon counting (TCSPC) detection unit. Hellma high precision cuvettes with a path length of 1 cm were used.

Femtosecond Transient Absorption

The femtosecond transient absorption (fs-TA) spectrometer consists of an optical parametric amplifier (OPA; Palitra, Quantronix) pumped by a Ti:sapphire regenerative amplifier system (Integra-C, Quantronix) operating at 1 kHz repetition rate and an optical detection system. The generated OPA pulses have a pulse width of ~ 200 fs in the range of 280–2700 nm, which are used as pump pulses. White light continuum (WLC) probe pulses were generated using a sapphire window (4 mm thick) by focusing a small portion of the fundamental 800 nm pulses which was picked off by a quartz plate before entering the OPA. The time delay between pump and probe beams was carefully controlled by making the pump beam travel along a variable optical delay (ILS250, Newport). Intensities of the spectrally dispersed WLC probe pulses are monitored by a High-Speed Spectrometer (Ultrafast Systems) for both visible and near-infrared measurements. To obtain the time-resolved transient absorption difference signal (ΔA) at a specific time, the pump pulses were chopped at 500 Hz and absorption spectra intensities were saved alternately with or without pump pulse. Typically, 4000 pulses excite the samples to obtain the fs-TA spectra at each delay time. The polarization angle between pump and probe beam was set at the magic angle (54.7°) using a Glan-laser polarizer with 4 a half-wave retarder to prevent polarization-dependent signals. The cross-correlation FWHM in pump-probe experiments was around 200 fs and chirp of WLC probe pulses was measured to be 1.2 ps in the 450–800 nm region. To minimize chirp, all reflection optics were used in the probe

beam path. A quartz cell of 2 mm path length was employed. After completing each set of TA experiments, the absorption spectra of all samples were carefully checked to rule out the presence of artifacts or spurious signals arising from, for example, degradation or photo-oxidation of the samples in question. The collected time-dependent differential absorbance $D_{(t,\lambda)}$ was fitted by global analysis using the R-package TIMP software with the graphical interface Glotaran.[6,7] Construction of a rational model describing the population kinetics of the transient species is critical for global analysis of transient absorption data.

2. Synthesis

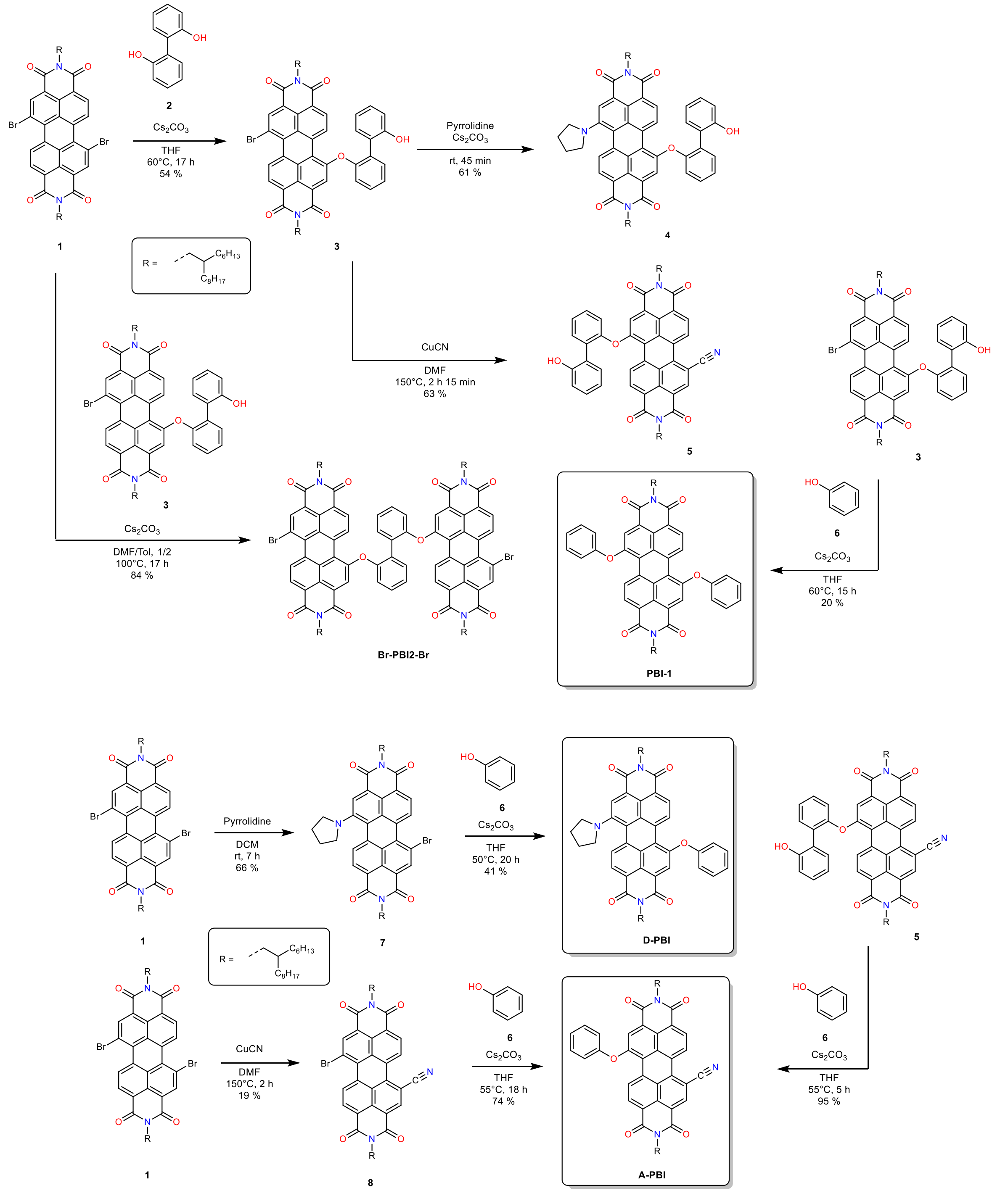

Supplementary Scheme 1. Synthetic procedure. Synthesis of the precursor molecules **4** and **5**, as well as the reference molecules **PBI-1**, **D-PBI** and **A-PBI**.

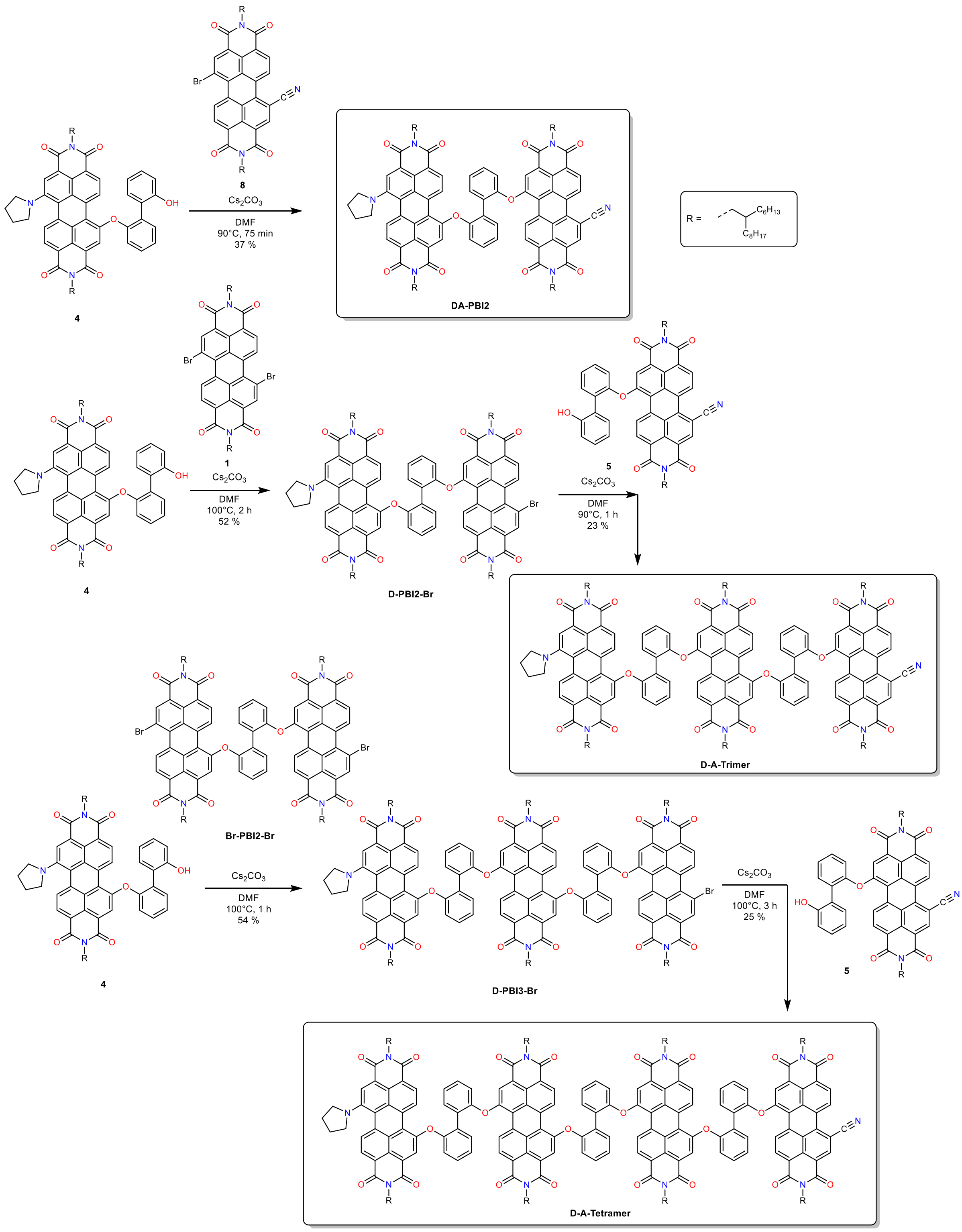

Supplementary Scheme 2. Synthetic procedure. Synthesis of the donor-acceptor molecules **DA-PBI2** to **DA-PBI4**.

_N,N´_-Di(2-hexyldecyl)-1-bromo-7-(2,2´-biphenol)perylene-3,4:9,10-bis(dicarboximide) (3)

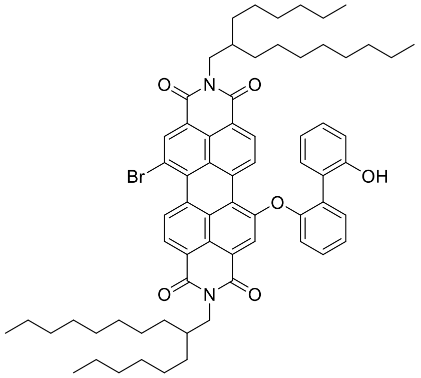

Regioisomerically pure *N,N´*-di(2-hexyldecyl)-1,7-dibromoperylene-3,4:9,10-bis(dicarboximide) (**1**) (20.0 mg, 20.1 µmol), 2,2´-biphenol (**2**) (18.6 mg, 99.9 µmol) and Cs_2CO_3 (29.3 mg, 90.0 µmol) were dissolved in dry THF (8 mL) and the mixture was stirred at 60 °C for 17 h under nitrogen atmosphere. The mixture was cooled to room temperature, H_2O (10 mL) was added and the aqueous layer was extracted with CH_2Cl_2 (5 × 10 mL). The combined organic layers were washed with H_2O (3 × 10 mL) and dried over anhydrous $MgSO_4$. After removal of the solvent under reduced pressure, the crude product was purified by column chromatography on silica gel (CH_2Cl_2/hexane, *v*/*v*, 6/4) to give compound **3** (12.0 mg, 10.9 µmol, 54 %) as a red solid. M.p.: 187 – 188 °C. ^{1}H NMR (400 MHz, TCE-d_2, 295 K): δ/ppm = 9.41 (d, ^3J = 8.0 Hz, 1H), 8.97 (d, ^3J = 8.0 Hz, 1H), 8.78 (s, 1H), 8.50 (d, ^3J = 8.2 Hz, 1H), 8.45 (d, ^3J = 8.2 Hz, 1H), 8.27 (s, 1H), 7.55 (dd, ^3J = 7.3 Hz, ^4J = 2.1 Hz, 1H), 7.48 – 7.39 (m, 2H), 7.28 (dd, ^3J = 7.6 Hz, ^4J = 1.5 Hz, 1H), 7.18 – 7.12 (m, 1H), 7.08 (d, ^3J = 7.9 Hz, 1H), 6.92 – 6.84 (m, 2H), 5.53 (br, 1H), 4.06 (t, ^3J = 7.3 Hz, 4H), 2.00 – 1.92 (m, 2H), 1.43 – 1.20 (m, 48H), 0.88 – 0.82 (m, 12H). ^{13}C NMR (101 MHz, TCE-d_2, 295 K): δ/ppm = 163.4, 163.3, 162.9, 162.6, 155.1, 153.1, 152.5, 137.7, 133.3, 132.7, 132.5, 132.2, 131.2, 130.9, 130.08, 130.06, 129.60, 129.57, 128.7, 128.4, 128.2, 126.7, 125.9, 124.0, 123.9, 123.40, 123.38, 123.1, 122.6, 122.0, 121.9, 121.5, 120.7, 119.7, 115.5, 44.7, 44.6, 36.5, 36.4, 31.8, 31.75, 31.72, 31.49, 31.45, 29.99, 29.97, 29.7, 29.5, 29.2, 26.31, 26.26, 22.6, 14.1. HRMS (MALDI-TOF, positive mode, DCTB in chloroform): m/z calcd. for $C_{68}H_{81}BrN_2O_6$ $[M]^+$: 1100.5278; found: 1100.5242.

***N,N´*-Di(2-hexyldecyl)-1-pyrrolidine-7-(2,2´-biphenol)perylene-3,4:9,10-bis(dicarbox-imide) (4)**

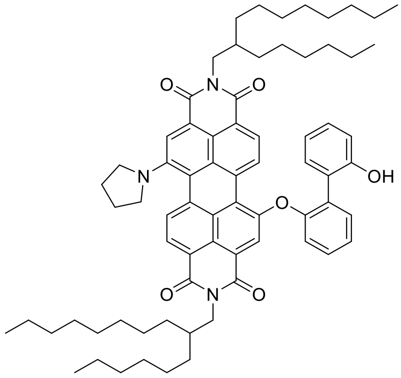

N,N´-Di(2-hexyldecyl)-1-bromo-7-(2,2´-biphenol)perylene-3,4:9,10-bis(dicarboximide) (**3**) (10.0 mg, 9.07 µmol), Cs_2CO_3 (15.0 mg, 46.0 µmol) and pyrrolidine (2 mL) were stirred at room temperature for 45 min under nitrogen atmosphere. Afterwards, H_2O (10 mL) was added and the aqueous layer was extracted with CH_2Cl_2 (3 × 15 mL). The combined organic layers were washed with 0.1 N HCl (2 × 10 mL) (aq.) followed by H_2O (10 mL) and dried over anhydrous $MgSO_4$. After removal of the solvent the crude product was purified by column chromatography on silica (CH_2Cl_2), followed by GPC ($CHCl_3$) and precipitation from CH_2Cl_2 in MeOH to give compound **4** (6.00 mg, 5.49 µmol, 61 %) as a dark green solid. M.p.: 72 – 73 °C. ^{1}H NMR (400 MHz, $CDCl_3$, 295 K): δ/ppm = 8.89 (d, ^3J = 8.1 Hz, 1H), 8.41 (d, ^3J = 8.1 Hz, 1H), 8.33 (s, 1H), 8.25 (s, 1H), 8.21 (d, ^3J = 8.3 Hz, 1H), 7.51 – 7.47 (m, 1H), 7.35 – 7.27 (m, 4H), 7.24 – 7.17 (m, 1H), 7.00 – 6.91 (m, 3H), 6.05 (br, 1H), 4.11 (d, ^3J = 7.3 Hz, 2H), 4.03 (d, ^3J = 7.1 Hz, 2H), 3.69 – 3.54 (m, 2H), 2.81 – 2.54 (m, 2H), 2.14 – 2.02 (m, 2H), 2.00 – 1.89 (m, 4H), 1.41 – 1.19 (m, 48H), 0.86 – 0.82 (m, 12H). ^{13}C NMR (101 MHz, $CDCl_3$, 295 K): δ/ppm = 164.5, 164.3, 164.3, 163.5, 153.9, 153.6, 153.2, 147.9, 134.9, 132.7, 131.5, 131.3, 129.9, 129.8, 129.6, 129.6, 129.3, 129.2, 128.7, 127.0, 125.2, 125.0, 124.8, 124.7, 124.1, 123.5, 122.8, 122.3, 122.1, 121.8, 121.5, 120.7, 118.8, 118.4, 116.2, 52.5, 44.9, 36.9, 36.9, 32.1, 32.1, 31.9, 30.3, 30.3, 30.0, 29.8, 29.8, 29.5, 26.7, 26.7, 25.9, 22.9, 14.3. HRMS (MALDI-TOF, negative mode, DCTB in chloroform): m/z calcd. for $C_{72}H_{89}N_3O_6$ $[M]^-$: 1091.6751; found: 1091.6741.

***N,N´*-Di(2-hexyldecyl)-1-cyano-7-(2,2´-biphenol)perylene-3,4:9,10-bis(dicarboximide) (5)**

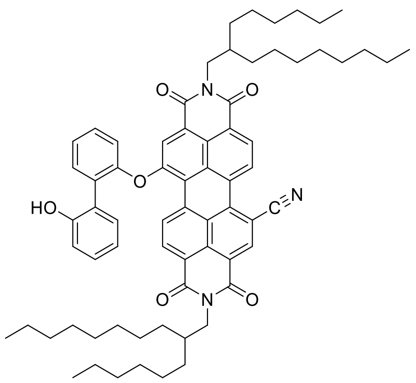

N,N´-Di(2-hexyldecyl)-1-bromo-7-(2,2´-biphenol)perylene-3,4:9,10-bis(dicarboximide) (**3**) (10.0 mg, 9.07 µmol) and copper cyanide (16.3 mg, 0.18 mmol) were dissolved in dry DMF (20 mL) and stirred at 150 °C for 2 h 15 min under nitrogen atmosphere. After the reaction mixture was cooled to room temperature, H_2O (10 mL) was added and the aqueous layer was extracted with CH_2Cl_2 (3 × 15 mL). The combined organic layers were washed with 10 % KOH (2 × 10 mL) (aq.) followed by H_2O (10 mL) and dried over anhydrous $MgSO_4$. After removal of the solvent the crude product was purified by column chromatography on silica (CH_2Cl_2) to give compound **5** (6.00 mg, 5.73 µmol, 63 %) as a dark purple solid. M.p.: 191 – 192 °C. ^{1}H NMR (400 MHz, $CDCl_3$, 295 K): δ/ppm = 9.41 (d, ^3J = 8.2 Hz, 1H), 9.32 (d, ^3J = 8.4 Hz, 1H), 8.74 (s, 1H), 8.62 (d, ^3J = 8.4 Hz, 1H), 8.50 (d, ^3J = 8.2 Hz, 1H), 8.28 (s, 1H), 7.56 – 7.41 (m, 3H), 7.24 – 7.20 (m, 1H), 7.19 – 7.14 (m, 1H), 7.09 – 7.02 (m, 1H), 6.82 – 6.74 (m, 2H), 5.51 (s, 1H), 4.09 (d, ^3J = 7.3 Hz, 2H), 4.04 (d, ^3J = 7.3 Hz, 2H), 2.00 – 1.89 (m, 2H), 1.32 – 1.22 (m, 48H), 0.87 – 0.83 (m, 12H). ^{13}C NMR (101 MHz, $CDCl_3$, 295 K): δ/ppm = 163.2, 163.2, 162.9, 162.5, 156.9, 153.4, 152.4, 137.5, 135.9, 134.0, 133.9, 133.1, 131.1, 130.5, 130.3, 129.9, 129.6, 129.5, 128.3, 128.1, 127.3, 126.6, 124.7, 124.6, 124.5, 124.1, 123.4, 122.6, 121.8, 121.4, 120.8, 120.5, 119.9, 115.8, 106.1, 45.0, 36.8, 36.7, 32.1, 32.0, 32.0, 31.8, 30.2, 30.2, 29.9, 29.8, 29.5, 26.6, 26.6, 22.8, 22.8, 14.3, 14.3. HRMS (MALDI-TOF, negative mode, DCTB in chloroform): m/z calcd. for $C_{69}H_{81}N_3O_6$ $[M]^-$: 1047.6125; found: 1047.6114.

_N,N´_-Di(2-hexyldecyl)-1-bromo-7-cyano-perylene-3,4:9,10-bis(dicarbox imide) (8)

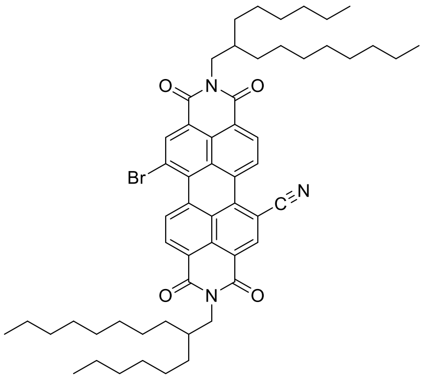

_N,N´_-Di(2-hexyldecyl)-1,7-dibromo-perylene-3,4:9,10-bis(dicarboximide) (**1**) (45.3 mg, 45.4 µmol) and copper cyanide (8.10 mg, 90.4 µmol) were suspended in dry DMF (20 mL) and stirred at 150 °C for 2 h under nitrogen atmosphere. Afterwards the excess CuCN is quenched with 10 % KOH (50 mL) and the reaction mixture is extracted with DCM (50 mL) and brine (50 mL). The organic phase is washed with water (100 mL) and dried over anhydrous $MgSO_4$. The product was purified by column chromatography on silica (DCM) to give compound **8** (10.0 mg, 8.48 µmol, 19 %) as a red solid. M.p.: 150 – 151 °C. ^{1}H NMR (400 MHz, $CDCl_3$, 295 K): δ/ppm = 9.70 (d, ^3J = 8.2 Hz, 1H), 9.47 (d, ^3J = 8.1 Hz, 1H), 8.99 (s, 1H), 8.93 (s, 1H), 8.83 (d, ^3J = 8.2 Hz, 1H), 8.81 (d, ^3J = 8.1 Hz, 1H), 4.16 (d, ^3J = 7.4 Hz, 2H), 4.15 (d, ^3J = 7.3 Hz, 2H), 2.06 – 1.95 (m, 2H), 1.36 – 1.21 (m, 48H), 0.87 – 0.83 (m, 12H). ^{13}C NMR (101 MHz, $CDCl_3$, 295 K): δ/ppm = 163.1, 163.1, 162.6, 162.5, 138.8, 137.2, 135.9, 134.2, 132.7, 132.6, 131.3, 131.2, 129.5, 129.2, 128.7, 128.6, 127.8, 127.4, 125.1, 123.6, 123.3, 123.3, 122.6, 119.5, 107.4, 45.1, 36.8, 32.1, 32.0, 31.8, 30.2, 29.9, 29.8, 29.5, 26.6, 22.9, 22.8, 14.3, 14.3. HRMS (MALDI-TOF, negative mode, DCTB in chloroform): m/z calcd. for $C_{57}H_{72}BrN_3O_4$ $[M]^-$: 941.4706; found: 941.4709.

N,N´-Di(2-hexyldecyl)-1-cyano-7-phenoxy-perylene-3,4:9,10-bis(dicarboximide) (A-PBI)

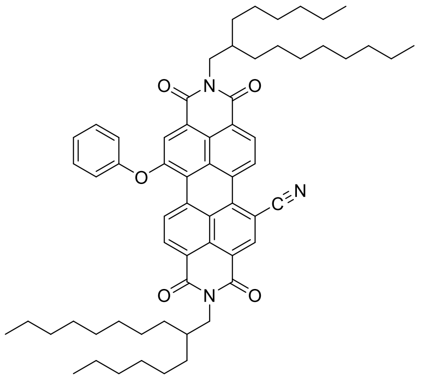

N,N´-Di(2-hexyldecyl)-1-biphenyl-7-cyano-perylene-3,4:9,10-bis(dicarboximide) (**5**) (24.5 mg, 22.4 µmol), Cs_2CO_3 (19.8 mg, 60.7 µmol) and phenol (**6**) (35.0 mg, 372 µmol) were dissolved in dry THF (6.0 mL) and stirred at 55 °C for 5 h under nitrogen atmosphere. After the reaction mixture was cooled to room temperature, H_2O (10 mL) was added and the aqueous layer was extracted with CH_2Cl_2 (3 × 15 mL). The combined organic layers were washed with 0.1 N HCl (2 × 10 mL) (aq.) followed by H_2O (10 mL) and dried over anhydrous $MgSO_4$. After removal of the solvent the crude product was purified by column chromatography on silica (CH_2Cl_2), followed by GPC ($CHCl_3$) and precipitation from CH_2Cl_2 in MeOH to give compound **A-PBI** (20.3 mg, 21.2 µmol, 95 %) as a red solid. M.p.: 167 – 168 °C. ^{1}H NMR (400 MHz, $CDCl_3$, 295 K): δ/ppm = 9.63 (d, ^3J = 8.2 Hz, 1H), 9.58 (d, ^3J = 8.4 Hz, 1H), 8.89 (s, 1H), 8.75 (d, ^3J = 8.4 Hz, 1H), 8.71 (d, ^3J = 8.2 Hz, 1H), 8.30 (s, 1H), 7.55 – 7.48 (m, 2H), 7.37 – 7.31 (m, 1H), 7.24 – 7.19 (m, 2H), 4.12 (d, ^3J = 7.3 Hz, 2H), 4.08 (d, ^3J = 7.3 Hz, 2H), 2.04 – 1.91 (m, 2H), 1.39 – 1.19 (m, 48H), 0.87 – 0.81 (m, 12H). ^{13}C NMR (101 MHz, $CDCl_3$, 295 K): δ/ppm = 163.3, 163.2, 163.0, 162.6, 157.3, 154.5, 137.9, 136.1, 134.2, 134.1, 131.5, 131.0, 129.9, 129.8, 129.3, 128.8, 128.5, 127.7, 126.1, 125.2, 125.0, 124.9, 123.8, 122.9, 122.2, 121.9, 120.3, 120.1, 106.6, 45.1, 45.0, 36.9, 36.7, 32.1, 32.1, 32.0, 32.0, 31.8, 31.8, 30.2, 29.9, 29.9, 29.7, 29.5, 29.5, 26.7, 26.6, 22.8, 22.8, 22.8, 14.3, 14.3. HRMS (MALDI-TOF, negative mode, DCTB in chloroform): m/z calcd. for $C_{63}H_{77}N_3O_5$ $[M]^-$: 955.5863; found: 955.5840.

_N,N´_-Di(2-hexyldecyl)-1-bromo-7-pyrrolidine-perylene-3,4:9,10-bis(dicarboximide) (7)

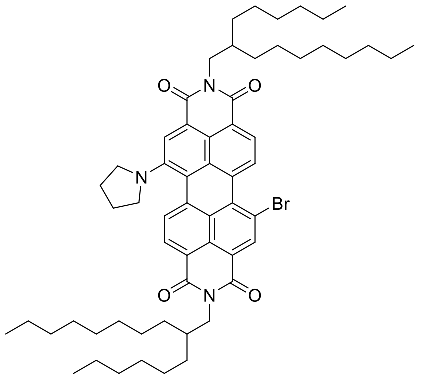

N,N´-Di(2-hexyldecyl)-1,7-dibromo-perylene-3,4:9,10-bis(dicarboximide) (**1**) (43.0 mg, 43.1 µmol) was dissolved in dry DCM (35 mL) and pyrrolidine (3.46 g, 48.7 mmol) was added. To the reaction mixture pyrrolidine (3.46 g, 48.7 mmol) was added over time and stirred at room temperature for 7 h in total under nitrogen atmosphere. Afterwards, 10 % HCL (50 mL) was added and the reaction mixture is extracted with DCM (50 mL). The organic phase was washed with sat. Na_2CO_3 (50 mL) and water (50 mL) and dried over anhydrous $MgSO_4$. The product was purified by column chromatography on silica (DCM) to give compound **7** (28.0 mg, 28.4 µmol, 66 %) as dark green solid. M.p.: 130 – 131 °C. ^{1}H NMR (400 MHz, $CDCl_3$, 295 K): δ/ppm = 9.53 (d, ^3J = 8.3 Hz, 1H), 8.91 (s, 1H), 8.66 (d, ^3J = 8.0 Hz, 1H), 8.55 (s, 1H), 8.49 (d, ^3J = 8.2 Hz, 1H), 7.41 (d, ^3J = 8.0 Hz, 1H), 4.15 (d, ^3J = 7.3 Hz, 2H), 4.14 (d, ^3J = 7.3 Hz, 2H), 3.74 (br, 2H), 2.83 (br, 2H), 2.21 – 2.08 (m, 2H), 2.07 – 1.95 (m, 4H), 1.47 – 1.20 (m, 48H), 0.88 – 0.82 (m, 12H). ^{13}C NMR (101 MHz, $CDCl_3$, 295 K): δ/ppm = 164.5, 164.3, 164.1, 163.4, 148.6, 137.8, 135.1, 134.7, 131.9, 130.9, 130.0, 129.8, 128.3, 127.5, 125.8, 124.2, 123.6, 123.1, 122.6, 121.8, 121.5, 118.5, 117.5, 115.1, 53.0, 44.9, 36.9, 36.8, 32.1, 32.1, 32.0, 31.9, 30.2, 29.9, 29.8, 29.8, 29.5, 26.7, 26.1, 22.9, 14.3, 22.8, 14.3. HRMS (MALDI-TOF, negative mode, DCTB in chloroform): m/z calcd. for $C_{60}H_{80}BrN_3O_4$ $[M]^{-}$: 985.5332; found: 985.5348.

***N,N´*-Di(2-hexyldecyl)-1-phenoxy-7-pyrrolidine-perylene-3,4:9,10-bis(dicarboximide) (D-PBI)**

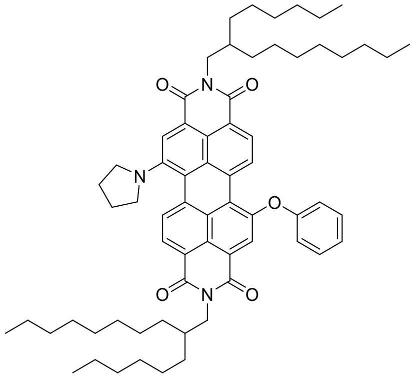

N,N´-Di(2-hexyldecyl)-1-bromo-7-pyrrolidine-perylene-3,4:9,10-bis(dicarbox imide) (**7**) (15.0 mg, 15.2 µmol), Cs_2CO_3 (73.4 mg, 225 µmol) and phenol (**6**) (43.2 mg, 459 µmol) were dissolved in dry THF (2 mL) and stirred at 50 °C for 20 h under nitrogen atmosphere. After the reaction mixture was cooled to room temperature, H_2O (10 mL) was added and the aqueous layer was extracted with CH_2Cl_2 (3 × 15 mL). The combined organic layers were washed with 0.1 N HCl (2 × 10 mL) (aq.) followed by H_2O (10 mL) and dried over anhydrous $MgSO_4$. After removal of the solvent the crude product was purified by column chromatography on silica (CH_2Cl_2), followed by GPC ($CHCl_3$) and precipitation from CH_2Cl_2 in MeOH to give compound **D-PBI** (6.30 mg, 6.30 µmol, 41 %) as a dark green solid. M.p.: 136 – 137 °C. ^{1}H NMR (400 MHz, $CDCl_3$, 295 K): *δ*/ppm = 9.37 (d, ^3J = 8.4 Hz, 1H), 8.62 (d, ^3J = 8.1 Hz, 1H), 8.53 (s, 1H), 8.42 (d, ^3J = 8.4 Hz, 1H), 8.30 (s, 1H), 7.61 (d, ^3J = 8.0 Hz, 1H), 7.45 – 7.40 (m, 2H), 7.24 – 7.20 (m, 1H), 7.19 – 7.15 (m, 2H), 4.13 (d, ^3J = 7.6 Hz, 2H), 4.10 (d, ^3J = 7.6 Hz, 2H), 3.78 (br, 2H), 2.82 (br, 2H), 2.16 – 2.10 (m, 2H), 2.04 – 1.97 (m, 4H), 1.35 – 1.20 (m, 48H), 0.86 – 0.81 (m, 12H). ^{13}C NMR (101 MHz, $CDCl_3$, 295 K): *δ*/ppm = 164.6, 164.5, 164.3, 163.7, 155.8, 153.9, 148.2, 135.6, 132.0, 130.6, 129.9, 129.7, 128.4, 127.3, 125.4, 125.2, 124.9, 124.5, 124.0, 123.1, 122.6, 122.2, 121.9, 119.5, 118.9, 116.5, 52.8, 44.9, 36.9, 32.1, 32.0, 31.9, 30.3, 30.2, 29.9, 29.8, 29.8, 29.5, 26.7, 26.7, 26.0, 22.8, 14.3. HRMS (MALDI-TOF, negative mode, DCTB in chloroform): m/z calcd. for $C_{66}H_{85}N_3O_5$ $[M]^-$: 999.6489; found: 999.6483.

***N,N´*-Di(2-hexyldecyl)-1,7-bisphenoxy-perylene-3,4:9,10-bis(dicarboximide) (PBI-1)**

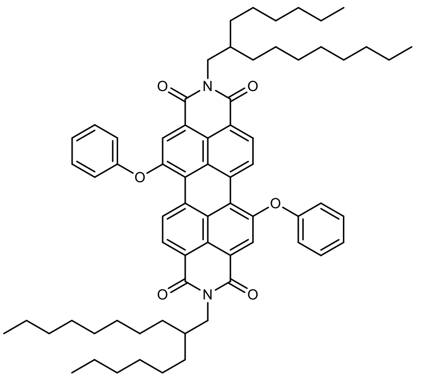

PBI-1 was isolated as a by-product from the reaction of *N,N´*-di(2-hexyldecyl)-1-bromo-7-(2,2´-biphenol)perylene-3,4:9,10-bis(dicarboximide) (**3**) (410 mg, 0.37 mmol), Cs_2CO_3 (1.22 g, 3.74 mmol) and phenol (**6**) (351 mg, 3.73 mmol) in dry THF (10 mL) for 15 h at 60 °C. After cooling to room temperature, H_2O (30 mL) was added and the aqueous layer was extracted with CH_2Cl_2 (3 × 20 mL). After column chromatography of the crude product on silica gel (CH_2Cl_2) and precipitation from CH_2Cl_2 in MeOH, **PBI-1** (76.0 mg, 0.07 mmol, 20 %) was isolated as a dark purple solid. M.p.: 249 – 250 °C. ^{1}H NMR (400 MHz, TCE-d_2, 295 K): δ/ppm = 9.55 (d, ^3J = 8.4 Hz, 2H), 8.56 (d, ^3J = 8.4 Hz, 2H), 8.26 (s, 2H), 7.54 – 7.48 (m, 4H), 7.35 – 7.30 (m, 2H), 7.24 – 7.20 (m, 4H), 4.05 (d, ^3J = 7.1 Hz, 4H), 1.98 – 1.92 (m, 2H), 1.39 – 1.22 (m, 48H), 0.85 – 0.82 (m, 12H). ^{13}C NMR (101 MHz, TCE-d_2, 295 K): δ/ppm = 163.4, 163.1, 155.1, 154.8, 133.1, 130.6, 130.2, 129.0, 128.7, 125.2, 124.8, 123.7, 123.6, 123.5, 121.9, 119.6, 44.6, 36.4, 31.8, 31.7, 31.5, 31.4, 30.0, 29.6, 29.5, 29.2, 26.32, 26.27, 22.58, 22.57, 14.13, 14.11. HRMS (MALDI-TOF, positive mode, DCTB in chloroform): m/z calcd. for $C_{68}H_{82}N_2O_6$ $[M]^+$: 1022.6168; found: 1022.6176.

Bisbromo substituted dimeric PBI (Br-PBI2-Br)

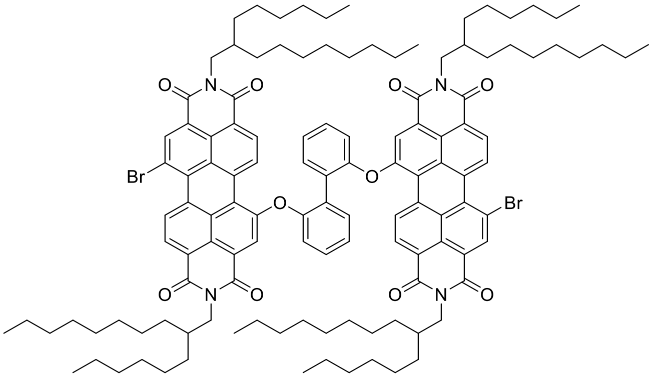

N,N´-Di(2-hexyldecyl)-1-bromo-7-(2,2´-biphenol)perylene-3,4:9,10-bis(dicarboximide) (**3**) (60.0 mg, 54.4 µmol), Cs_2CO_3 (90.2 mg, 282 µmol) and *N,N´*-di(2-hexyldecyl)-1,7-dibromoperylene-3,4:9,10-bis(dicarboximide) (**1**) (163 mg, 163 µmol) were dissolved in dry toluene (100 mL) and stirred for 20 min at room temperature. Afterwards, DMF (50 mL) was added and the reaction mixture was stirred at 100 ° for 17 h under nitrogen atmosphere. After the reaction mixture was cooled to room temperature, H_2O (30 mL) was added and the aqueous layer was extracted with CH_2Cl_2 (3 × 35 mL). The combined organic layers were washed with 0.1 N HCl (2 × 30 mL) (aq.) followed by H_2O (100 mL) and dried over anhydrous $MgSO_4$. After removal of the solvent the crude product was purified by column chromatography on silica (CH_2Cl_2) followed by GPC ($CHCl_3$) to give compound **Br-PBI2-Br** (92.0 mg, 45.6 µmol, 84 %) as a dark red solid. M.p.: 72 – 73 °C. ^{1}H NMR (600 MHz, TCE-d_2, 384 K): δ/ppm = 9.66 (d, ^{3}J = 8.2 Hz, 2H), 8.99 (d, ^{3}J = 8.2 Hz, 2H), 8.97 (s, 2H), 8.57 (d, ^{3}J = 8.2 Hz, 2H), 8.10 (s, 2H), 8.10 (d, ^{3}J = 7.2 Hz, 2H), 7.83 – 7.79 (m, 2H), 7.54 – 7.45 (m, 4H), 7.13 – 7.09 (m, 2H), 4.10 (d, ^{3}J = 7.3 Hz, 4H), 3.77 (d, ^{3}J = 7.3 Hz, 4H), 2.06 – 1.99 (m, 2H), 1.88 – 1.79 (m, 2H), 1.51 – 1.20 (m, 96H), 0.94 – 0.87 (m, 24H). ^{13}C NMR (151 MHz, TCE-d_2, 384 K): δ/ppm = 162.7, 162.6, 162.3, 162.1, 154.7, 152.5, 137.9, 133.4, 132.9, 132.7, 132.3, 130.5, 130.2, 130.1, 129.2, 129.1, 128.3, 127.8, 127.0, 125.6, 124.3, 123.6, 122.9, 122.7, 122.5, 122.4, 121.9, 119.8, 118.7, 44.9, 44.7, 36.6, 36.5, 31.9, 31.9, 31.8, 31.6, 31.6, 31.5, 31.5, 29.8, 29.7, 29.4, 29.3, 29.2, 29.2, 28.9, 28.9, 26.4, 26.4, 26.3, 26.2, 22.3, 22.3, 22.3, 13.6, 13.6, 13.6. HRMS (MALDI-TOF, positive mode, DCTB in chloroform): m/z calcd. for $C_{124}H_{152}Br_2N_4O_{10}$ $[M]^+$, 2018.4060, found: 2017.9864.

Pyrrolidine substituted dimeric PBI (D-PBI2-Br)

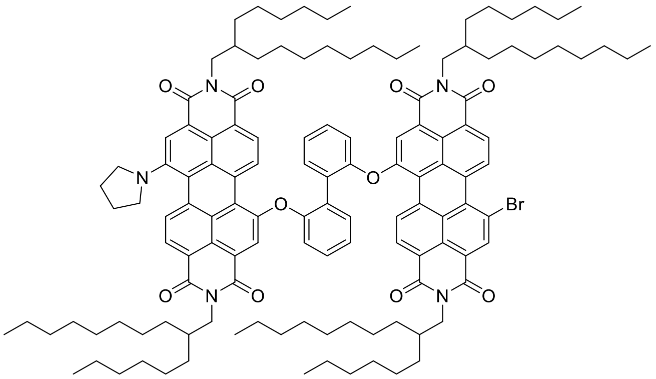

N,N´-Di(2-hexyldecyl)-1-pyrrolidine-7-(2,2´-biphenol)perylene-3,4:9,10-bis(dicarboximide) (**4**) (98.0 mg, 79.7 µmol), Cs_2CO_3 (250 mg, 767 µmol) and *N,N´*-di(2-hexyldecyl)-1,7-dibromoperylene-3,4:9,10-bis(dicarboximide) (**1**) (189 mg, 190 µmol) were dissolved in dry DMF (95 mL) and stirred at 100 ° for 2 h under nitrogen atmosphere. After the reaction mixture was cooled to room temperature, H_2O (50 mL) was added and the aqueous layer was extracted with CH_2Cl_2 (3 × 35 mL). The combined organic layers were washed with 0.1 N HCl (2 × 30 mL) (aq.) followed by H_2O (50 mL) and dried over anhydrous $MgSO_4$. After removal of the solvent the crude product was partially purified by column chromatography on silica (CH_2Cl_2), followed by GPC ($CHCl_3$), HPLC (hexane/ CH_2Cl_2, 1/3) and precipitation from CH_2Cl_2 in MeOH to give compound **D-PBI2-Br** (94.0 mg, 46.8 µmol, 52 %) as a dark green solid. M.p.: 88 – 89 °C. ^{1}H NMR (600 MHz, TCE-d_2, 384 K): δ/ppm = 9.65 (d, ^3J = 8.2 Hz, 1H), 8.97 (d, ^3J = 8.4 Hz, 1H), 8.96 (s, 1H), 8.92 (d, ^3J = 8.3 Hz, 1H), 8.60 (s, 1H), 8.55 (dd, ^4J = 13 Hz, ^3J = 8.1 Hz, 2H), 8.17 (s, 1H), 8.08 (s, 1H), 7.97 (d, ^3J = 8.2 Hz, 1H), 7.83 – 7.77 (m, 4H), 7.54 – 7.41 (m, 4H), 7.16 – 7.09 (m, 2H), 4.11 (d, ^3J = 7.2 Hz, 2H), 4.04 (d, ^3J = 7.2 Hz, 2H), 3.79 (d, ^3J = 7.1 Hz, 2H), 3.74 (d, ^3J = 7.0 Hz, 2H), 3.42 (br, 4H), 2.22 – 2.11 (m, 4H), 2.09 – 1.95 (m, 2H), 1.91 – 1.80 (m, 2H), 1.49 – 1.23 (m, 96H), 0.94 – 0.86 (m, 24H). ^{13}C NMR (151 MHz, TCE-d_2, 384 K): δ/ppm = 163.7, 163.4, 163.1, 162.8, 162.6, 162.5, 162.3, 162.2, 154.9, 153.1, 153.0, 152.6, 147.9, 137.8, 135.2, 133.6, 133.0, 132.6, 132.4, 131.5, 130.6, 130.5, 130.0, 129.9, 129.6, 129.4, 129.3, 128.4, 128.2, 128.0, 127.7, 127.0, 126.3, 125.5, 124.9, 124.8, 124.5, 124.4, 124.0, 123.7, 123.0, 123.0, 122.6, 122.4, 122.4, 122.3, 121.9, 121.8, 121.7, 121.6, 119.7, 118.8, 118.7, 118.3, 117.1, 52.0, 44.8, 44.6, 36.7, 36.6, 36.5, 31.9, 31.9, 31.9, 31.9, 31.8, 31.8, 31.8, 31.6, 31.6, 31.6, 31.5, 31.5, 31.5, 31.5, 29.8, 29.8, , 9.7, 29.5, 29.4, 29.4, 29.4, 29.3, 29.2, 29.2, 29.2, 28.9, 28.9, 26.4, 26.4, 26.4, 26.4, 26.3, 26.3, 26.2, 25.5, 22.3, 22.3, 22.3, 13.7, 13.6. HRMS (MALDI-TOF, negative mode, DCTB in chloroform): m/z calcd. for $C_{128}H_{160}BrN_5O_{10}$ $[M]^-$, 2008.1378, found: 2008.1362.

Pyrrolidine substituted trimeric PBI (D-PBI3-Br)

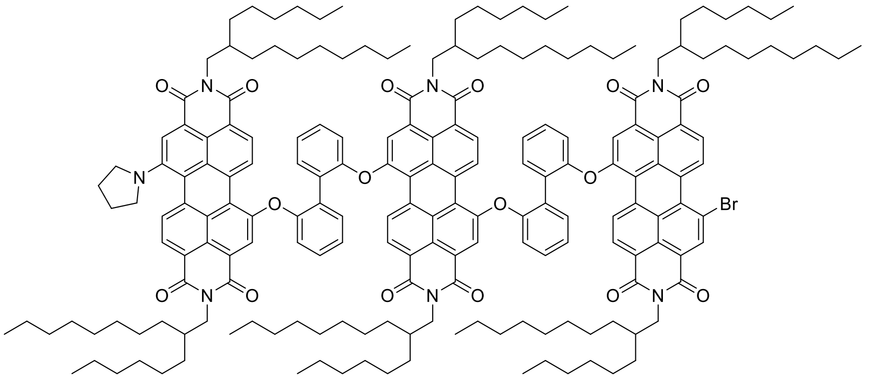

N,N´-Di(2-hexyldecyl)-1-pyrrolidine-7-(2,2´-biphenol)perylene-3,4:9,10-bis(dicarboximide) (**4**) (10.0 mg, 9.15 µmol), Cs_2CO_3 (35.0 mg, 107 µmol) and dibromo substituted dimeric PBI (**Br-PBI2-Br**) (40.0 mg, 19.8 µmol) were dissolved in dry DMF (30 mL) and stirred at 100 ° for 60 min under nitrogen atmosphere. After the reaction mixture was cooled to room temperature, H_2O (20 mL) was added and the aqueous layer was extracted with CH_2Cl_2 (3 × 20 mL). The combined organic layers were washed with 0.1 N HCl (2 × 15 mL) (aq.) followed by H_2O (15 mL) and dried over anhydrous $MgSO_4$. After removal of the solvent the crude product was partially purified by column chromatography on silica (CH_2Cl_2), followed by GPC ($CHCl_3$), HPLC (hexane/ CH_2Cl_2, 1/3) and precipitation from CH_2Cl_2 in MeOH to give **D-PBI3-Br** (15.0 mg, 4.95 µmol, 54 %) as a dark purple solid. M.p.: 110 – 111 °C. ^{1}H NMR (600 MHz, TCE-d_2, 384 K): δ/ppm = 9.60 (d, ^3J = 8.1 Hz, 1H), 9.15 (dd, ^4J = 8.3 Hz, ^3J = 1.9 Hz, 2H), 9.00 (d, ^3J = 8.2 Hz, 1H), 8.93 (s, 1H), 8.92 (d, ^3J = 8.2 Hz, 1H), 8.57 (s, 1H), 8.52 (d, ^3J = 8.1 Hz, 1H), 8.50 (d, ^3J = 8.1 Hz, 1H), 8.13 (s, 1H), 8.10 – 8.08 (m, 2H), 8.07 – 8.04 (m, 4H), 7.85 – 7.80 (m, 5H), 7.75 (d, ^3J = 8.0 Hz, 1H), 7.58 – 7.55 (m, 1H), 7.53 – 7.46 (m, 7H), 7.34 – 7.29 (m, 1H), 7.28 – 7.23 (m, 1H), 7.18 – 7.13 (m, 2H), 4.04 (d, ^3J = 7.2 Hz, 2H), 4.01 (d, ^3J = 7.2 Hz, 2H), 3.84 – 3.75 (m, 4H), 3.62 (d, ^3J = 7.1 Hz, 2H), 3.53 (d, ^3J = 6.8 Hz, 2H), 3.41 (br, 4H), 2.19 – 2.11 (m, 4H), 2.04 – 1.94 (m, 2H), 1.94 – 1.84 (m, 2H), 1.76 – 1.67 (m, 2H), 1.34 – 1.19 (m, 144H), 0.93 – 0.86 (m, 36H). ^{13}C NMR (151 MHz, TCE-d_2, 384 K): δ/ppm = 163.6, 163.3, 163.2, 162.7, 162.6, 162.6, 162.5, 162.4, 162.2, 162.2,162.0, 155.1, 154.5, 154.3, 153.4, 153.0, 152.7, 152.5, 147.6, 137.8, 135.2, 133.5, 133.2, 133.0, 132.9, 132.8, 132.6, 132.5, 132.5, 132.4, 131.6, 130.8, 130.6, 130.5, 130.3, 130.2, 130.1, 130.1, 129.9, 129.9, 129.6, 129.5, 129.2, 29.2, 129.1, 129.0, 129.0, 129.0, 128.2, 128.2, 128.1, 128.0, 127.5, 127.3, 126.9, 126.7, 126.5, 125.8, 125.3, 125.2, 125.2, 124.7, 124.4, 124.3, 124.3, 124.0, 123.8, 123.5, 123.0, 122.9, 122.8, 122.7, 122.6, 122.4, 122.3, 122.3, 122.3, 122.1, 122.0, 121.7, 121.6, 121.5, 121.5, 121.3, 119.6, 119.3, 119.0, 119.0, 118.9, 118.8, 117.2, 51.9,

44.7, 44.6, 44.5, 44.3, 36.6, 36.6, 36.6, 36.5, 36.5, 36.5, 31.8, 31.8, 31.8, 31.7, 31.7, 31.7, 31.6, 31.6, 31.6, 31.5, 31.5, 31.5, 31.5, 31.4, 29.8, 29.8, 29.8, 29.7, 29.4, 29.4, 29.4, 29.3, 29.3, 29.2, 29.2, 29.2, 29.2, 29.2, 29.2, 28.9, 28.9, 26.4, 26.4, 26.3, 26.3, 26.3, 26.2, 26.2, 26.1, 25.5, 22.4, 22.3, 22.3, 22.3, 22.2, 13.7, 13.6, 13.6. HRMS (MALDI-TOF, negative mode, DCTB in chloroform): m/z calcd. for $C_{196}H_{240}BrN_7O_{16}$ $[M]^-$, 3029.7422, found: 3029.7397.

DA-PBI2

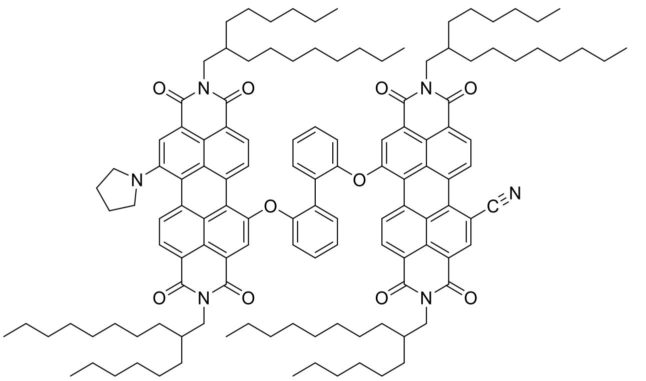

N,N′-Di(2-hexyldecyl)-1-bromo-7-cyano-perylene-3,4:9,10-bis(dicarboximide) (**8**) (8.00 mg, 8.48 µmol), Cs_2CO_3 (16.7 mg, 51.3 µmol) and *N,N′*-Di(2-hexyldecyl)-1-pyrrolidine-7-(2,2′-biphenol)perylene-3,4:9,10-bis(dicarboximide) (**4**) (24.7 mg, 22.6 µmol) were dissolved in dry DMF (4 mL) and stirred at 90 ° for 75 min under nitrogen atmosphere. After the reaction mixture was cooled to room temperature, H_2O (10 mL) was added and the aqueous layer was extracted with CH_2Cl_2 (3 × 15 mL). The combined organic layers were washed with 0.1 N HCl (2 × 10 mL) (aq.) followed by H_2O (10 mL) and dried over anhydrous $MgSO_4$. After removal of the solvent the crude product was partially purified by column chromatography on silica (CH_2Cl_2), followed by repeating cycles of GPC ($CHCl_3$), HPLC (hexane/ CH_2Cl_2, 1/3) and precipitation from CH_2Cl_2 in MeOH to give the pure compound **DA-PBI2** (6.10 mg, 3.12 µmol, 37 %) as a dark purple solid. M.p.: 85 – 86 °C. ^{1}H NMR (600 MHz, TCE-d_2, 384 K): δ/ppm = 9.66 (d, ^3J = 8.1 Hz, 1H), 9.19 (d, ^3J = 8.3 Hz, 1H), 8.94 (s, 1H), 8.92 (d, ^3J = 8.2 Hz, 1H), 8.64 (d, ^3J = 8.1 Hz, 1H), 8.60 (s, 1H), 8.52 (d, ^3J = 8.0 Hz, 1H), 8.19 (s, 1H), 8.18 (d, ^3J = 8.2 Hz, 1H), 8.02 (s, 1H), 7.86 – 7.83 (m, 1H), 7.82 (d, ^3J = 8.2 Hz, 1H), 7.80 – 7.76 (m, 2H), 7.58 – 7.51 (m, 2H), 7.47 – 7.41 (m, 2H), 7.19 – 7.16 (m, 1H), 7.10 – 7.07 (m, 1H), 4.13 (d, ^3J = 7.3 Hz, 2H), 4.09 (d, ^3J = 7.3 Hz, 2H), 3.77 (d, ^3J = 7.3 Hz, 2H), 3.69 (d, ^3J = 7.0 Hz, 2H), 3.41 (br, 4H), 2.21 – 2.11 (m, 4H), 2.09 – 1.99 (m, 2H), 1.90 – 1.78 (m, 2H), 1.42 – 1.21 (m, 96H), 0.93 – 0.87 (m, 24H). ^{13}C NMR (151 MHz, TCE-d_2, 384 K): δ/ppm = 163.7, 163.5, 163.0, 162.5, 162.4, 162.4, 162.0, 161.8, 156.1, 152.9, 152.1, 147.9, 137.4, 135.5, 135.2, 133.7, 132.9, 132.5, 131.5, 131.3, 130.7, 130.1, 129.8, 129.6, 129.4, 129.4, 129.0, 128.5,

128.4, 127.9, 127.6, 127.5, 126.3, 125.9, 125.0, 124.8, 124.5, 124.4, 124.4, 124.3, 124.0, 123.0, 122.7, 122.4, 122.2, 121.9, 121.8, 121.7, 121.5, 119.6, 119.1, 118.8, 118.4, 116.9, 106.4, 52.0, 44.9, 44.8, 44.6, 44.5, 36.7, 36.6, 36.5, 36.5, 31.9, 31.9, 31.9, 31.8, 31.8, 31.7, 31.6, 31.6, 31.5, 31.5, 31.5, 31.4, 29.8, 29.8, 29.8, 29.7, 29.4, 29.4, 29.4, 29.3, 29.2, 29.2, 29.2, 29.2, 28.9, 28.9, 28.9, 26.4, 26.4, 26.4, 26.3, 26.3, 26.2, 26.2, 25.5, 22.3, 22.3, 22.3, 13.6, 13.6, 13.6. HRMS (MALDI-TOF, negative mode, DCTB in chloroform): m/z calcd. for $C_{129}H_{160}N_6O_{10}$ [M]$^-$, 1954.2234, found: 1954.2185.

DA-PBI3

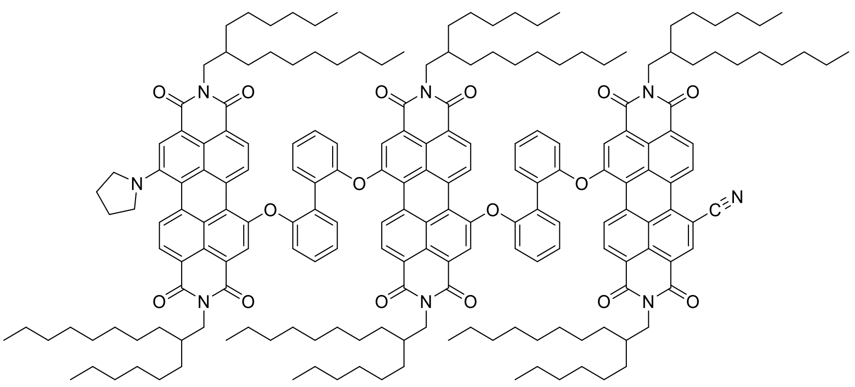

N,N´-Di(2-hexyldecyl)-1-cyano-7-(2,2´-biphenol)perylene-3,4:9,10-bis(dicarboximide) (**5**) (15.0 mg, 14.3 µmol), Cs_2CO_3 (56.0 mg, 172 µmol) and pyrrolidine substituted dimeric PBI (**D-PBI2-Br**) (36.0 mg, 17.9 µmol) were dissolved in dry DMF (5 mL) and stirred at 90 ° for 60 min under nitrogen atmosphere. After the reaction mixture was cooled to room temperature, H_2O (10 mL) was added and the aqueous layer was extracted with CH_2Cl_2 (3 × 15 mL). The combined organic layers were washed with 0.1 N HCl (2 × 10 mL) (aq.) followed by H_2O (10 mL) and dried over anhydrous $MgSO_4$. After removal of the solvent the crude product was partially purified by column chromatography on silica (CH_2Cl_2), followed by repeating cycles of GPC ($CHCl_3$), HPLC (hexane/ CH_2Cl_2, 1/3) and precipitation from CH_2Cl_2 in MeOH to give the pure compound **DA-PBI3** (10.0 mg, 3.36 µmol, 23 %) as a dark purple solid. M.p.: 104 – 105 °C. ^{1}H NMR (600 MHz, TCE-d_2, 384 K): δ/ppm = 9.59 (d, ^3J = 8.1 Hz, 1H), 9.19 (d, ^3J = 8.3 Hz, 1H), 9.13 (dd, ^4J = 8.3 Hz, ^3J = 4.3 Hz, 2H), 8.90 (d, ^3J = 8.1 Hz, 1H), 8.90 (s, 1H), 8.58 (d, ^3J = 8.1 Hz, 1H), 8.56 (s, 1H), 8.49 (d, ^3J = 7.9 Hz, 1H), 8.27 (d, ^3J = 7.6 Hz, 1H), 8.13 (s, 1H), 8.10 (s, 1H), 8.08 (d, ^3J = 8.3 Hz, 1H), 8.04 (d, ^3J = 8.3 Hz, 1H), 8.01 (s, 1H), 7.98 (s, 1H), 7.86 – 7.79 (m, 5H), 7.74 (d, ^3J = 8.0 Hz, 1H), 7.61 – 7.58 (m, 1H), 7.55 – 7.43 (m, 7H), 7.32 – 7.31 (m, 1H), 7.26 – 7.24 (m, 1H), 7.19 (d, ^3J = 7.7 Hz, 1H), 7.12 (d, ^3J = 7.7 Hz, 1H), 4.06 (d, ^3J = 7.3 Hz, 2H), 4.04 (d, ^3J = 7.5 Hz, 2H), 3.78 (d, ^3J = 6.9 Hz, 2H), 3.71 (d, ^3J = 6.9 Hz, 2H), 3.59 (d, ^3J = 6.9 Hz, 2H), 3.52 (d, ^3J = 6.9 Hz, 2H), 3.40 (br, 4H),

2.17 – 2.11 (m, 4H), 2.04 – 1.96 (m, 2H), 1.91 – 1.84 (m, 1H), 1.84 – 1.78 (m, 1H), 1.75 – 1.61 (m, 2H), 1.34 – 1.21 (m, 144H), 0.91 – 0.86 (m, 36H). ^{13}C NMR (151 MHz, TCE-d_2, 384 K): δ/ppm = 163.7, 163.3, 163.1, 162.6, 162.6, 162.4, 162.3, 162.3, 162.0, 161.9, 161.9, 161.8, 156.3, 154.6, 154.2, 153.3, 153.0, 152.5, 152.4, 151.9, 147.7, 137.3, 135.5, 135.2, 133.7, 133.1, 133.0, 132.9, 132.7, 132.6, 132.5, 131.6, 131.1, 130.9, 130.5, 130.4, 130.3, 130.2, 130.1, 130.0, 129.7, 129.6, 129.4, 129.4, 129.3, 129.2, 129.0, 128.4, 128.1, 128.0, 127.8, 127.3, 127.3, 126.4, 126.3, 125.3, 125.2, 124.7, 124.5, 124.3, 124.3, 124.0, 123.5, 122.9, 122.9, 122.8, 122.7, 122.6, 122.4, 122.3, 122.1, 122.0, 121.6, 121.5, 121.3, 121.3, 120.2, 119.7, 119.5, 119.1, 118.9, 118.8, 117.1, 106.2, 51.9, 44.8, 44.6, 44.5, 44.3, 36.6, 36.6, 36.5, 31.8, 31.8, 31.7, 31.7, 31.6, 31.6, 31.6, 31.5, 31.5, 31.5, 31.5, 31.5, 31.5, 31.4, 31.4, 29.8, 29.8, 29.7, 29.4, 29.4, 29.4, 29.4, 29.4, 29.3, 29.3, 29.2, 29.2, 29.2, 29.2, 29.2, 29.0, 28.9, 28.9, 28.9, 26.4, 26.3, 26.3, 26.3, 26.3, 26.2, 26.1, 26.1, 26.1, 25.5, 22.3, 22.3, 22.3, 22.3, 22.2, 22.2, 13.7, 13.6, 13.6. HRMS (MALDI-TOF, negative mode, DCTB in chloroform): m/z calcd. for $C_{197}H_{240}N_8O_{16}$ $[M]^-$, 2975.8284, found: 2975.8302.

DA-PBI4

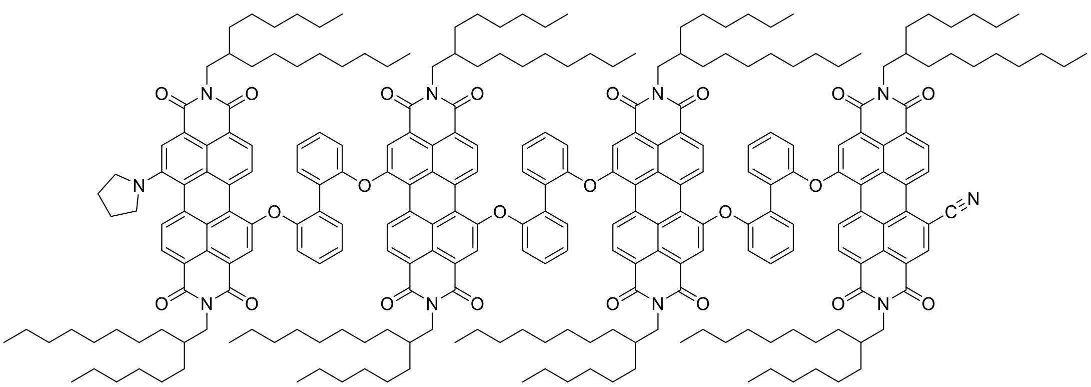

N,N′-Di(2-hexyldecyl)-1-cyano-7-(2,2′-biphenol)perylene-3,4:9,10-bis(dicarboximide) (**5**) (11.0 mg, 10.5 µmol), Cs_2CO_3 (24.0 mg, 73.7 µmol) and the pyrrolidine substituted trimeric PBI (**D-PBI3-Br**) (15.0 mg, 4.95 µmol) were dissolved in dry DMF (3 mL) and stirred at 100 °C for 3 h under nitrogen atmosphere. After the reaction mixture was cooled to room temperature, H_2O (10 mL) was added and the aqueous layer was extracted with CH_2Cl_2 (3 × 15 mL). The combined organic layers were washed with 0.1 N HCl (2 × 10 mL) (aq.) followed by H_2O (10 mL) and dried over anhydrous $MgSO_4$. After removal of the solvent the crude product was partially purified by column chromatography on silica (CH_2Cl_2), followed by repeating cycles of GPC ($CHCl_3$), HPLC (hexane/ CH_2Cl_2, 1/3) and precipitation from CH_2Cl_2 in MeOH to give the pure compound **DA-PBI4** (5.00 mg, 1.25 µmol, 25 %) as a dark purple solid. M.p.: 111 – 112 °C. ^{1}H NMR (600 MHz, TCE-d_2, 384 K): δ/ppm = 9.59 (d, ^3J = 8.0 Hz, 1H),

9.20 – 9.08 (m, 5H), 8.91 (s, 1H), 8.89 (d, ^3J = 8.2 Hz, 1H), 8.58 (d, ^3J = 8.1 Hz, 1H), 8.56 (s, 1H), 8.47 (d, ^3J = 8.0 Hz, 1H), 8.22 (d, ^3J = 7.0 Hz, 1H), 8.15 (d, ^3J = 8.5 Hz, 1H), 8.12 (s, 1H), 8.09 (d, ^3J = 7.7 Hz, 1H), 8.06 – 8.02 (m, 3H), 8.00 – 7.95 (m, 4H), 7.85 – 7.73 (m, 8H), 7.56 – 7.46 (m, 10H), 7.43 – 7.33 (m, 3H), 7.32 – 7.26 (m, 2H), 7.26 – 7.22 (m, 1H), 7.14 – 7.11 (m, 1H), 7.08 – 7.03 (m, 1H), 4.08 (d, ^3J = 7.4 Hz, 2H), 4.05 (d, ^3J = 7.3 Hz, 2H), 3.78 – 3.73 (m, 2H), 3.70 – 3.65 (m, 2H), 3.60 – 3.54 (m, 4H), 3.54 – 3.48 (m, 4H), 3.38 (br, 4H), 2.16 – 2.09 (m, 4H), 2.05 – 1.98 (m, 2H), 1.89 – 1.83 (m, 1H), 1.82 – 1.75 (m, 1H), 1.74 – 1.65 (m, 4H), 1.34 – 1.17 (m, 192H), 0.92 – 0.84 (m, 48H). ^{13}C NMR (151 MHz, TCE-*d*$_2$, 384 K): *δ*/ppm = 163.7, 163.4, 163.1, 162.6, 162.5, 162.5, 162.4, 162.4, 162.3, 162.0, 162.0, 161.9, 161.8, 161.7, 156.2, 154.7, 154.4, 154.2, 153.3, 152.9, 152.5, 152.3, 152.2,152.0, 151.8, 147.7, 137.2, 135.5, 135.2, 133.7, 133.2, 133.1, 133.0, 132.8, 132.7, 132.6, 132.4, 131.6, 131.1, 130.7, 130.5, 130.4, 130.3, 130.3, 130.3, 130.1, 130.0, 129.9, 129.8, 129.6, 129.5, 129.4, 129.3, 129.2, 129.2, 129.1, 129.0, 128.9, 128.9, 128.4, 128.0, 128.0, 127.8, 127.4, 127.3, 126.4, 126.2, 125.5, 125.4, 125.3, 125.0, 124.7, 124.4, 124.4, 124.4, 124.3, 124.3, 124.2, 124.0, 123.2, 123.0, 122.9, 122.8, 122.7, 122.6, 122.4, 122.2, 122.1, 122.1, 121.9, 121.7, 121.6, 121.5, 121.4, 121.4, 121.3, 120.2, 119.7, 119.6, 119.5, 119.2, 119.1, 118.9, 118.7, 117.1, 106.3, 51.9, 44.9, 44.7, 44.6, 44.4, 44.3, 44.3, 36.6, 36.6, 36.5, 36.5, 31.9, 31.9, 31.8, 31.8, 31.8, 31.7, 31.7, 31.6, 31.6, 31.6, 31.6, 31.5, 31.5, 31.5, 31.5, 31.4, 31.4, 31.4, 29.8, 29.8, 29.8, 29.7, 29.7, 29.7, 29.4, 29.4, 29.4, 29.3, 29.3, 29.3, 29.3, 29.2, 29.2, 29.2, 29.2, 29.1, 28.9, 28.9, 28.9, 26.4, 26.4, 26.4, 26.3, 26.3, 26.3, 26.2, 26.2, 26.2, 26.1, 26.1, 26.1, 25.5, 22.3, 22.3, 22.3, 22.3, 22.2, 13.7, 13.6, 13.6, 13.6. HRMS (MALDI-TOF, negative mode, DCTB in chloroform): m/z calcd. for $C_{265}H_{320}N_{10}O_{22}$ [M]$^-$, 3996.4300, found: 3996.4326.

3. 2D NMR Spectroscopy

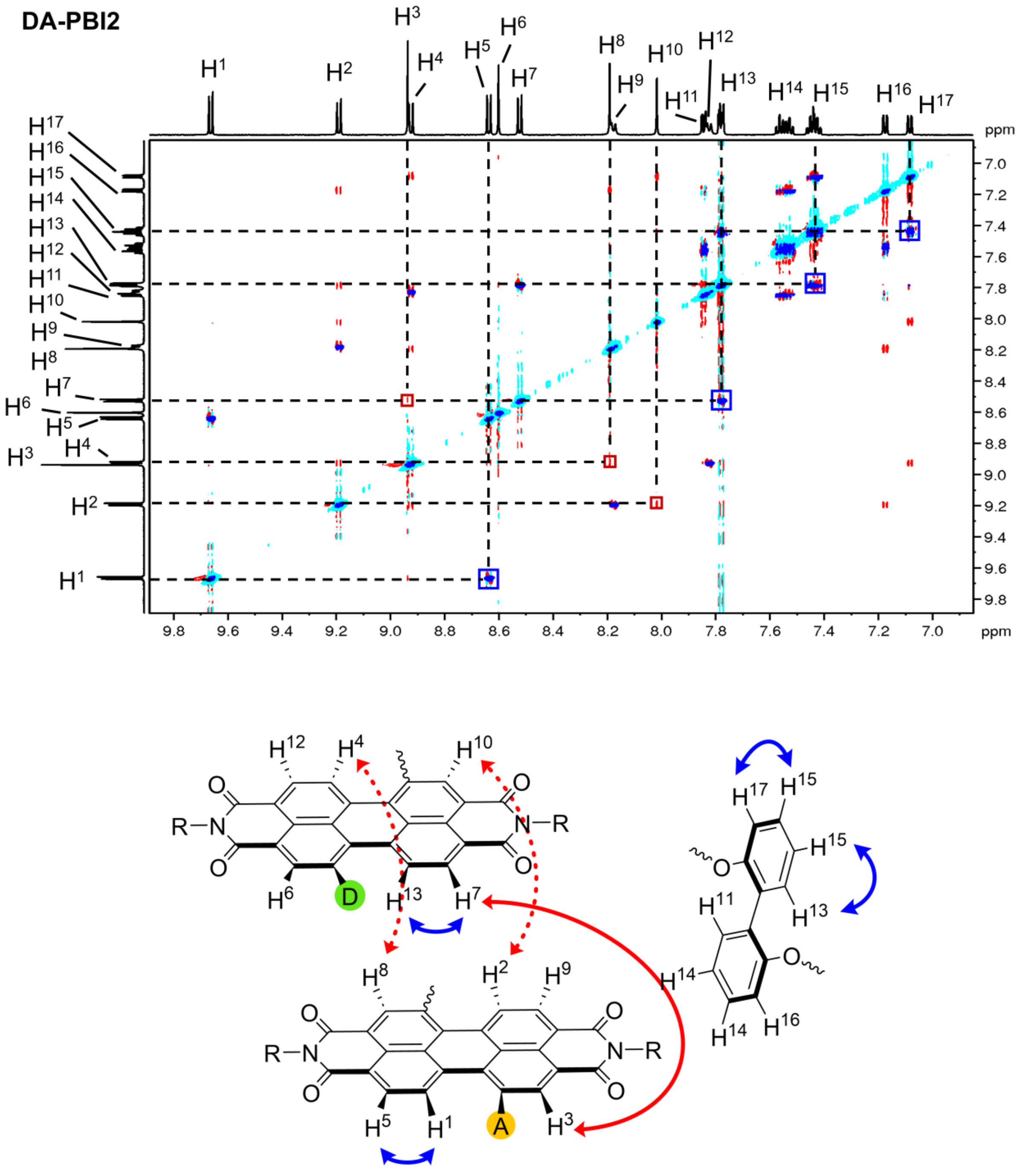

Supplementary Figure 1. 2D NMR spectroscopic characterization. Superposition of COSY (blue) and ROESY (red: positive signal, cyan: negative signal) spectra (600 MHz, 384 K, TCE-d_2) of **DA-PBI2**. Red boxes / arrows indicate important ROESY cross-signals, blue boxes / arrows indicate important COSY signals.

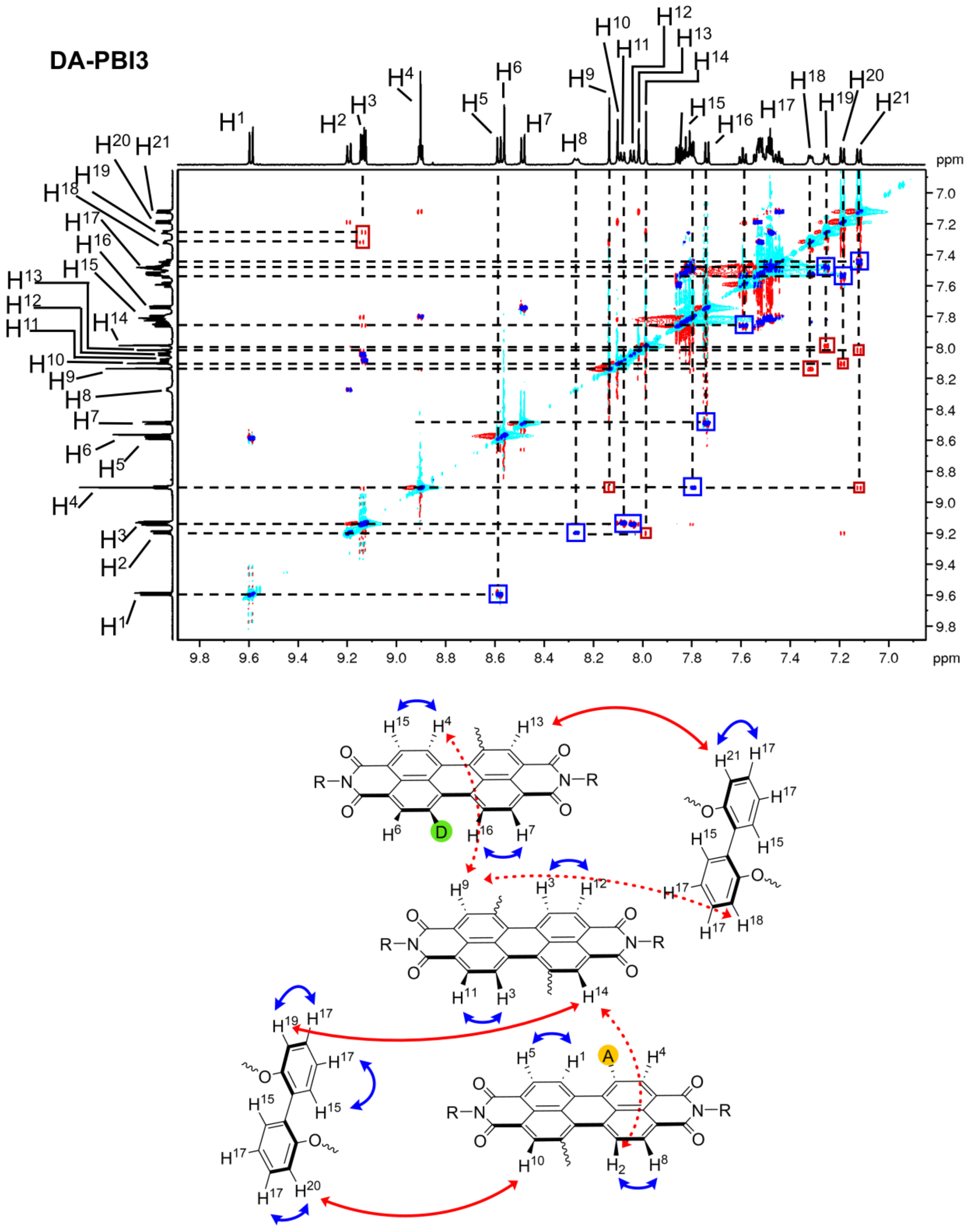

Supplementary Figure 2. 2D NMR spectroscopic characterization. Superposition of COSY (blue) and ROESY (red: positive signal, cyan: negative signal) spectra (600 MHz, 384 K, TCE-d_2) of **DA-PBI3**. Red boxes / arrows indicate important ROESY cross-signals, blue boxes / arrows indicate important COSY signals.

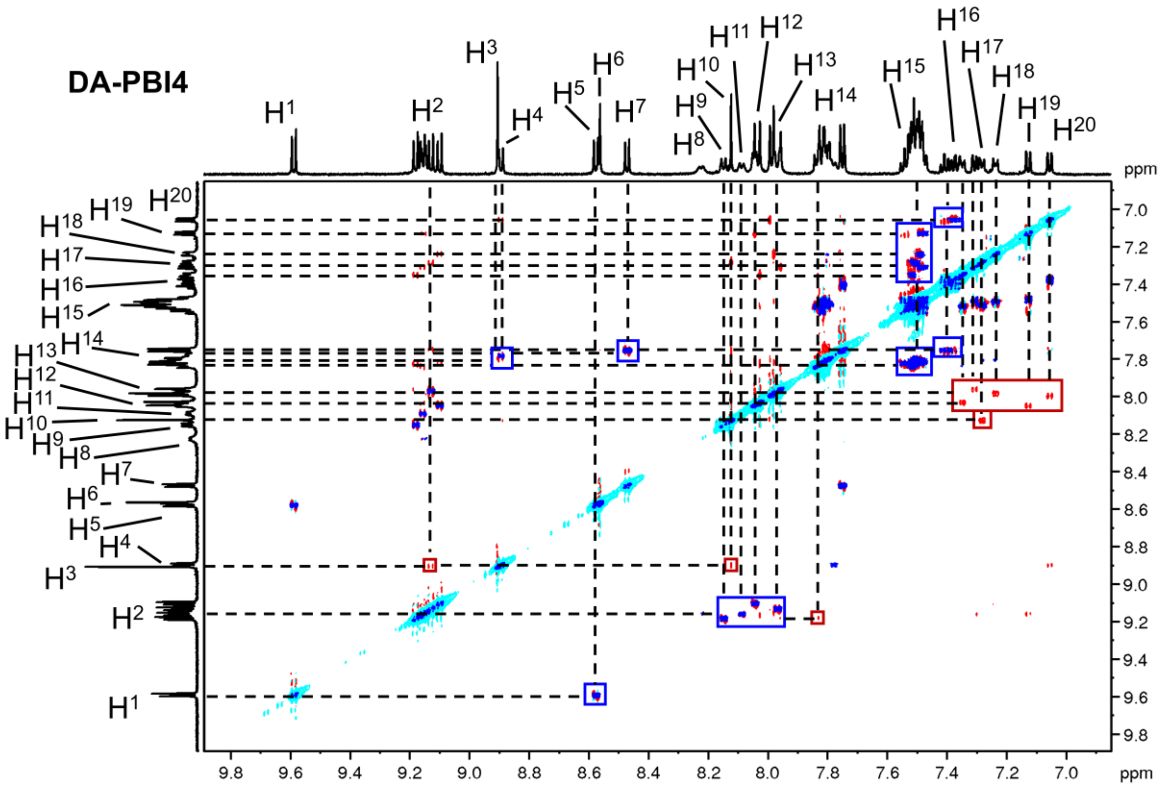

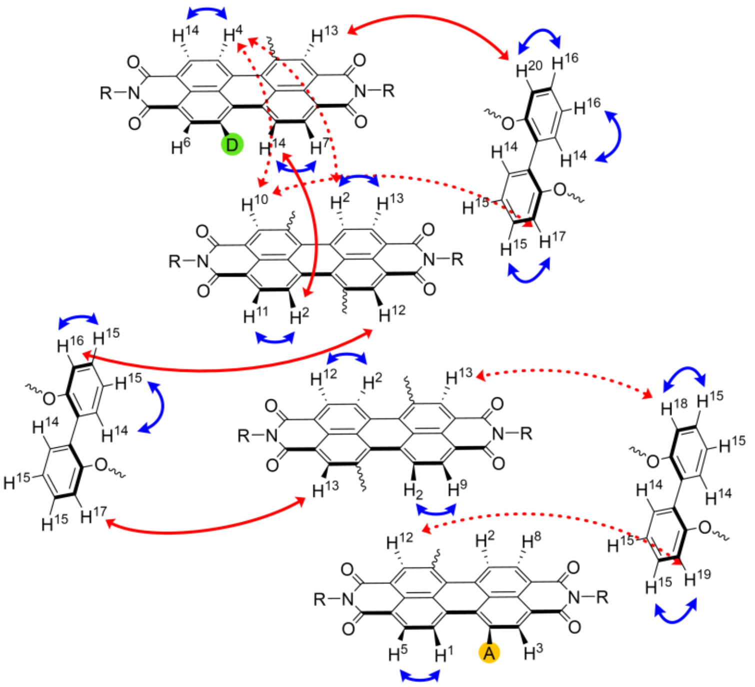

Supplementary Figure 3. 2D NMR spectroscopic characterization. Superposition of COSY (blue) and ROESY (red: positive signal, cyan: negative signal) spectra (600 MHz, 384 K, TCE-d_2) of **DA-PBI4**. Red boxes / arrows indicate important ROESY cross-signals, blue boxes / arrows indicate important COSY signals.

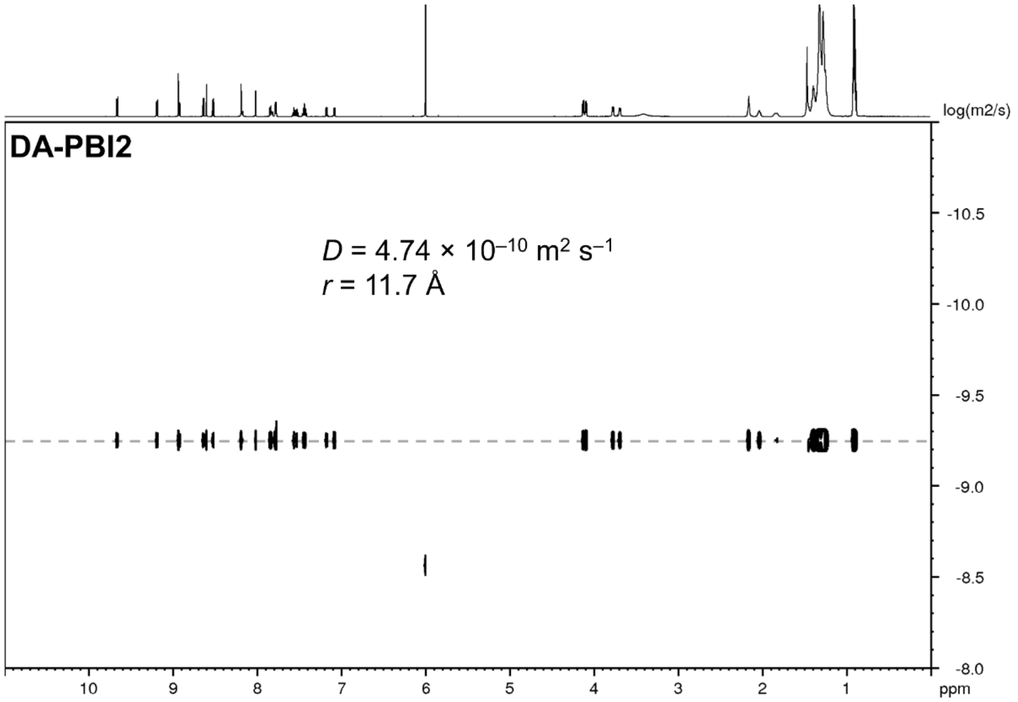

Supplementary Figure 4. NMR spectroscopic characterization. 2D plot of the DOSY NMR (600 MHz, 384 K, TCE-d_2) spectra of **DA-PBI2** with the diffusion coefficient and hydrodynamic radii as received from the Stokes-Einstein equation.[8,9]

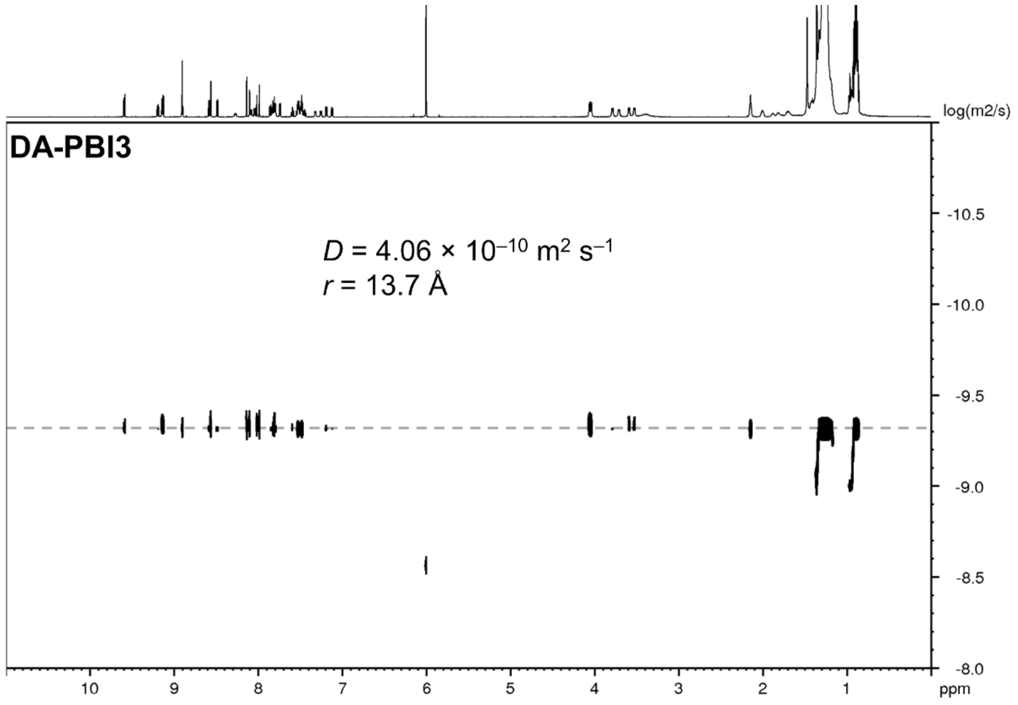

Supplementary Figure 5. NMR spectroscopic characterization. 2D plot of the DOSY NMR (600 MHz, 384 K, TCE-d_2) spectra of **DA-PBI3** with the diffusion coefficient and hydrodynamic radii as received from the Stokes-Einstein equation.[8,9]

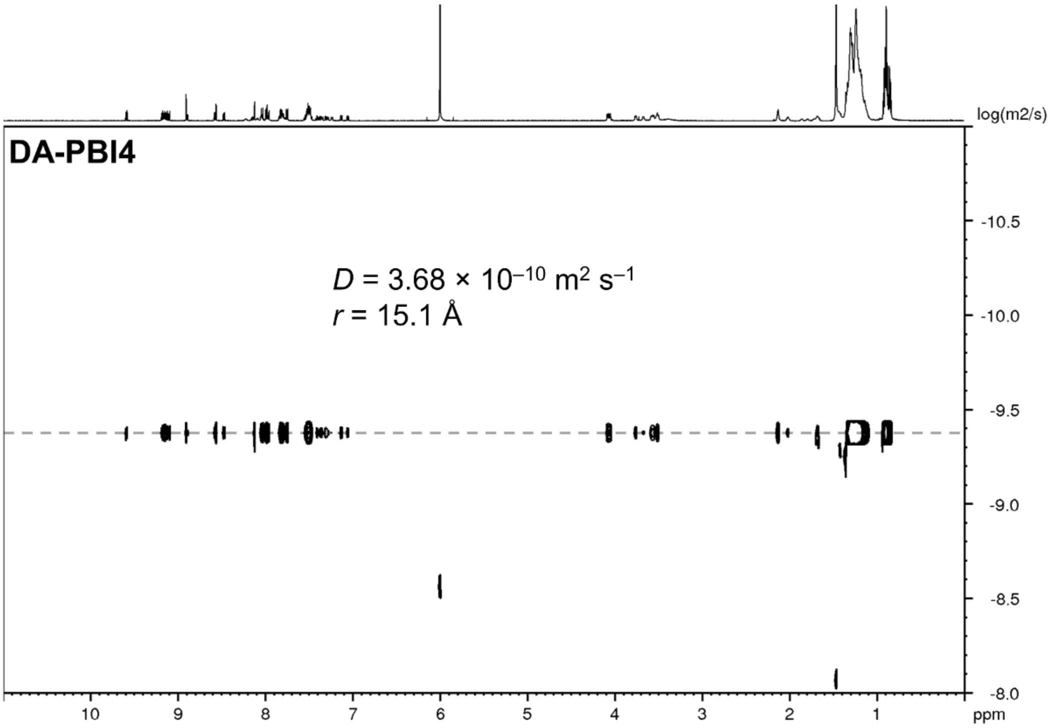

Supplementary Figure 6. NMR spectroscopic characterization. 2D plot of the DOSY NMR (600 MHz, 384 K, TCE-d_2) spectra of **DA-PBI4** with the diffusion coefficient and hydrodynamic radii as received from the Stokes-Einstein equation.[8,9]

4. Quantum Chemical and Weller Calculations

Geometry optimizations were performed for **DA-PBI2** to **DA-PBI4** at the density functional theory (DFT) level, as implemented in the Gaussian 16 program package[10], using the long-range corrected hybrid density wB97X-D[11] functional including dispersion correction and the def2-SVP basis set.[12] An implicit solvent simulation for Tol and THF using the polarizable continuum model (PCM) implemented in Gaussian16 was applied. The structures were geometry-optimized followed by frequency calculations to prove the existence of true minima. The solubilizing alkyl chains were replaced by methyl groups to reduce the computational effort. This series exhibits PBI units at equidistant center-to-center offsets of 4.5 Å and longitudinal slip angles of approximately 47 – 48°, accompanied by a rotational offset ranging from 5 to 7° and a transverse slip of ~ 0 Å.

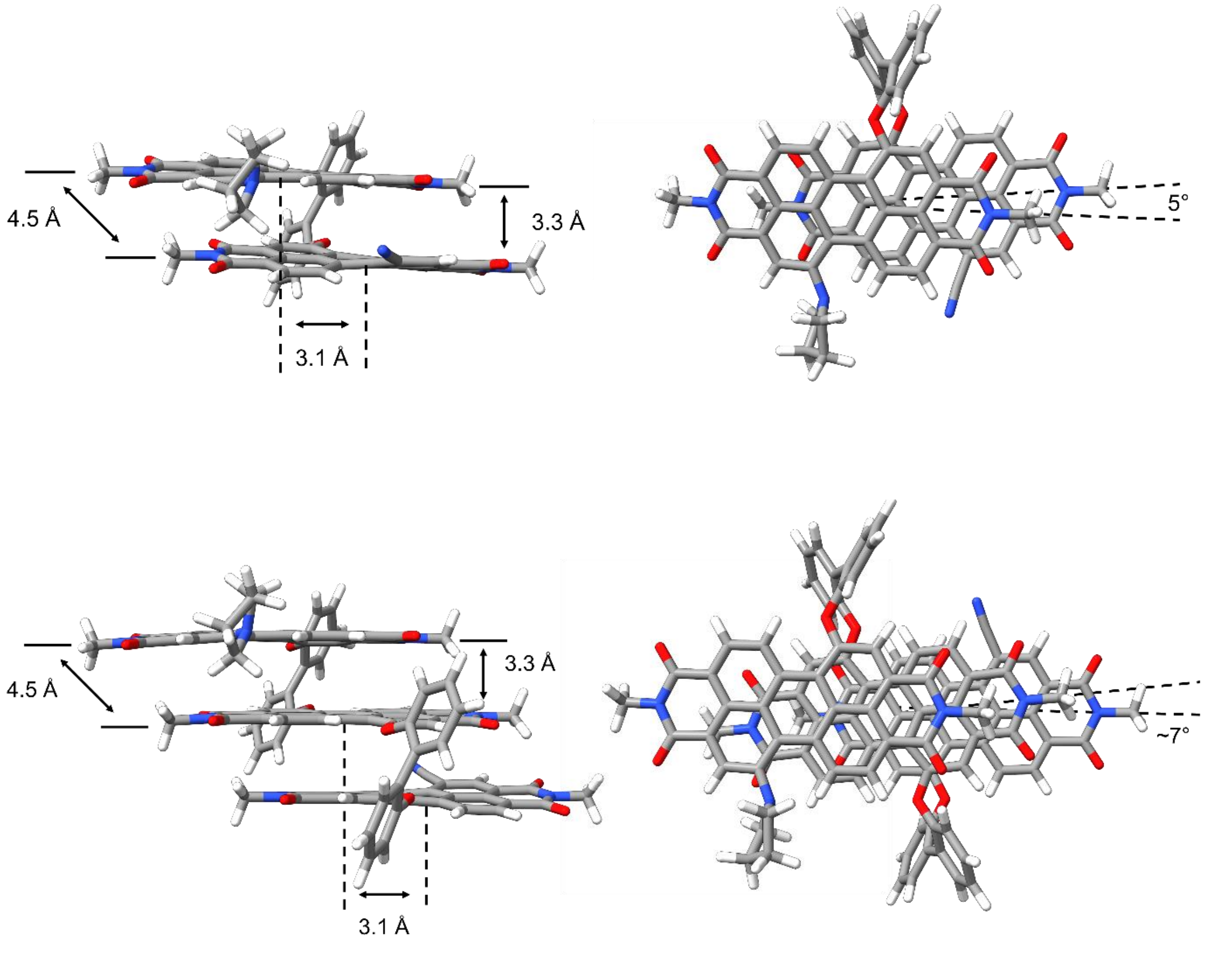

Supplementary Figure 7. Quantum chemical structure calculation. Front and top side view of the geometry-optimized structure of **DA-PBI2** (top), and **DA-PBI3** (bottom) (wB97X-D/def2-SVP) in THF (PCM). The solubilizing alkyl chains were replaced by methyl groups.

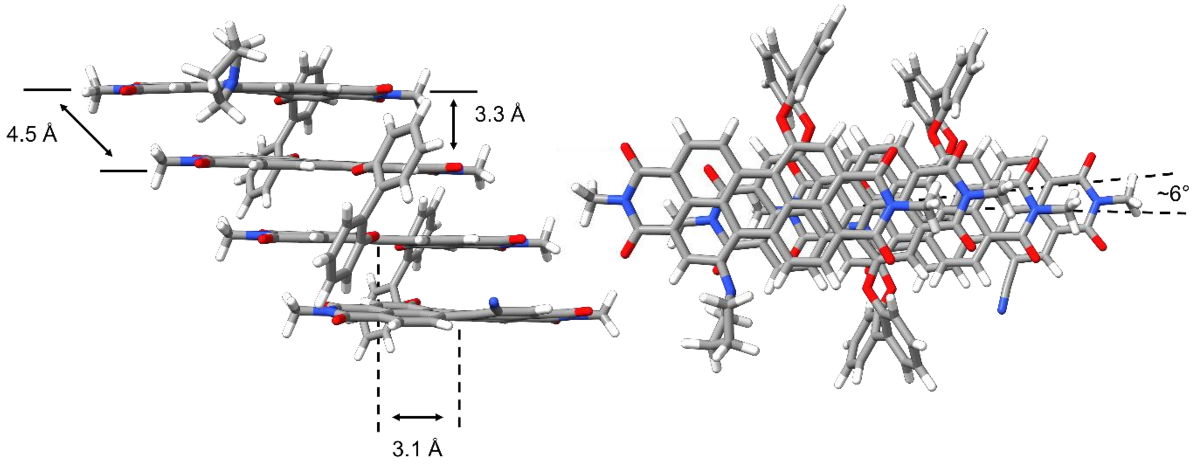

Supplementary Figure 8. Quantum chemical structure calculation. Front and top side view of the geometry-optimized structure of **DA-PBI4** (wB97X-D/def2-SVP) in THF (PCM). The solubilizing alkyl chains were replaced by methyl groups.

Supplementary Table 1. Quantum chemical structure calculation. Summary of the structural parameters of the DFT geometry optimization calculations for **DA-PBI2** to **DA-PBI4** in THF.

Parameter	π-π-Distance [Å]	Centroid Distance [Å]	Slip Angle [°]	Rotation [°]
**DA-PBI2**	3.3	4.5	48	5
**DA-PBI3**	3.3	9.0	47	7
**DA-PBI4**	3.3	13.5	48	6

The calculated stacked configurations align with our in-depth 2D NMR analysis. For instance, the nearly vertical orientation of the spacer moieties with respect to the PBI planes can be verified by intense cross-signals between *meta*-spacer protons and *ortho*/bay-protons of the perylene cores (Supplementary Fig. 1–3). In addition, the experimentally derived hydrodynamic radii (11.7, 13.7 and 15.1 Å) only slightly exceed those obtained from DFT calculations (9.43, 11.4 and 13.4 Å), which is in good accordance with the replacement of large alkyl chains by methyl groups for DFT calculations to reduce the computational cost (Supplementary Fig. 7, 8).

The quantum chemical calculations for determination of the different types of couplings (Supplementary Table 3) were conducted following the procedure of the previously reported **Bis-PBI2**[8] and **Tris-PBI**[2] molecules using the geometry optimized structures to perform time-dependent DFT (TD-DFT) calculations (Gaussian 16 program package, wB97X-D functional and def2-SVP basis set in Tol and THF (PCM)). Therefrom, also highest and lowest occupied natural transition orbitals (HONTO/LUNTO) have been derived using the Multiwfn software package.[13]

Initially the transition dipole moments μ_{eg} of the **D-PBI**, **PBI-1** and **A-PBI** reference monomers were calculated from the absorption spectra measured in Tol and THF at 295 K. It was determined as the integral of the reduced absorption band by[14]

$$\left|\mu_{\mathrm{eg}}\right|^2 = \frac{3hc\varepsilon_0 ln10}{2\pi^2 N_{\mathrm{A}}} \cdot \int_{\tilde{v}_1}^{\tilde{v}_2} \frac{\varepsilon(\tilde{v})}{\tilde{v}} d\tilde{v}. \quad (1)$$

The molar extinction coefficient is given by $\varepsilon(\tilde{v})$, the speed of light by c and the Planck constant by h. Furthermore, ε_0 represents the vacuum permittivity and N_A gives the Avogadro constant. Average values of μ_{eg} = 6.91 D, 7.18 D (**DA-PBI2**), 7.11 D, 7.35 D (**DA-PBI3**) and 7.21 D, 7.44 D (**DA-PBI4**) were obtained for their reference monomers in Tol and THF, respectively. Afterwards, these values were further applied to appropriately scale the transition charges determined by the Mulliken electron excitation analysis.

The long-range Coulomb coupling J_{Coul} was calculated applying TD-DFT calculations (wB97X-D/def2-SVP). The resulting transition density was projected onto atomic transition charges by a Mulliken style electron excitation analysis for the first excited state using the Multiwfn software package.[13] The Coulomb coupling J_{Coul} was calculated using the transition charge method.[15]

The exciton coupling energy was calculated according to supplementary eq. 2:

$$J_{\mathrm{Coul}} = \frac{1}{4\pi\varepsilon_0} \sum_{\mathrm{i}} \sum_{\mathrm{j}} \frac{q_{\mathrm{i}}^{(\mathrm{a})} \cdot q_{\mathrm{j}}^{(\mathrm{b})}}{\left|r_{\mathrm{i}}^{(\mathrm{a})} - r_{\mathrm{j}}^{(\mathrm{b})}\right|}. \quad (2)$$

The transition charge on atom *i* of chromophore *a* is defined by $q_i^{(a)}$. The position vector of the respective transition charge is given by $r_i^{(a)}$ and ε_0 gives the vacuum permittivity. As reported in literature, the transition dipole moments of the respective monomer layers were overestimated by the TD-DFT calculations ($\mu_{eg\,(TD\text{-}DFT)}$ = 9.27 D, 9.06 D (**DA-PBI2**), 9.46 D, 9.25 D (**DA-PBI3**), 9.61 D, 9.41 D (**DA-PBI4**)) in Tol and THF, respectively.[8,16] In comparison to the transition dipole values obtained for the integral of the reduced absorption band $\mu_{eg\,(TD\text{-}DFT)}$ is overestimated by a factor of 1.34, 1.33, 1.33 (Tol) and 1.26, 1.26, 1.26 (THF) for **DA-PBI2** to **DA-PBI4**, respectively. Therefore, the transition charges were scaled by a factor of 0.75, 0.75, 0.75 (Tol) and 0.80, 0.79, 0.79 (THF) for **DA-PBI2** to **DA-PBI4**, respectively.

The transfer integrals were determined within the unique fragment approach using the Amsterdam Density Functional (ADF) program[17,18] applying the PW91 functional[19] and a TZP basis set.[20] Given the overlap integrals S_e / S_h of the LUMO / HOMO, the electron / hole site energies $E_{e,1}$ / $E_{h,1}$ and $E_{e,2}$ / $E_{h,2}$ of the respective fragments 1 and 2, the effective electron (t_e) and hole (t_h) transfer integrals can be calculated by[8,21]

$$t_{\mathrm{e}} = \frac{t_{\mathrm{e}}' - \frac{1}{2}\left(E_{\mathrm{e},1} + E_{\mathrm{e},2}\right)S_{\mathrm{e}}}{1 - S_{\mathrm{e}}^2} \tag{3}$$

$$t_{\mathrm{h}} = \frac{t_{\mathrm{h}}' - \frac{1}{2}\left(E_{\mathrm{h},1} + E_{\mathrm{h},2}\right)S_{\mathrm{h}}}{1 - S_{\mathrm{h}}^2}. \tag{4}$$

The ADF calculations were performed by extracting the monomeric layers from **DA-PBI2** to **DA-PBI4** and replacing the spacer moieties by phenoxy units, without further geometry optimization. The solubilizing alkyl chains were replaced by methyl groups.

The short-range charge-transfer coupling J_{CT} was calculated at the perturbative limit[22,23] (charge-transfer state and local Frenkel exciton state should be well separated) with its effective electron (t_{e}) and hole (t_{h}) transfer integrals and the energy difference E_{CT}–E_{S1} = 3200 cm^{-1} for **DA-PBI2**, 4084 cm^{-1} for **DA-PBI3** and 4369 and 4332 cm^{-1} for **DA-PBI4** in Tol and THF, respectively (Supplementary Table 2 and 3). Energy values for the first excited virtual state (S_1) and the first virtual CT state have been derived from TD-DFT calculations (wB97X-D/def2-SVP) in Tol and THF (PCM) (Supplementary Table 4). Information of the CT character of the calculated excited states have been derived from qualitative interpretation of the NTO plots for **DA-PBI3** and **DA-PBI4** (Supplementary Fig. 10–12). Due to the pronounced CT character of the S_1 state for **DA-PBI2**, literature values of E_{CT}–E_{S1} = 3200 cm^{-1} had to be used. These values were in good accordance with those derived from monomeric PBI crystals[22] and reported to be suitable for the calculations of short-range coupling energies.[2,8,24]

The short-range charge-transfer coupling J_{CT} was calculated by[22,23]

$$J_{\mathrm{CT}} = -2\frac{t_{\mathrm{e}}t_{\mathrm{h}}}{E_{\mathrm{CT}} - E_{\mathrm{S_1}}}. \tag{5}$$

The resulting total coupling J_{Total} was determined as the sum of J_{Coul} and J_{CT}. The molecular structures obtained from the DFT geometry optimizations were used.

Supplementary Table 2. Quantum chemical calculation. Energy values of the calculated first excited LE (S_1) and CT states as well as their obtained energy gap in THF and Tol.

	THF			Tol		
	State	Energy [eV]	E_{CT}–E_{S1} [cm^{-1}]	State	Energy [eV]	E_{CT}–E_{S1} [cm^{-1}]
**DA-PBI2**	–	–	3200	–	–	3200
**DA-PBI3**	S_1	2.3000	4084	2.2964	2.2964	4084
	S_4	2.8063		2.8027	2.8027	
**DA-PBI4**	S_1	2.2593	4369	2.2581	2.2581	4332
	S_5	2.8010		2.7952	2.7952	

Supplementary Table 3. Quantum chemical coupling calculation. Calculated Coulomb, charge transfer coupling energies and hole/electron integrals for **DA-PBI2** to **DA-PBI4** in Tol and THF.

Couplings [cm^{-1}]	J_{Total} Tol | THF	J_{Coul}[a] Tol | THF	J_{CT}[b] Tol | THF	t_e Tol | THF	t_h Tol | THF
**DA-PBI2** (close)	97.0 | 125	419 | 445	–322 | –320	685 | 677	754 | 755
**DA-PBI3** (close)	49.0 | 12.0	445 | 475	–396 | –463	806 | 880	1003 | 1074
**DA-PBI3** (far, 3 units)	–84.4 | –90.3	–84.4 | –90.3	–	–	–
**DA-PBI4** (close)	6.00 | 19.0	458 | 484	–452 | –465	858 | 887	1141 | 1147
**DA-PBI4** (far, 3 units)	–86.1 | –91.9	–86.1 | –91.9	–	–	–
**DA-PBI4** (far, 4 units)	–7.08 | –7.19	–7.08 | –7.19	–	–	–

[a]Calculated by the transition charge method (averaged). [b]Determined based on supplementary eq. 5 (averaged).

The dependence of the effective electron/hole transfer integrals and the charge transfer coupling in THF has been investigated, as the longitudinal shift of the donor and acceptor chromophores of **DA-PBI2** (Supplementary Fig. 9) is increased from 0 to 11.4 Å at constant π-distances of 3.3 Å. The same calculation methods and values, as mentioned above, have been applied. The monomeric donor/acceptor chromophores were extracted from the geometry optimized structure of **DA-PBI2** in THF and were not further optimized.

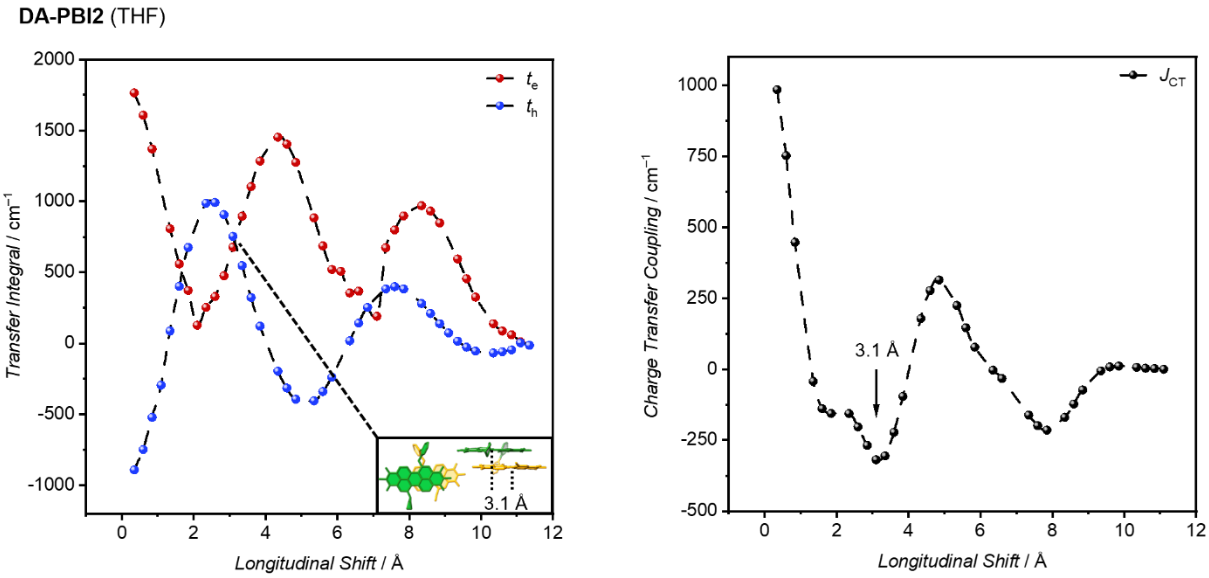

Supplementary Figure 9. Quantum chemical coupling calculation. Left: Effective electron and hole transfer integrals derived for **DA-PBI2** as a function of the longitudinal shift between the donor/acceptor chromophores in THF. Right: Charge transfer coupling values as a function of the longitudinal shift.

Supplementary Tabel 4 summarizes the excited state energies and oscillator strengths of the five lowest excited states obtained by TD-DFT calculations (wB97X-D/def2-SVP) for **DA-PBI2** to **DA-PBI4** in the gas phase, Tol and THF (PCM).

Supplementary Table 4. Quantum chemical calculation. Summary of the energy and oscillator strength of the lowest five calculated excited states applying TD-DFT calculations (wB97X-D/def2-SVP, PCM) in the gas phase, Tol and THF for **DA-PBI2** to **DA-PBI4**.

	**State**	**Gas phase**		**Tol**		**THF**	
		Energy [eV]	Oscillator Strength	Energy [eV]	Oscillator Strength	Energy [eV]	Oscillator Strength
**DA-PBI2**	S_1	2.4813	0.4121	2.4177	0.7251	2.4186	0.7032
	S_2	2.6064	0.1392	2.5457	0.1391	2.5335	0.1265
	S_3	2.9177	0.3487	2.8674	0.3867	2.8604	0.3253
	S_4	3.0403	0.1229	2.9687	0.1482	2.9344	0.1832
	S_5	3.4334	0.0668	3.4001	0.0641	3.3727	0.0624
**DA-PBI3**	S_1	2.3477	0.5439	2.2964	0.9787	2.3000	1.0109
	S_2	2.4699	0.1241	2.4114	0.1081	2.3971	0.0441
	S_3	2.5460	0.1686	2.5030	0.1647	2.4843	0.1454
	S_4	2.8459	0.3244	2.8027	0.3864	2.8063	0.3545
	S_5	2.9308	0.0058	2.9104	0.0157	2.8800	0.0334
**DA-PBI4**	S_1	2.3146	0.8198	2.2581	1.3057	2.2593	1.2653
	S_2	2.3934	0.0123	2.3518	0.0074	2.3536	0.0001
	S_3	2.4872	0.1898	2.4400	0.3042	2.4276	0.3201
	S_4	2.5317	0.1310	2.4917	0.0718	2.4796	0.0549
	S_5	2.8326	0.3329	2.7952	0.3946	2.8010	0.3402

Supplementary Tabel 5–7 summarize the calculated transitions for the first three lowest excited states with reasonable high oscillator strengths and their contribution. The transition to an electronic state is not predominated by one molecular orbital (MO) pair. Therefore, the natural transition orbital (NTO) method[25] has been chosen, to separately perform unitary transformations for occupied MOs and virtual MOs as implemented in the MultiWFN 3.8 programm package (Supplementary Fig. 10–12). To achieve acceptable accuracy, for the TD-DFT calculations (wB97X-D/def2-SVP) in Tol and THF (PCM) all configuration coefficients whose magnitude is larger than 0.001 have been taken into account.[13]

Supplementary Table 5. Quantum chemical calculation. Summary of the excited state transitions with their contributions of the lowest three calculated excited states, featuring reasonable high oscillator strengths, applying TD-DFT calculations (wB97X-D/def2-SVP, PCM) in the gas phase, Tol and THF for **DA-PBI2**.

**DA-PBI2**	**Gas phase**		**Tol**		**THF**	
	Transition	Contribution [%]	Transition	Contribution [%]	Transition	Contribution [%]
S_1	H–1 → L	6	H–1 → L	7	H–1 → L	7
	H → L	89	H → L	87	H → L	87
S_2	H–1 → L	21	H–1 → L	22	H–1 → L	23
	H → L	3	–	–	–	–
	H → L+1	70	H → L+1	72	H → L+1	71
S_3	H–2 → L	4	H–2 → L	4	H–2 → L	7
	H–1 → L	64	H–1 → L	60	H–1 → L	57
	H–1 → L+1	3	H–1 → L+1	3	H–1 → L+1	4
	H → L	3	H → L	8	H → L	8
	H → L+1	20	H → L+1	19	H → L+1	18

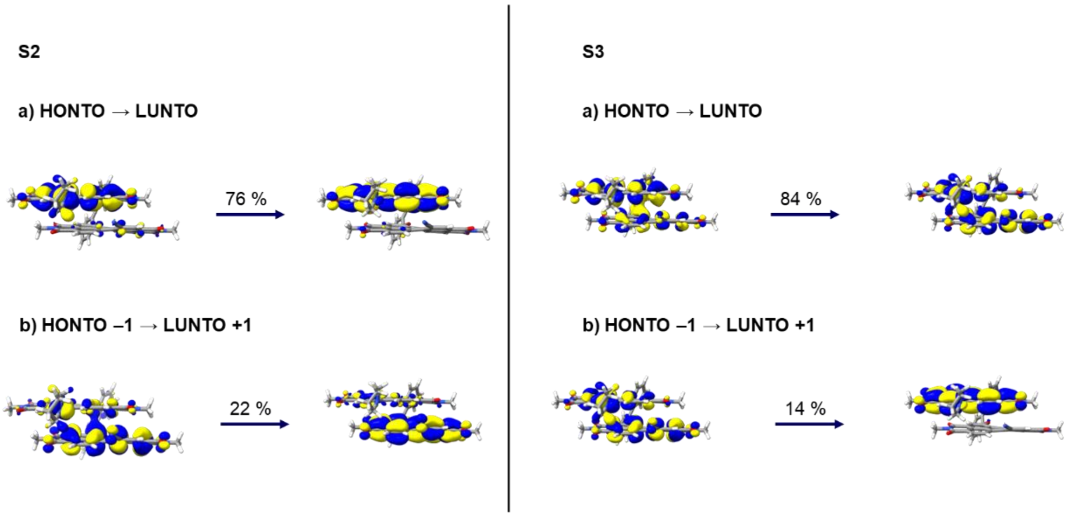

Supplementary Figure 10. Natural transition orbitals. a) HONTO and LUNTO and **b)** HONTO–1 and LUNTO+1 orbital plots for the important transitions to the S2 (left) and S3 (right) state of the **DA-PBI2** array with their contributions (%) in THF (isovalue 0.02) derived from TD-DFT calculations.

Supplementary Table 6. Quantum chemical calculation. Summary of the excited state transitions with their contributions of the lowest three calculated excited states, featuring reasonable high oscillator strengths, applying TD-DFT calculations (wB97X-D/def2-SVP, PCM) in the gas phase, Tol and THF for **DA-PBI3**.

**DA-PBI3**	**Gas phase**		**Tol**		**THF**	
	Transition	Contribution [%]	Transition	Contribution [%]	Transition	Contribution [%]
S_1	H–1 → L	17	H–1 → L	15	H–1 → L	13
	H → L	74	H → L	69	H → L	66
	H → L+1	3	H → L+1	9	H → L+1	14
S_3	H–2 → L	9	H–2 → L	15	H–2 → L	16
	H–1 → L+1	32	H–1 → L+1	31	H–1 → L+1	31
	–	–	–	–	H → L+1	2
	H → L+2	73	H → L+2	49	H → L+2	46
S_4	H–2 → L	59	H–2 → L	55	H–2 → L	53
	H–2 → L+1	7	H–2 → L+1	9	H–2 → L+1	9
	H–1 → L	6	H–1 → L	3	H–1 → L	3
	H–1 → L+1	5	H–1 → L+1	4	H–1 → L+1	5
	H → L	4	H → L	7	H → L	7
	H → L+1	12	H → L+1	13	H → L+1	14
	H → L+2	2	H → L+2	3	H → L+2	2

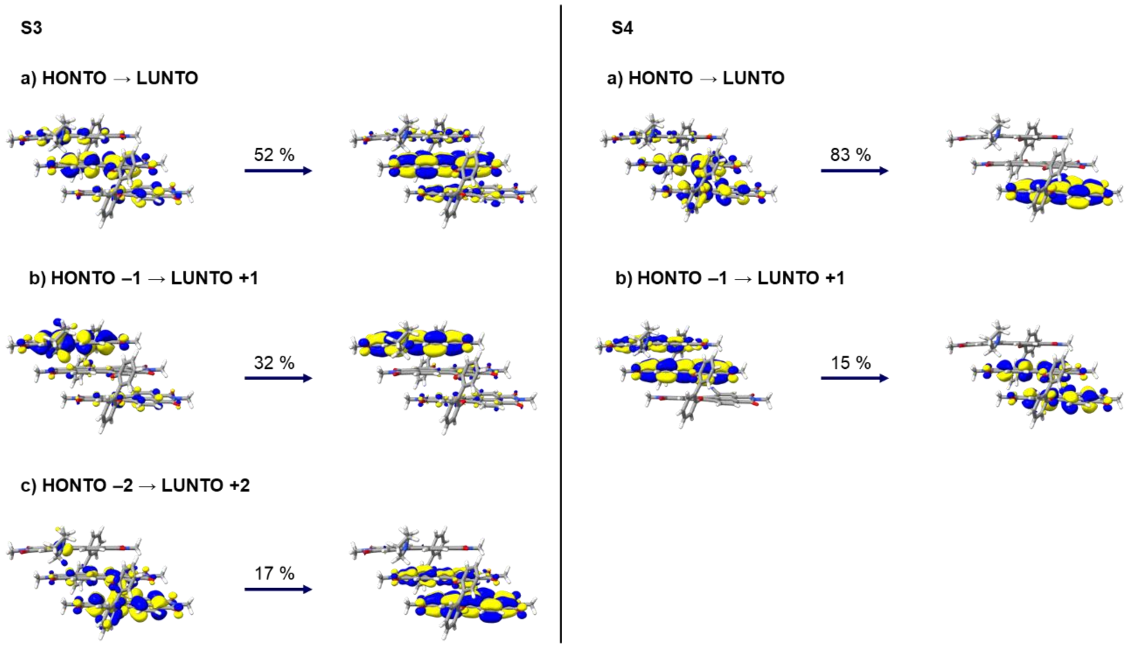

Supplementary Figure 11. Natural transition orbitals. a) HONTO and LUNTO and **b)** HONTO–1 and LUNTO+1 orbital plots for the important transitions to the S3 (left) and S4 (right) state of the **DA-PBI3** array with their contributions (%) in THF (isovalue 0.02) derived from TD-DFT calculations.

Supplementary Table 7. Quantum chemical calculation. Summary of the excited state transitions with their contributions of the lowest three calculated excited states, featuring reasonable high oscillator strengths, applying TD-DFT calculations (wB97X-D/def2-SVP, PCM) in the gas phase, Tol and THF for **DA-PBI4**.

**DA-PBI4**	**Gas phase**		**Tol**		**THF**	
	Transition	Contribution [%]	Transition	Contribution [%]	Transition	Contribution [%]
	H –2 → L+1	2	H –2 → L+1	2	H –2 → L+1	2
	H –1 → L	15	H –1 → L	13	H –1 → L	11
S_1	H → L	52	–	–	H–1 → L+2	2
	H → L+1	23	H → L	49	H → L	48
	H → L+2	2	H → L+1	28	H → L+1	31
	H–3 → L	9	H–3 → L	6	H–3 → L	4
	H–2 → L	3	H–2 → L	5	H–2 → L	8
	–	–	H–2 → L+2	3	H–2 → L+2	3
S_3	H–1 → L+1	30	H–1 → L+1	29	H–1 → L+1	27
	–	–	–	–	H–1 → L+2	3
	H–1 → L+3	5	H–1 → L+3	7	H–1 → L+3	7
	H → L+2	42	H → L+2	42	H → L+2	35
	H → L+3	2	–	–	H → L+3	6
	H–3 → L	43	H–3 → L	43	H–3 → L	32
	H–3 → L+1	11	H–3 → L+1	12	H–3 → L+1	14
	H–2 → L	10	H–2 → L	9	H–2 → L	10
S_5	H–1 → L+1	12	H–1 → L+1	10	H–1 → L+1	15
	–	–	H–1 → L+2	2	–	–
	H → L	5	H → L	7	H → L	8
	H → L+1	7	H → L+1	8	H → L+1	8

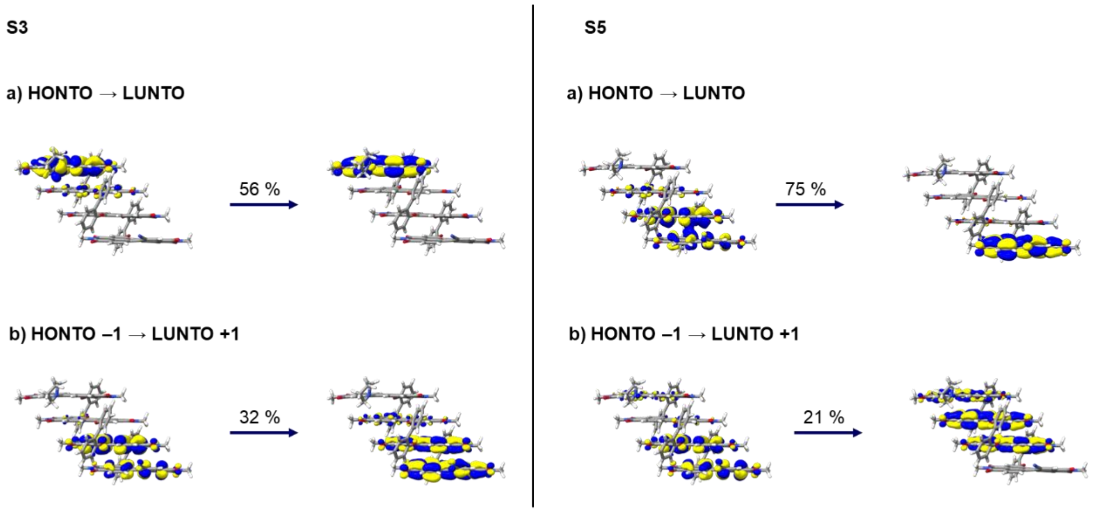

Supplementary Figure 12. Natural transition orbitals. a) HONTO and LUNTO and **b)** HONTO–1 and LUNTO+1 orbital plots for the important transitions to the S3 (left) and S5 (right) state of the **DA-PBI4** array with their contributions (%) in THF (isovalue 0.02) derived from TD-DFT calculations.

The supplemented Weller equation (supplementary eq 6 and 7) have been applied to obtain the Gibbs free energies ΔG_{CS} and ΔG_{CR}.[26–29]

$$\Delta G_{\mathrm{CS}} = e\left[E_{\mathrm{Ox(D)}} - E_{\mathrm{Red(A)}}\right] - E_{00} - \frac{1}{4\pi\varepsilon_0\varepsilon_{\mathrm{B}}}\sum_i\sum_j \frac{q_{\mathrm{i}}^{(\mathrm{a})}\cdot q_{\mathrm{j}}^{(\mathrm{b})}}{\left|r_{\mathrm{i}}^{(\mathrm{a})} - r_{\mathrm{j}}^{(\mathrm{b})}\right|} - \frac{e^2}{8\pi\varepsilon_0}\left(\frac{1}{r_{\mathrm{D}}} + \frac{1}{r_{\mathrm{A}}}\right)\left(\frac{1}{\varepsilon_{\mathrm{CV}}} - \frac{1}{\varepsilon_{\mathrm{S}}}\right) \quad (6)$$

$$\Delta G_{\mathrm{CR}} = -(\Delta G_{\mathrm{CS}} + E_{00}) \quad (7)$$

As described in the main text, the original Weller equation[30] has been supplemented to address distributed point charges instead of hard sphere point charges. The screening factor of the surrounding solvent and bridging units within the π-stack (ε_B) has been kept to the dielectric constant of dichloromethane (8.93) to not include additional uncertainties. The free energy values are summarized in Supplementary Table 8.

Supplementary Tabel 8. Weller calculations. Calculated Gibbs free energy for the intramolecular charge separation and recombination processes in DCM.

DCM	E_{ox} [V]	E_{Red} [V]	E_{00} [eV]	ε_B	ΔG_{redox} [eV]	ΔG_{Coul} [eV]	ΔG_{CS} [eV]	ΔG_{CR} [eV]
**DA-PBI2** (D^+ to A^-)	0.43	–1.07	1.80	8.93	–0.30	–0.23	–0.53	–1.27
**DA-PBI3** (D^+ to Bridge$^-$)	0.43	–1.13	1.80	8.93	–0.24	–0.24	–0.48	–1.32
**DA-PBI3** (D^+ to A^-)	0.43	–1.07	1.80	8.93	–0.30	–0.15	–0.45	–1.35
**DA-PBI4** (D^+ to Bridge$_1^-$)	0.43	–1.13	1.80	8.93	–0.24	–0.24	–0.48	–1.32
**DA-PBI4** (D^+ to Bridge$_2^-$)	0.43	–1.13	1.80	8.93	–0.24	–0.16	–0.46	–1.34
**DA-PBI4** (D^+ to A^-)	0.43	–1.07	1.80	8.93	–0.30	–0.11	–0.41	–1.39

5. UV/Vis Absorption and Fluorescence Spectroscopy

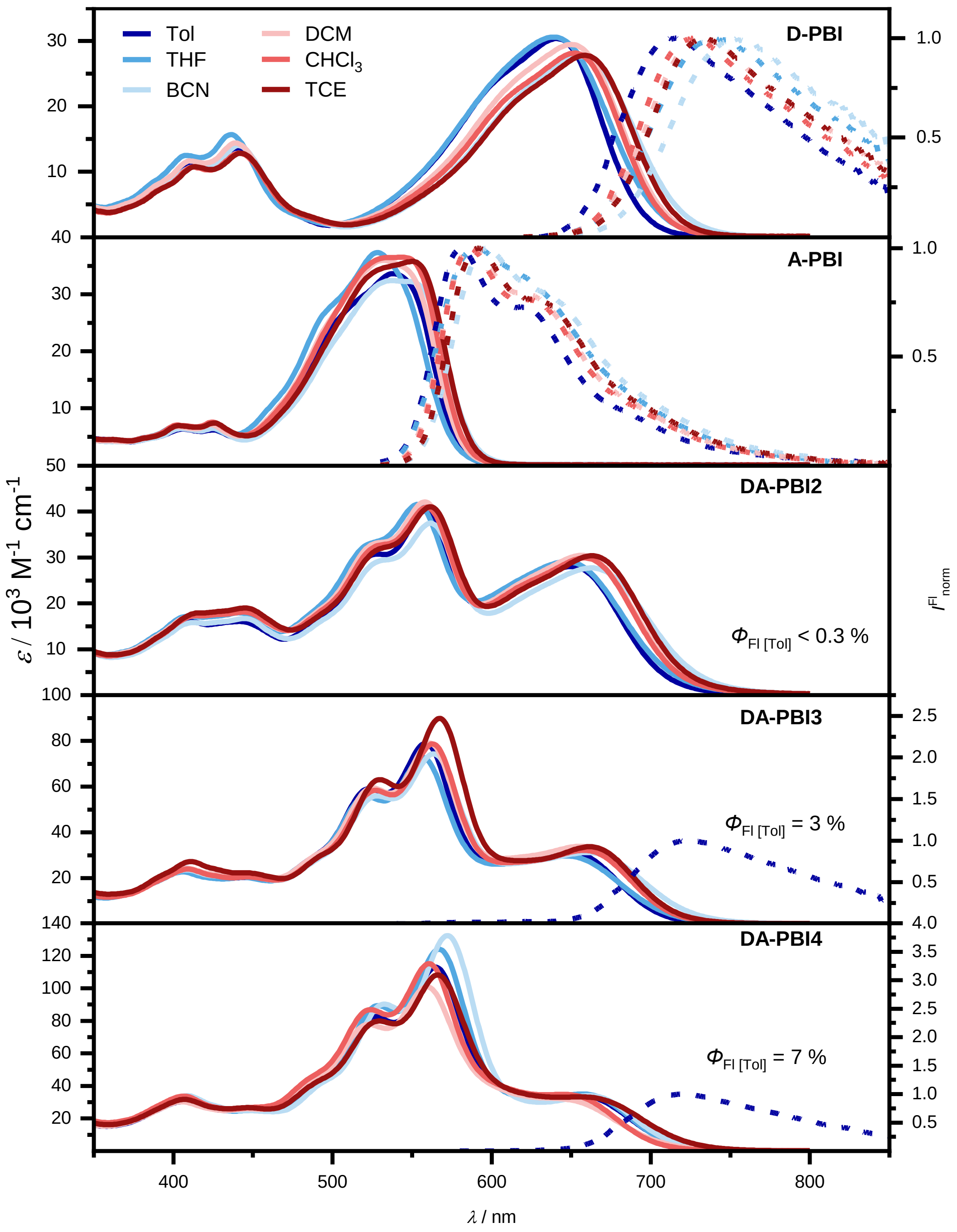

Supplementary Figure 13. Steady-state spectroscopic characterization. Absorption (solid line) and emission (dashed line) spectra of **D-PBI**, **A-PBI**, **DA-PBI2**, **DA-PBI3** and **DA-PBI4** in Tol (dark blue), THF (blue), BCN (light blue), DCM (light red), $CHCl_3$ (red), TCE (dark red). Absorption measurements c_0 ~ 1.0×10^{-5} M. Emission measurements OD < 0.05, Fluorescence quantum yields: Absolute values c_0 ~ 1.0×10^{-5} M, with reabsorption correction.

Supplementary Table 9. Optical characterization. Spectroscopic data obtained from UV/Vis and fluorescence measurements of **D-PBI** to **DA-PBI4** in toluene at 298 K.

Toluene	D-PBI	PBI-1	A-PBI	DA-PBI2	DA-PBI3	DA-PBI4
$\lambda_{abs}(A_{0-0})^a$ / nm	646	537	555	558	559	561
$\lambda_{abs}(A_{0-1})^a$ / nm	596	503	536	528	523	524
$\varepsilon_{max}(A_{0-0})^a$ / M^{-1} cm^{-1}	30 370	48 860	27 919	40 223	78 970	115 170
$\varepsilon_{max}(A_{0-1})^a$ / M^{-1} cm^{-1}	22 225	32 740	33 535	30 733	59 130	86 842
$[A_{0-0} / A_{0-1}]^a$	1.37	1.49	0.83	–	–	–
$\lambda_{em}{}^b$ / nm	716	570	581	–	722	719
$\Phi_{Fl}{}^c$ / %	21^c	95^c	95^c	<0.3^c	3^c	7^c
$\tau_{Fl,1}{}^b$ / ns	2.91	4.45	5.98	–	1.35 (65 %)	2.21 (100 %)
$\tau_{Fl,2}{}^b$ / ns	–	–	–	–	3.10 (35 %)	–

^{a}UV/Vis $c_0 \sim 1.0\times10^{-5}$ M. ^{b}Fluorescence under high diluted conditions $OD < 0.05$, time-resolved measurements with ps laser diode with excitation wavelength of 505 nm. ^{c}Fluorescence quantum yields: absolute values $c_0 \sim 1.0\times10^{-5}$ M, with reabsorption correction.

Supplementary Table 10. Optical characterization. Spectroscopic data obtained from UV/Vis and fluorescence measurements of **D-PBI** to **DA-PBI4** in THF at 298 K.

THF	D-PBI	PBI-1	A-PBI	DA-PBI2	DA-PBI3	DA-PBI4
$\lambda_{abs}(A_{0-0})^a$ / nm	649	545	544	553	557	559
$\lambda_{abs}(A_{0-1})^a$ / nm	599	511	525	516	522	523
$\varepsilon_{max}(A_{0-0})^a$ / M^{-1} cm^{-1}	30 590	47 310	32 180	41 561	72 750	100 967
$\varepsilon_{max}(A_{0-1})^a$ / M^{-1} cm^{-1}	23 284	32 680	36 992	31 107	55 718	77 841
$[A_{0-0} / A_{0-1}]^a$	1.31	1.45	0.87	–	–	–
$\lambda_{em}{}^b$ / nm	739	567	589	–	–	–
$\Phi_{Fl}{}^c$ / %	10^c	94^c	88^c	–	–	–
$\tau_{Fl,1}{}^b$ / ns	1.82	4.64	6.14	–	–	–
$\tau_{Fl,2}{}^b$ / ns	–	–	–	–	–	–

^{a}UV/Vis $c_0 \sim 1.0\times10^{-5}$ M. ^{b}Fluorescence under high diluted conditions $OD < 0.05$, time-resolved measurements with ps laser diode with excitation wavelength of 505 nm. ^{c}Fluorescence quantum yields: absolute values $c_0 \sim 1.0\times10^{-5}$ M, with reabsorption correction.

Supplementary Table 11. Optical characterization. Spectroscopic data obtained from UV/Vis and fluorescence measurements of **D-PBI** to **DA-PBI4** in BCN at 298 K.

BCN	D-PBI	PBI-1	A-PBI	DA-PBI2	DA-PBI3	DA-PBI4
$\lambda_{abs}(A_{0-0})^a$ / nm	664	545	557	562	563	566
$\lambda_{abs}(A_{0-1})^a$ / nm	611	511	529	527	528	529
$\varepsilon_{max}(A_{0-0})^a$ / M^{-1} cm^{-1}	27 637	47 306	30 945	37 493	74 320	108 235
$\varepsilon_{max}(A_{0-1})^a$ / M^{-1} cm^{-1}	20 071	32 679	31 730	28 920	55 920	79 890
$[A_{0-0} / A_{0-1}]^a$	1.38	1.45	0.98	–	–	–
$\lambda_{em}{}^b$ / nm	754	579.	596	–	–	–
$\Phi_{Fl}{}^c$ / %	12[c]	94[c]	86[c]	–	–	–
$\tau_{Fl,1}{}^b$ / ns	1.85	4.63	5.89	–	–	–
$\tau_{Fl,2}{}^b$ / ns	–	–	–	–	–	–

[a]UV/Vis $c_0 \sim 1.0\times10^{-5}$ M. [b]Fluorescence under high diluted conditions *OD* < 0.05, time-resolved measurements with ps laser diode with excitation wavelength of 505 nm. [c]Fluorescence quantum yields: absolute values $c_0 \sim 1.0\times10^{-5}$ M, with reabsorption correction.

Supplementary Table 12. Optical characterization. Spectroscopic data obtained from UV/Vis and fluorescence measurements of **D-PBI** to **DA-PBI4** in DCM at 298 K.

DCM	D-PBI	PBI-1	A-PBI	DA-PBI2	DA-PBI3	DA-PBI4
$\lambda_{abs}(A_{0-0})^a$ / nm	650	539	557	558	561	565
$\lambda_{abs}(A_{0-1})^a$ / nm	606	505	533	520	525	527
$\varepsilon_{max}(A_{0-0})^a$ / M^{-1} cm^{-1}	29 458	48 392	28 791	42 127	78 982	112 976
$\varepsilon_{max}(A_{0-1})^a$ / M^{-1} cm^{-1}	21 860	33 080	35 934	31 502	59 483	82 673
$[A_{0-0} / A_{0-1}]^a$	1.35	1.46	0.80	–	–	–
$\lambda_{em}{}^b$ / nm	734	572	587	–	–	–
$\Phi_{Fl}{}^c$ / %	16[c]	95[c]	87[c]	–	–	<0.2[c]
$\tau_{Fl,1}{}^b$ / ns	2.46	4.66	6.11	–	–	–
$\tau_{Fl,2}{}^b$ / ns	–	–	–	–	–	–

[a]UV/Vis $c_0 \sim 1.0\times10^{-5}$ M. [b]Fluorescence under high diluted conditions *OD* < 0.05, time-resolved measurements with ps laser diode with excitation wavelength of 505 nm. [c]Fluorescence quantum yields: absolute values $c_0 \sim 1.0\times10^{-5}$ M, with reabsorption correction.

Supplementary Table 13. Optical characterization. Spectroscopic data obtained from UV/Vis and fluorescence measurements of **D-PBI** to **DA-PBI4** in CHCl3 at 298 K.

$CHCl_3$	D-PBI	PBI-1	A-PBI	DA-PBI2	DA-PBI3	DA-PBI4
$\lambda_{abs}(A_{0–0})^a$ / nm	652	542	557	559	563	567
$\lambda_{abs}(A_{0–1})^a$ / nm	608	508	529	519	527	529
$\varepsilon_{max}(A_{0–0})^a$ / M^{-1} cm^{-1}	28 142	50 975	32 824	40 972	78 656	124 046
$\varepsilon_{max}(A_{0–1})^a$ / M^{-1} cm^{-1}	20 887	34 601	36 177	29 959	58 366	89 378
$[A_{0–0} / A_{0–1}]^a$	1.35	1.47	0.91	–	–	–
$\lambda_{em}{}^b$ / nm	725	575	587	–	–	–
$\Phi_{Fl}{}^c$ / %	17^c	95^c	88^c	–	$<0.1^c$	$<0.5^c$
$\tau_{Fl,1}{}^b$ / ns	2.65	4.56	6.02	–	–	–
$\tau_{Fl,2}{}^b$ / ns	–	–	–	–	–	–

[a]UV/Vis c_0 ~ 1.0×10^{-5} M. [b]Fluorescence under high diluted conditions *OD* < 0.05, time-resolved measurements with ps laser diode with excitation wavelength of 505 nm. [c]Fluorescence quantum yields: absolute values c_0 ~ 1.0×10^{-5} M, with reabsorption correction.

Supplementary Table 14. Optical characterization. Spectroscopic data obtained from UV/Vis and fluorescence measurements of **D-PBI** to **DA-PBI4** in TCE at 298 K.

TCE	D-PBI	PBI-1	A-PBI	DA-PBI2	DA-PBI3	DA-PBI4
$\lambda_{abs}(A_{0–0})^a$ / nm	658	546	560	561	567	572
$\lambda_{abs}(A_{0–1})^a$ / nm	614	511	529	519	530	533
$\varepsilon_{max}(A_{0–0})^a$ / M^{-1} cm^{-1}	27 798	51 253	37 340	41 063	89 833	132 400
$\varepsilon_{max}(A_{0–1})^a$ / M^{-1} cm^{-1}	20 515	34 548	32 702	28 612	63 047	90 274
$[A_{0–0} / A_{0–1}]^a$	1.36	1.48	1.14	–	–	–
$\lambda_{em}{}^b$ / nm	733	577	592	–	–	–
$\Phi_{Fl}{}^c$ / %	17^c	94^c	88^c	–	–	$<0.2^c$
$\tau_{Fl,1}{}^b$ / ns	2.47	4.53	5.82	–	–	–
$\tau_{Fl,2}{}^b$ / ns	–	–	–	–	–	–

[a]UV/Vis c_0 ~ 1.0×10^{-5} M. [b]Fluorescence under high diluted conditions *OD* < 0.05, time-resolved measurements with ps laser diode with excitation wavelength of 505 nm. [c]Fluorescence quantum yields: absolute values c_0 ~ 1.0×10^{-5} M, with reabsorption correction.

6. Cyclic Voltammetry

The first reduction potential exhibits an ascending trend from the donor **D-PBI** (–1.24 V), over the bridge **PBI-1** (–1.13 V), towards the acceptor **A-PBI** (–1.07 V), accompanied by a corresponding increase in oxidation potentials. As anticipated, **A-PBI** does not display any oxidation process within the electrochemical window of DCM, while **D-PBI** exhibits two distinct oxidation waves.

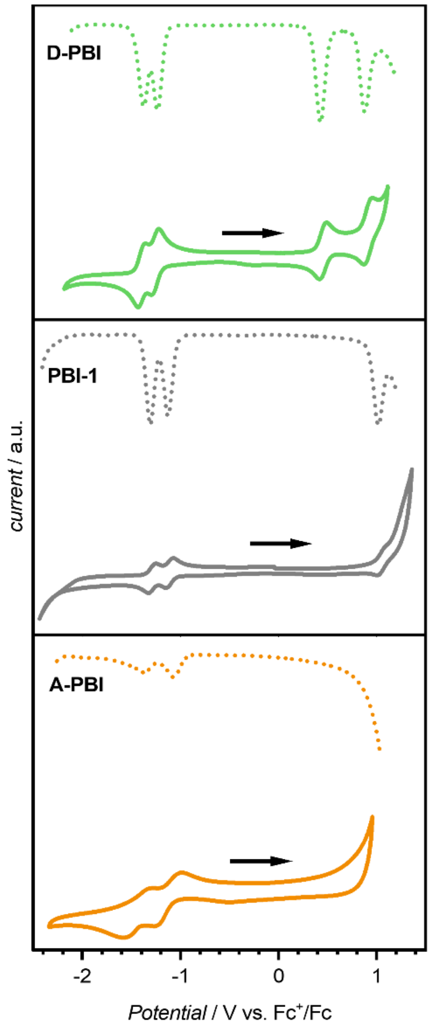

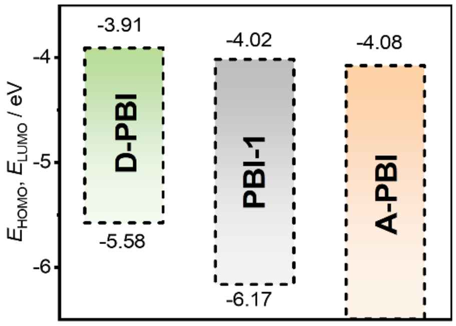

Electrochemical half-wave redox potentials of the reference compounds.

	D-PBI	PBI-1	A-PBI
E_{red1} [V]	−1.24	−1.13	−1.07
E_{red2} [V]	−1.38	−1.31	−1.36
E_{ox1} [V]	0.43	1.02	–
E_{ox2} [V]	0.88	–	–
E_{LUMO} [eV]	–3.91	–4.02	–4.08
E_{HOMO} [eV]	–5.58	–6.17	–

Supplementary Figure 14. Cyclic voltammetry. Left: Differential pulse (dotted line) and cyclic (solid line) voltammograms of **D-PBI** (top, green), **PBI-1** (middle, gray) and **A-PBI** (bottom, orange) ($c_0 \approx 1.0\times10^{-3}$ M, 0.1 M (n-Bu)$_4$NPF$_6$ (TBAHFP) in dry, degassed DCM) under argon atmosphere and a scan speed of 100 mVs^{-1} at 298 K (the direction of the scan is indicated by arrows). All potentials were calculated by differential pulse voltammetry referenced against the Fc$^{0/+}$ redox couple. Right: HOMO and LUMO energy levels of **D-PBI** (green), **PBI-1** (gray) and **A-PBI** (orange) calculated using the equations $E_{LUMO} = -[E(M/M^-) + 5.15\text{ eV}]$ and $E_{HOMO} = -[E(M/M^+) + 5.15\text{ eV}]$, assuming that the energy level of Fc$^+$/Fc with respect to the vacuum level is −5.15 eV.

7. Spectroelectrochemistry

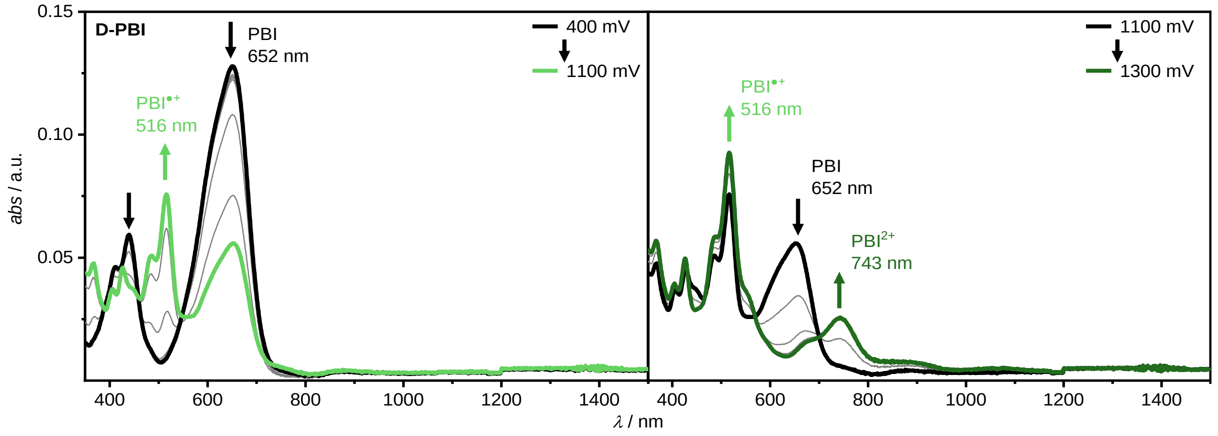

Supplementary Figure 15. Spectroelectrochemistry. UV/Vis/NIR absorption spectra of the neutral (black), radical cation (light green) and dicationic (dark green) PBI species of **D-PBI** measured in dry, degassed DCM ($c_0 \approx 4.0 \times 10^{-4}$ M) at room temperature using TBAHFP (0.1 M) as electrolyte.[31] Arrows indicate increasing or decreasing signal intensity.

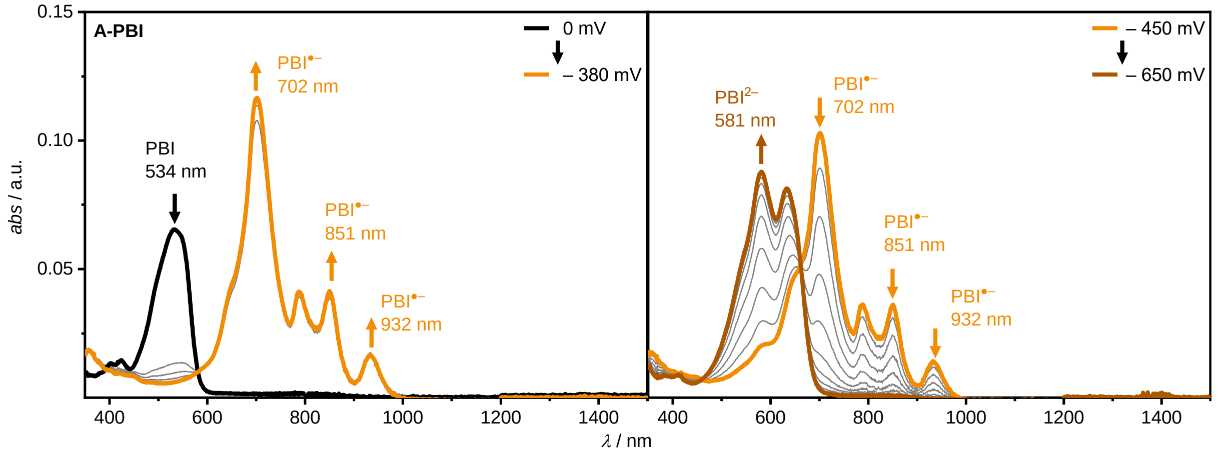

Supplementary Figure 16. Spectroelectrochemistry. UV/Vis/NIR absorption spectra of the neutral (black), radical anion (orange) and dianionic (brown) PBI species of **A-PBI** measured in dry, degassed DCM ($c_0 \approx 4.0 \times 10^{-4}$ M) at room temperature using TBAHFP (0.1 M) as electrolyte.[31] Arrows indicate increasing or decreasing signal intensity.

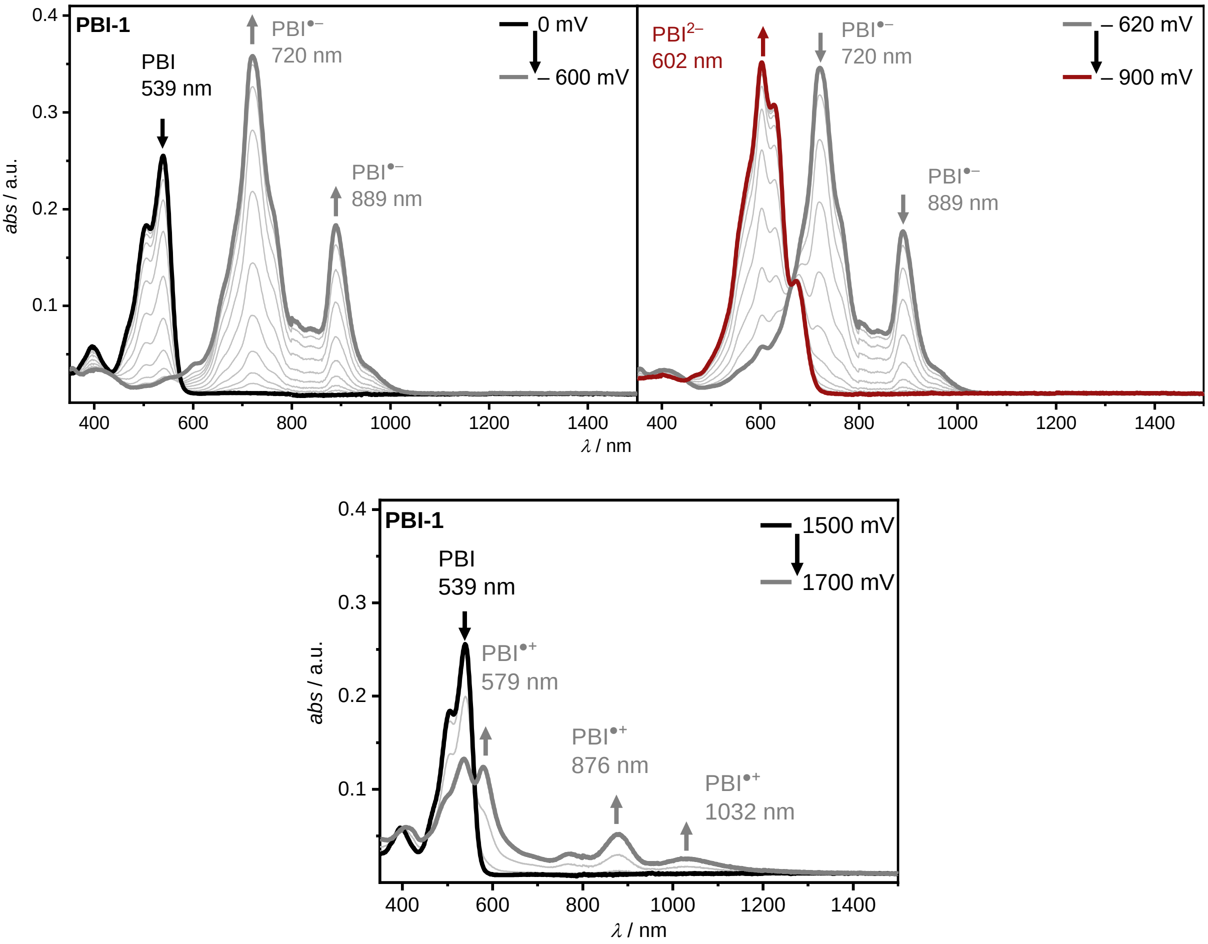

Supplementary Figure 17. Spectroelectrochemistry. UV/Vis/NIR absorption spectra of the neutral (black), radical anion (gray, top), radical cation (gray, bottom) and dianionic (dark red) PBI species of **PBI-1** measured in dry, degassed DCM ($c_0 \approx 4.0 \times 10^{-4}$ M) at room temperature using TBAHFP (0.1 M) as electrolyte.[31] Arrows indicate increasing or decreasing signal intensity.

8. Time-Resolved Spectroscopy

The TA spectra of **D-PBI**, excited at 600 nm in Tol, exhibit a prominent ground-state bleach (GSB) at 610 nm, stimulated emission (SE) at 720 nm, and excited-state absorption (ESA) at 500 nm (Supplementary Fig. 18a). Global fitting analysis (Supplementary Fig. 19) discloses two sequential relaxation processes for the excited state, a 5.0 ps structural relaxation followed by a 2.5 ns decay back to the ground state. In contrast, THF induces a notable red shift within the initial picoseconds and an accelerated decay of the SE at 710 nm (Supplementary Fig. 18c). The red shift, occurring within 2.4 ps, is ascribed to an augmented dipole moment, succeeded by the decay of the stabilized state back to the ground state within 1.5 ns. Additionally, TA spectra in THF at approximately 100 ps reveal the emergence of a new ESA band at around 690 nm, associated with the absorption of the **D-PBI** radical anion (Fig. 2d). The discerned *intra*molecular CT character for **D-PBI** is substantiated by the appearance of the **D-PBI** radical anion absorption, and a reduction in fluorescence quantum yield and lifetime with increasing solvent polarity. Conversely, transient absorption measurements (Supplementary Fig. 20) and global fitting analysis of **A-PBI** in Tol and THF (Supplementary Fig. 21) with two similar time constants (2 ps and 4 ns) suggest that **A-PBI** itself does not undergo any *intra*molecular CT processes, supported by the constant fluorescence quantum yield and lifetime in solvents with different polarity (Supplementary Table 9–14).

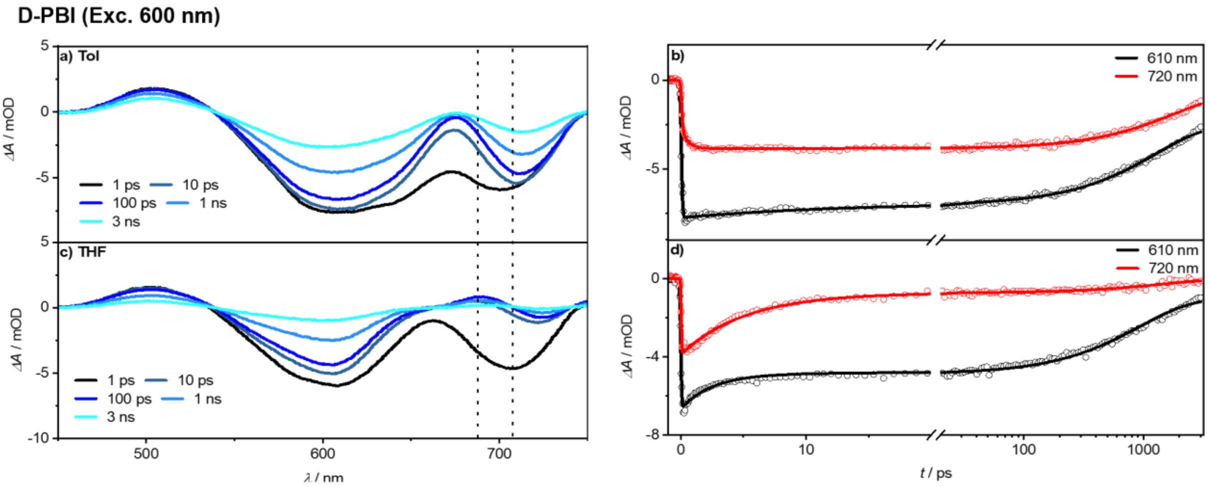

Supplementary Figure 18. Time-resolved characterization. Transient absorption spectra of **D-PBI** in Tol **a)** and THF **c)** at selected delay times with 600 nm excitation. Transient absorption kinetic traces in Tol **b)** and THF **d)** probed at 610 and 720 nm and the corresponding single-wavelength fitting line.

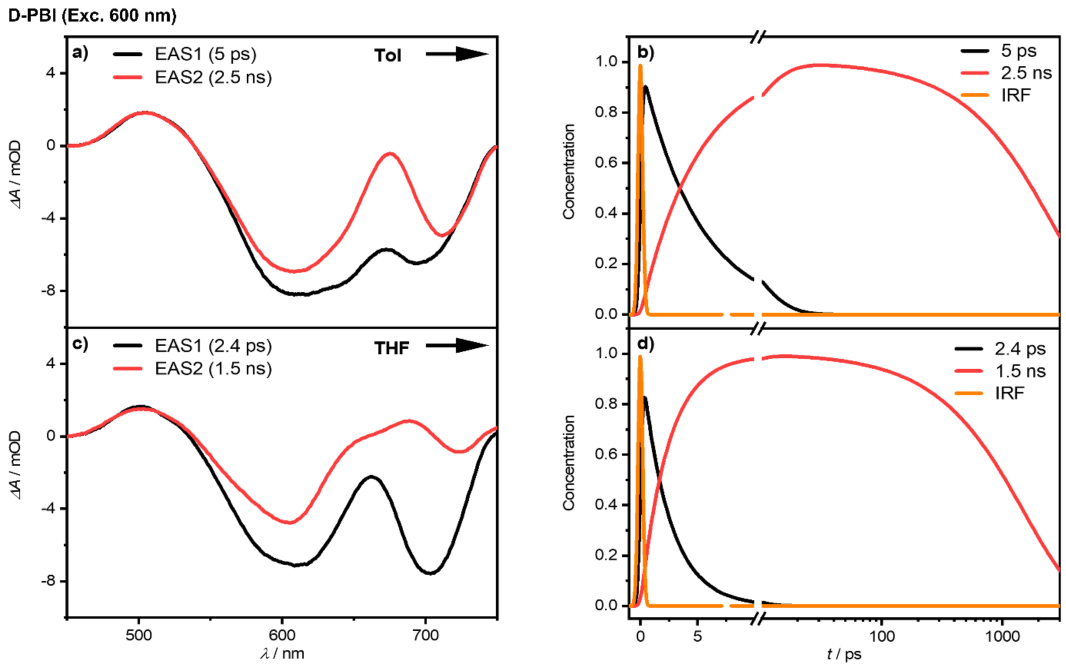

Supplementary Figure 19. Time-resolved characterization. Evolution-associated spectra **a), c)** and the corresponding population profiles **b), d)** of **D-PBI** in Tol **a), b)** and THF **c), d)** obtained by global fitting analysis with 600 nm excitation.

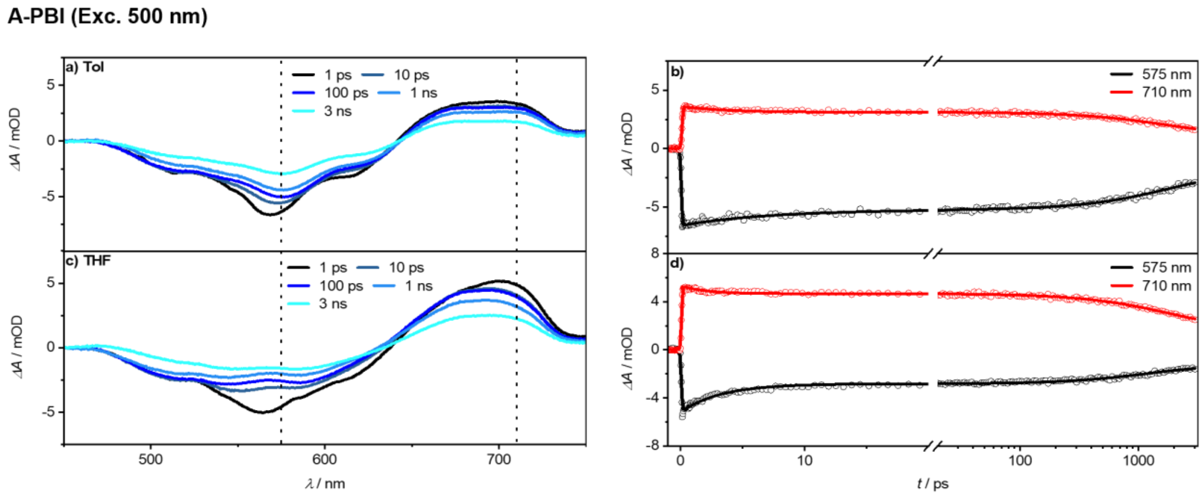

Supplementary Figure 20. Time-resolved characterization. Transient absorption spectra of **A-PBI** in Tol **a)** and THF **c)** at selected delay times with 500 nm excitation. Transient absorption kinetic traces in Tol **b)** and THF **d)** probed at 575 and 710 nm and the corresponding single-wavelength fitting line.

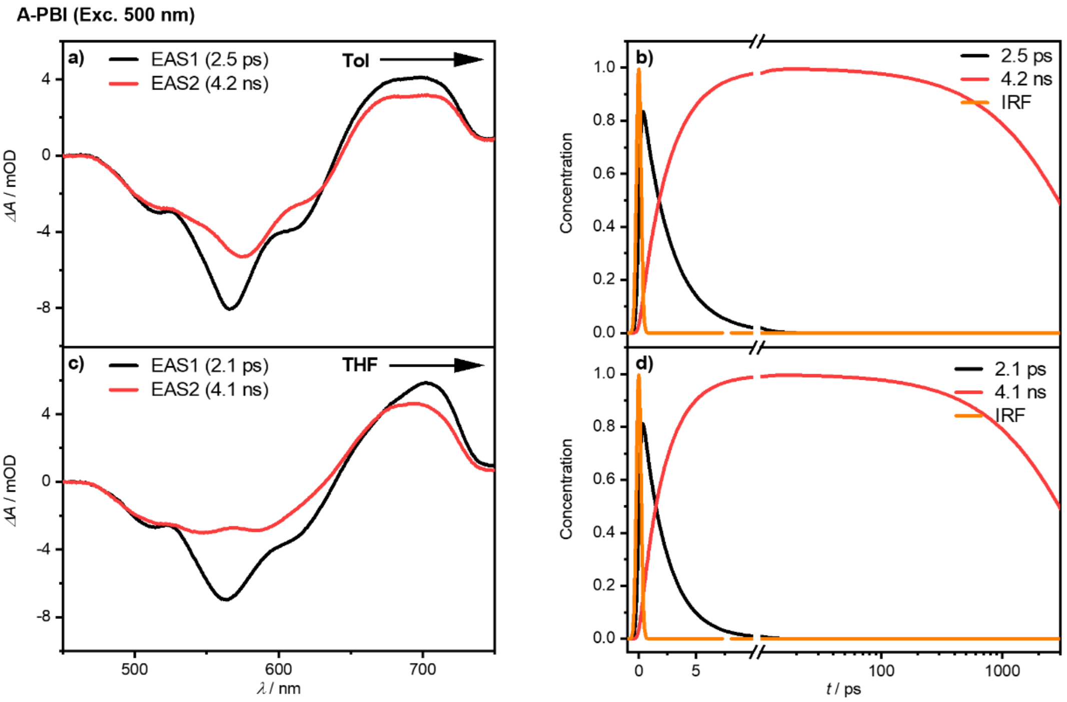

Supplementary Figure 21. Time-resolved characterization. Evolution-associated spectra **a), c)** and the corresponding population profiles **b), d)** of **A-PBI** in Tol **a), b)** and THF **c), d)** obtained by global fitting analysis with 500 nm excitation.

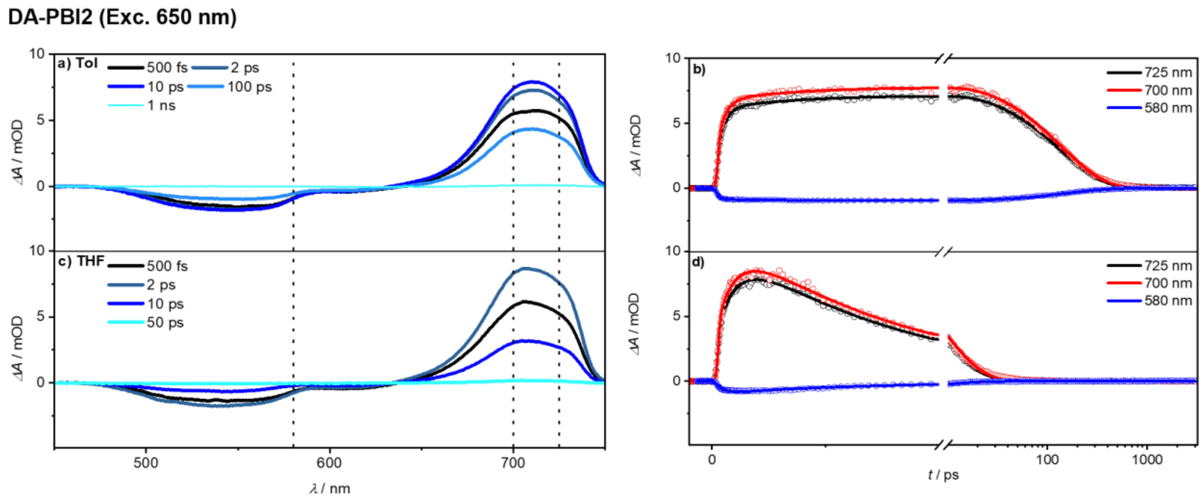

Supplementary Figure 22. Time-resolved characterization. Transient absorption spectra of **DA-PBI2** in Tol **a)** and THF **c)** at selected delay times with 650 nm excitation. Transient absorption kinetic traces in Tol **b)** and THF **d)** probed at 580, 700 and 725 nm and the corresponding single-wavelength fitting line.

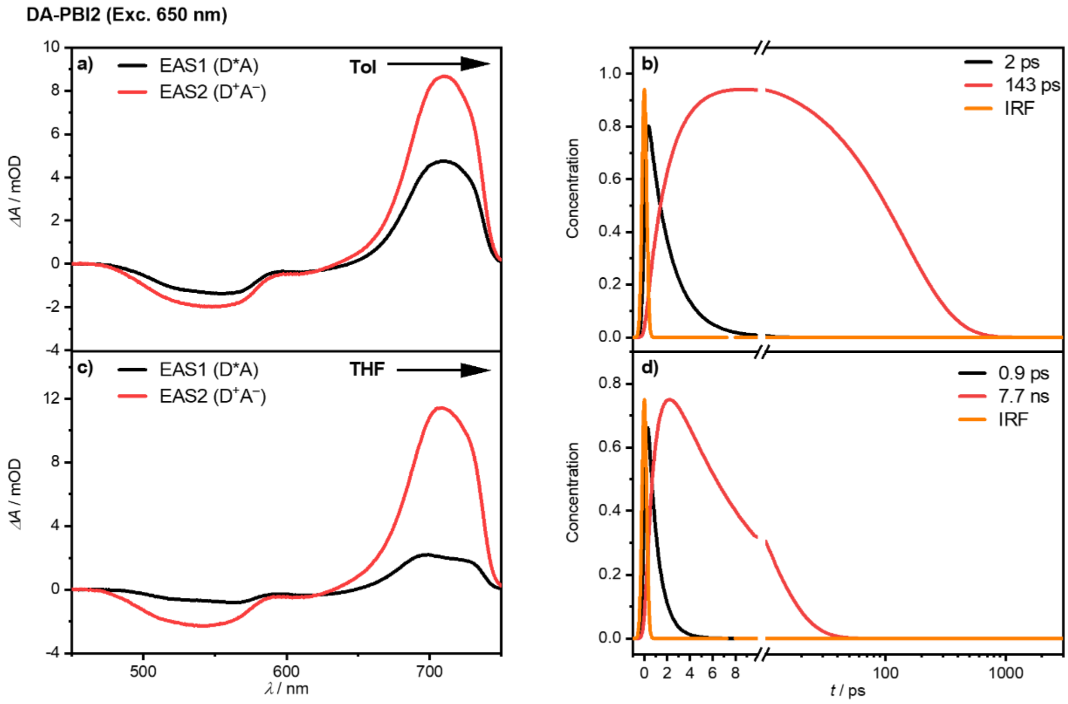

Supplementary Figure 23. Time-resolved characterization. Evolution-associated spectra **a), c)** and the corresponding population profiles **b), d)** of **DA-PBI2** in Tol **a), b)** and THF **c), d)** obtained by global fitting analysis with 650 nm excitation.

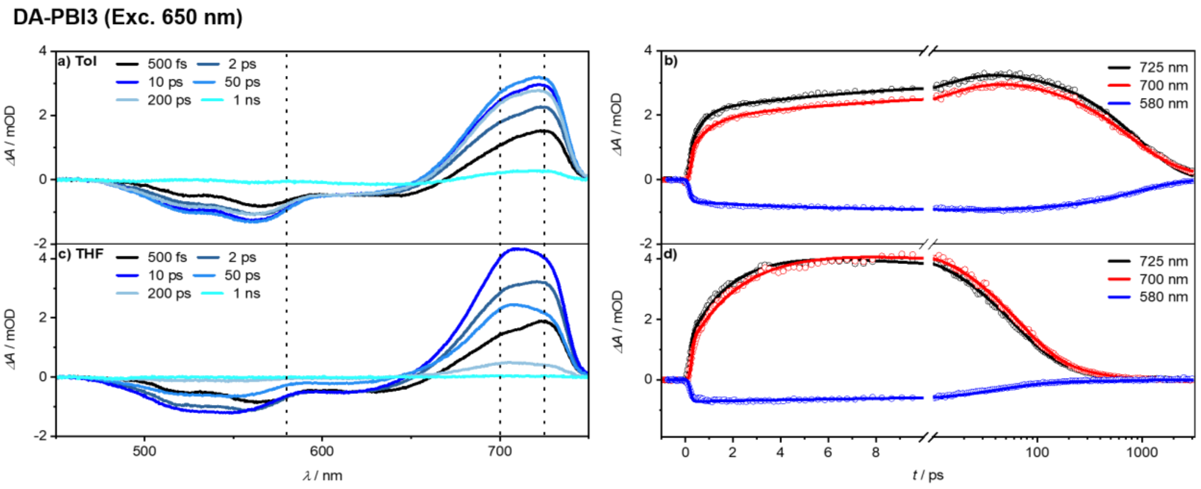

Supplementary Figure 24. Time-resolved characterization. Transient absorption spectra of **DA-PBI3** in Tol **a)** and THF **c)** at selected delay times with 650 nm excitation. Transient absorption kinetic traces in Tol **b)** and THF **d)** probed at 580, 700 and 725 nm and the corresponding single-wavelength fitting line.

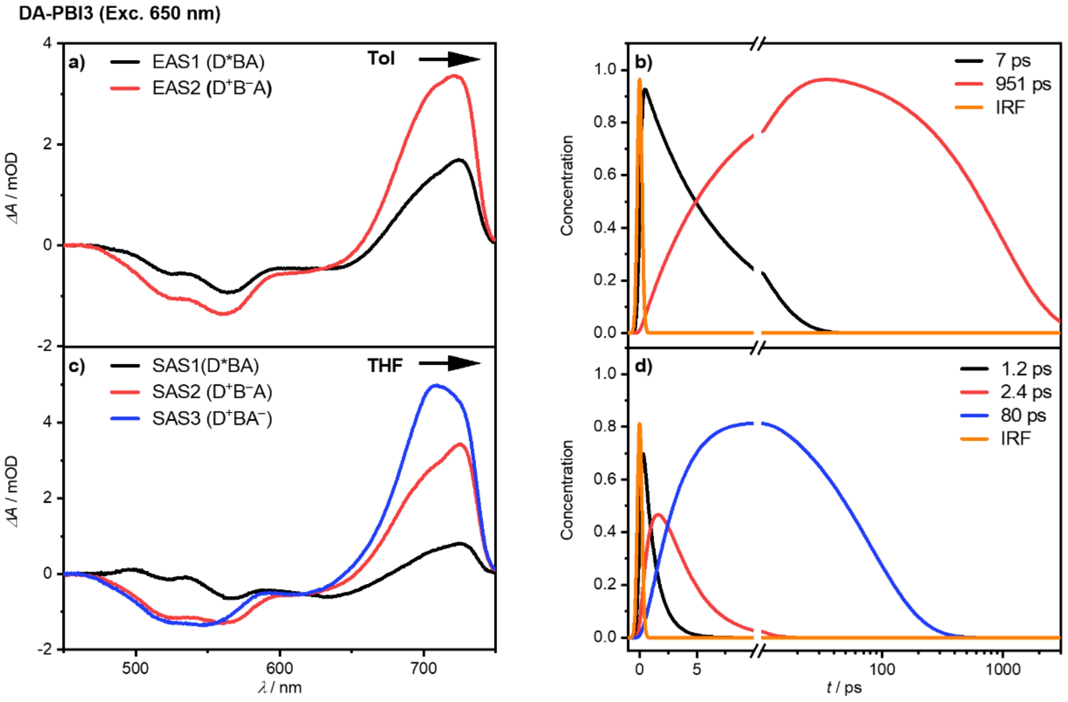

Supplementary Figure 25. Time-resolved characterization. Evolution-associated spectra **a)**, species-associated spectra **c)** and the corresponding population profiles **b), d)** of **DA-PBI3** in Tol **a), b)** and THF **c), d)** obtained by global fitting analysis with 650 nm excitation.

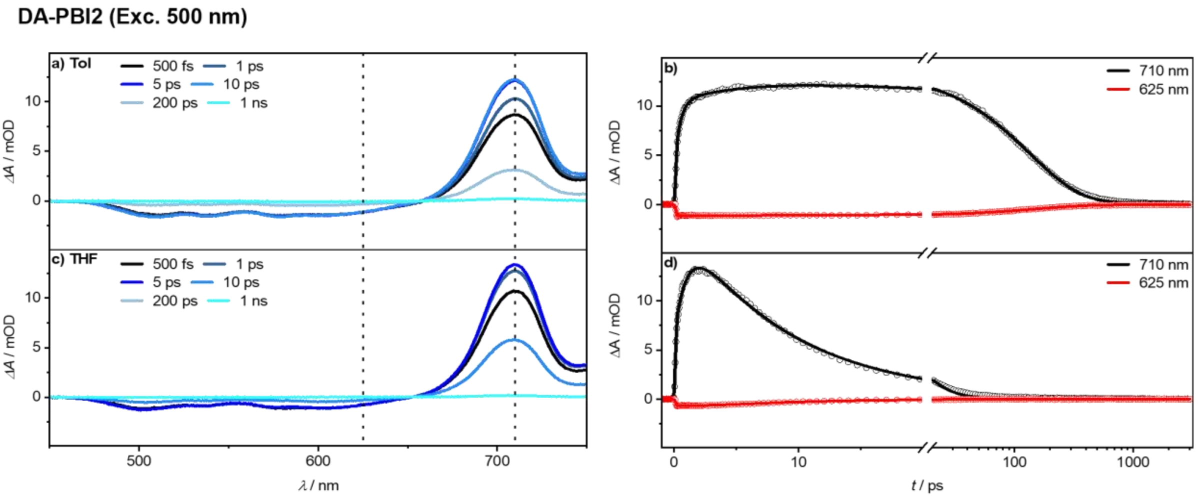

Supplementary Figure 26. Time-resolved characterization. Transient absorption spectra of **DA-PBI2** in Tol **a)** and THF **c)** at selected delay times with 500 nm excitation; transient absorption kinetic traces in Tol **b)** and THF **d)** probed at 625 and 710 nm and the corresponding single-wavelength fitting line.

The emergence and decay of the ESA band, centered around 710 nm, implies efficient ET processes with time constants of 2.6 and 1 ps, respectively, along with CR processes featuring time constants of 147 and 9 ps in Tol and THF, respectively.

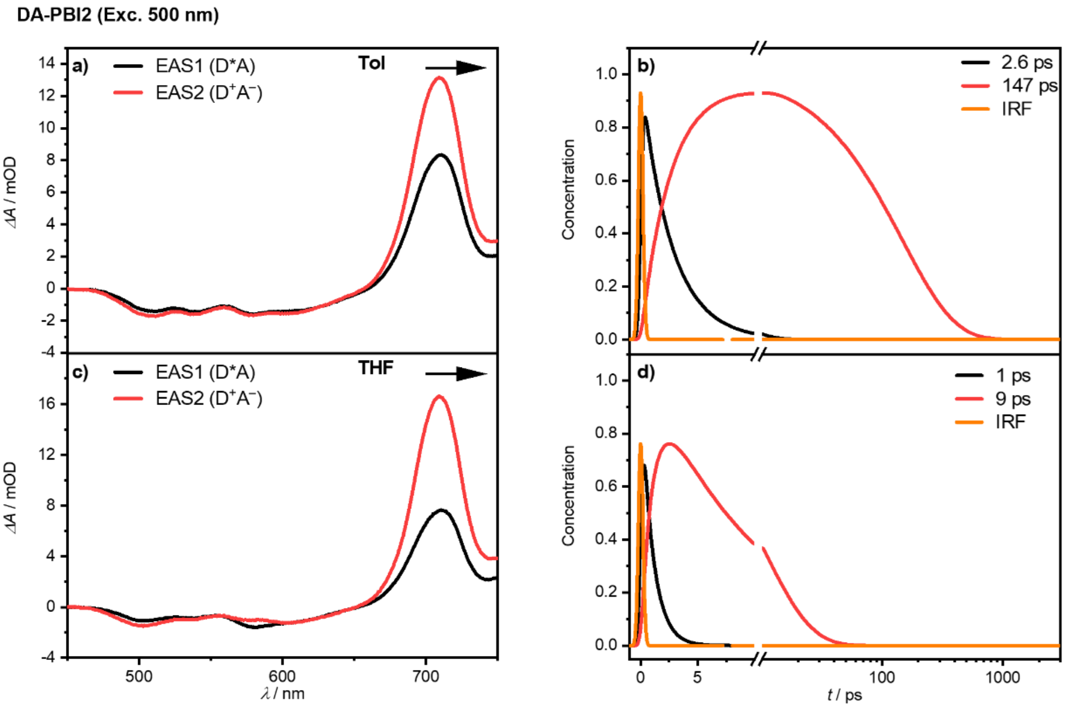

Supplementary Figure 27. Time-resolved characterization. Evolution-associated spectra **a), c)** and the corresponding population profiles **b), d)** of **DA-PBI2** in Tol **a), b)** and THF **c), d)** obtained by global fitting analysis with 500 nm excitation.

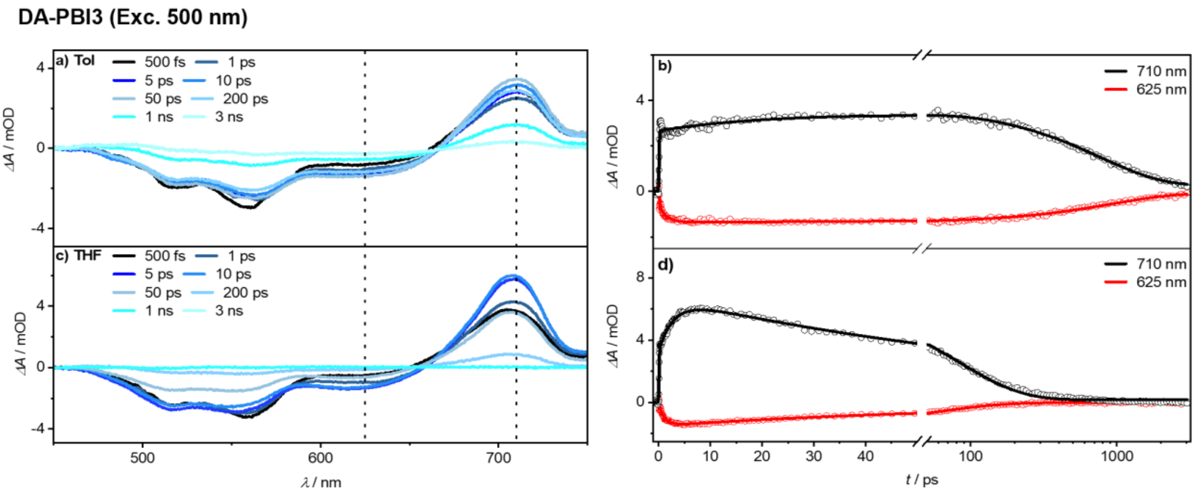

Supplementary Figure 28. Time-resolved characterization. Transient absorption spectra of **DA-PBI3** in Tol **a)** and THF **c)** at selected delay times with 500 nm excitation; transient absorption kinetic traces in Tol **b)** and THF **d)** probed at 625 and 710 nm and the corresponding single-wavelength fitting line.

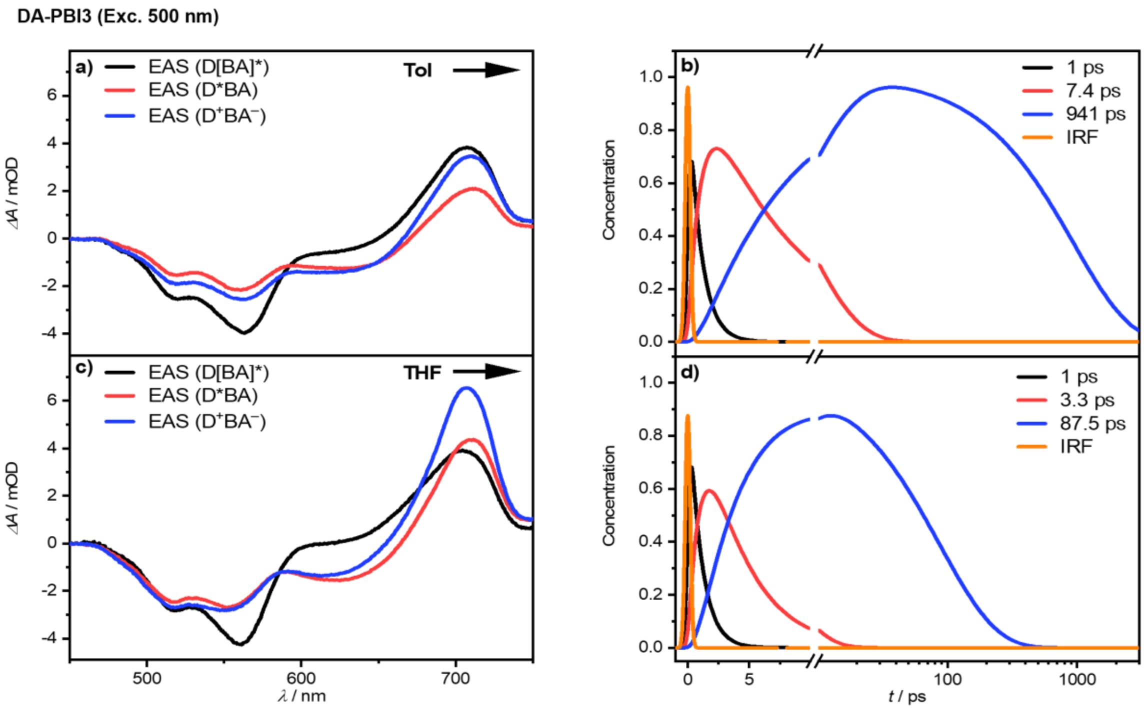

Supplementary Figure 29. Time-resolved characterization. Evolution-associated spectra **a), c)** and the corresponding population profiles **b), d)** of **DA-PBI3** in Tol **a), b)** and THF **c), d)** obtained by global fitting analysis with 500 nm excitation.

Global fitting analysis with a sequential model (Supplementary Fig. 29) yields solvent polarity-dependent CS time constants of τ_{CS} = 7.4 and 3 ps, as well as CR lifetimes of τ_{CR} = 941 and 87.5 ps in Tol and THF, respectively and a solvent polarity-independent EnT time constant of 1 ps.

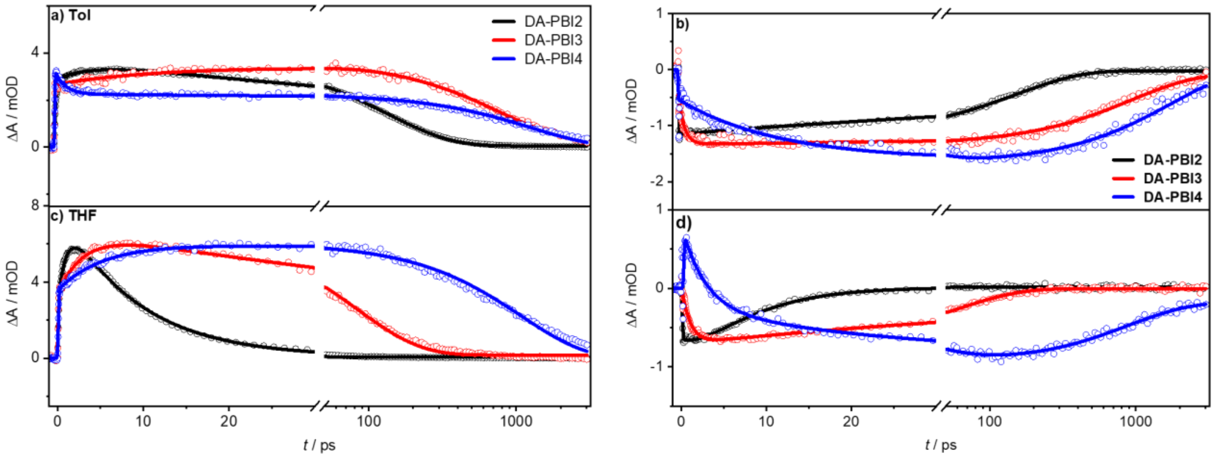

Supplementary Figure 30. Time-resolved characterization. The comparison of transient absorption kinetic traces at 710 nm, which are related to electron transfer process in Tol **a)** and THF **c)** for **DA-PBI2**, **DA-PBI3**, and **DA-PBI4**. The comparison of transient absorption kinetic traces at 625 nm, which are related to energy transfer process in Tol **b)** and THF **d)** for **DA-PBI2**, **DA-PBI3**, and **DA-PBI4**.

9. NMR Spectroscopy

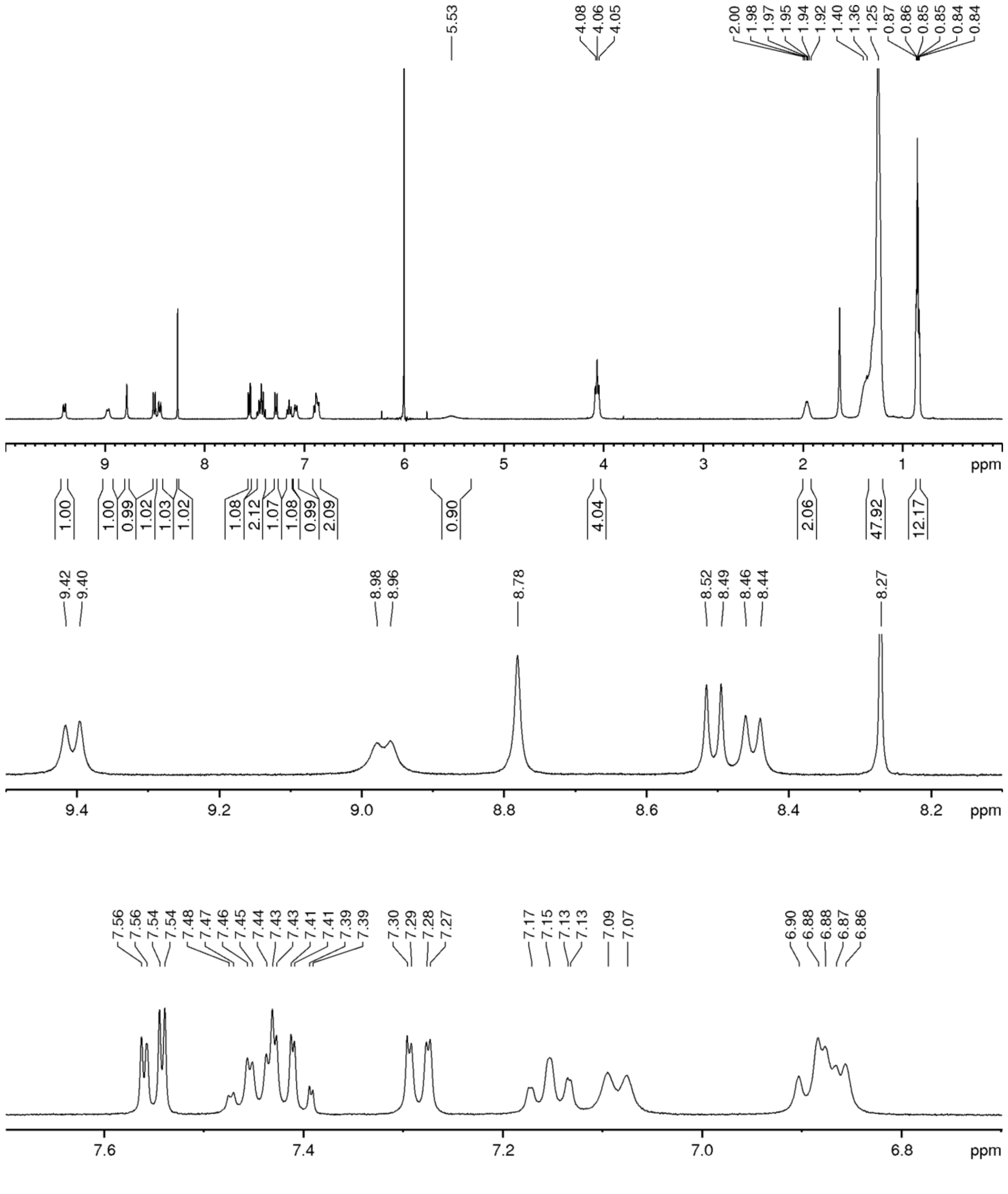

Supplementary Figure 31. NMR spectroscopic characterization. ^{1}H NMR spectrum (400 MHz, 295 K, TCE-d_2) of PBI **3**.

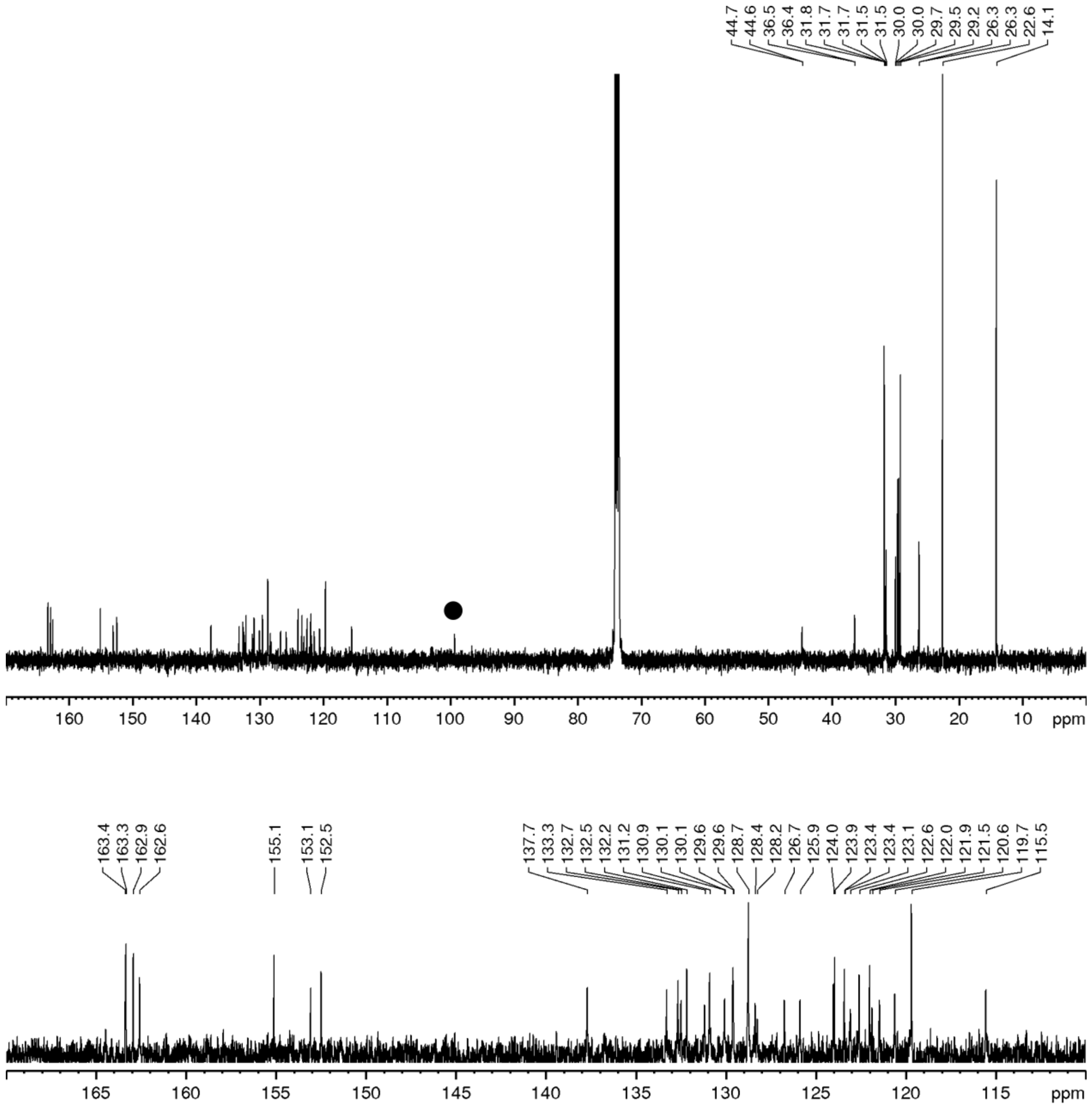

Supplementary Figure 32. NMR spectroscopic characterization. ^{13}C NMR spectrum (101 MHz, 295 K, TCE-d_2) of PBI **3**. Residual signal is marked as followed: tetrachloromethane (●) present in deuterated 1,1,2,2-tetrachloroethane.[29]

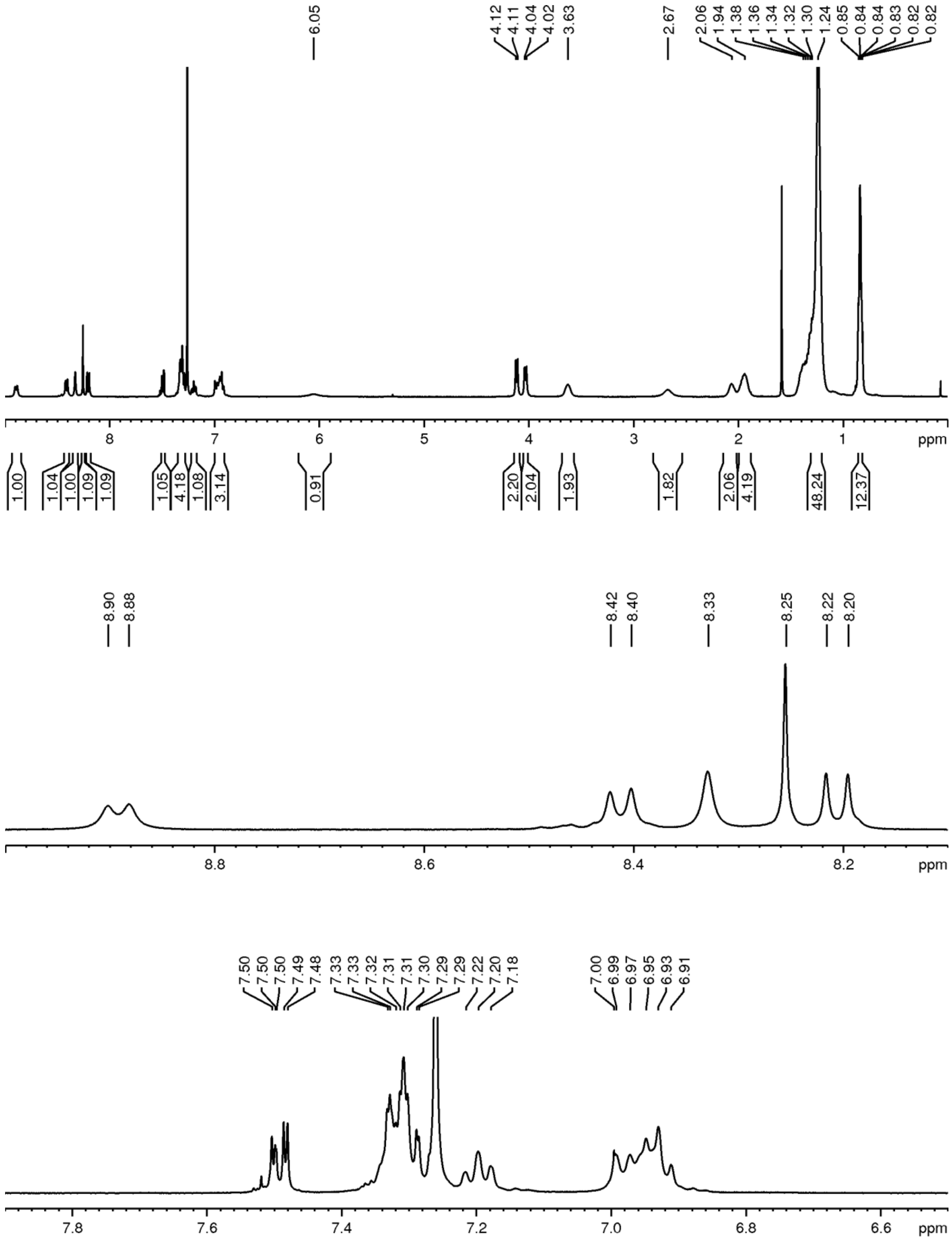

Supplementary Figure 33. NMR spectroscopic characterization. ^{1}H NMR spectrum (400 MHz, 295 K, $CDCl_3$) of PBI **4**.

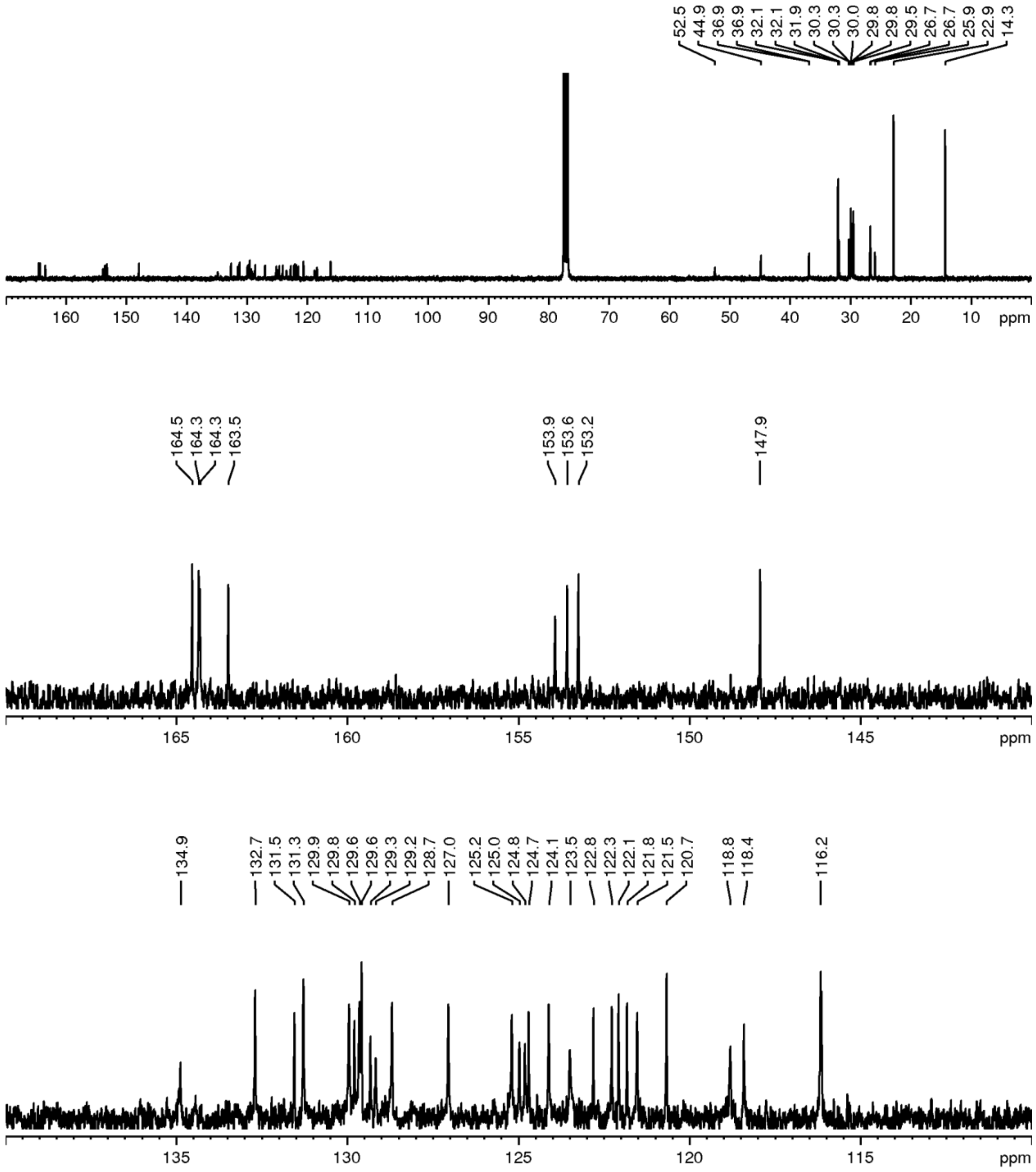

Supplementary Figure 34. NMR spectroscopic characterization. ^{13}C NMR spectrum (101 MHz, 295 K, TCE-d_2) of PBI **4**.

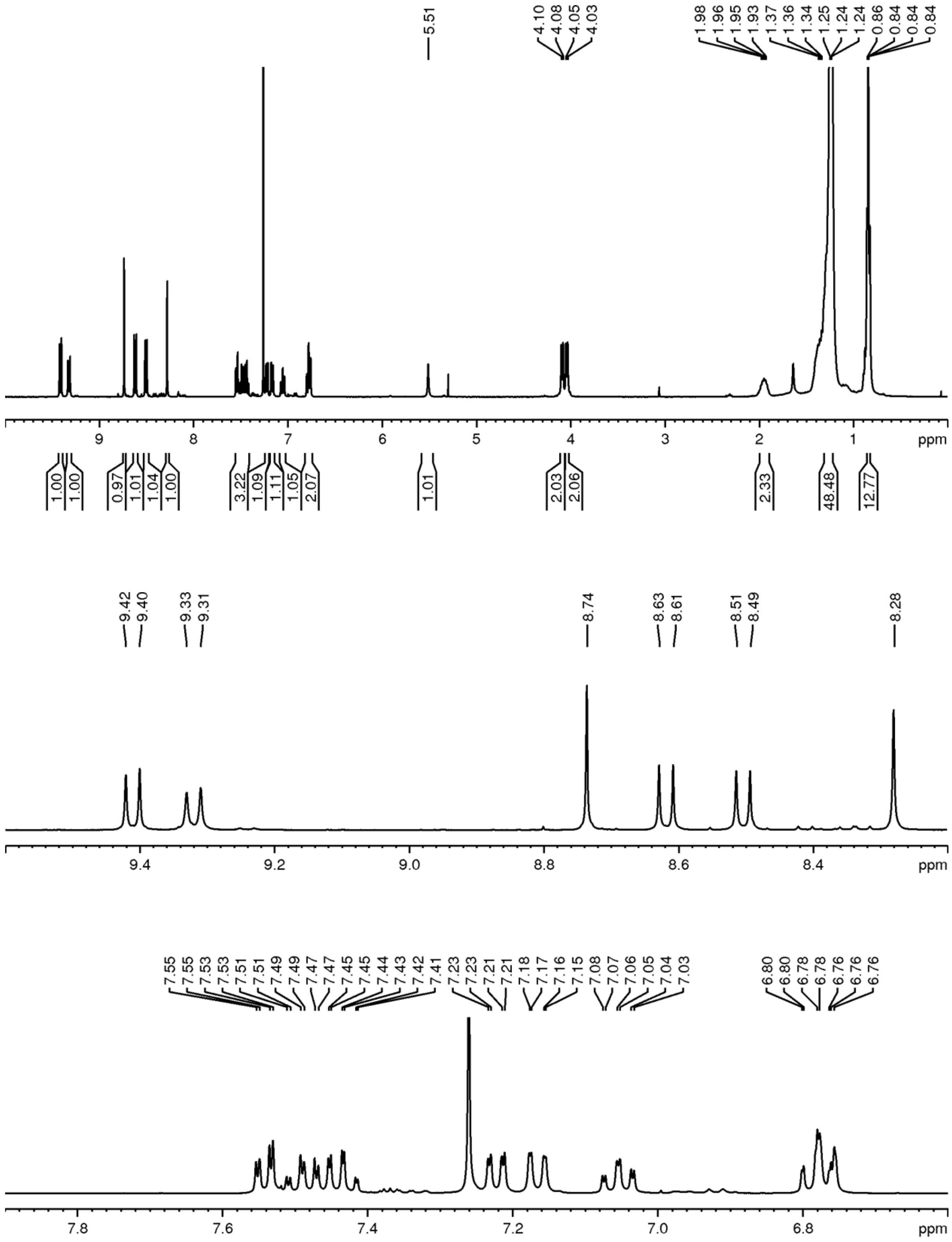

Supplementary Figure 35. NMR spectroscopic characterization. ^{1}H NMR spectrum (400 MHz, 295 K, $CDCl_3$) of PBI **5**.

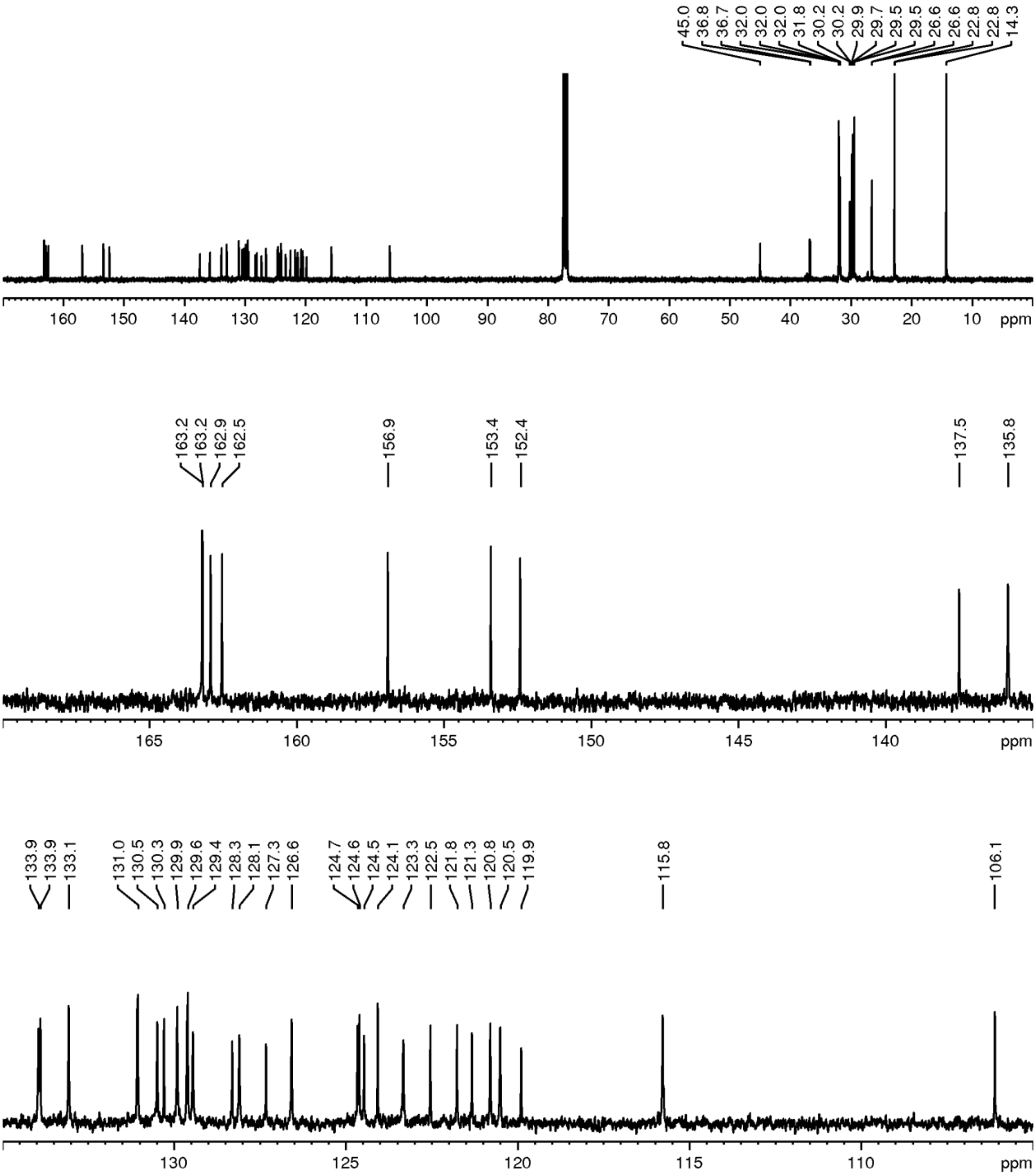

Supplementary Figure 36. NMR spectroscopic characterization. ^{13}C NMR spectrum (101 MHz, 295 K, TCE-d_2) of PBI **5**.

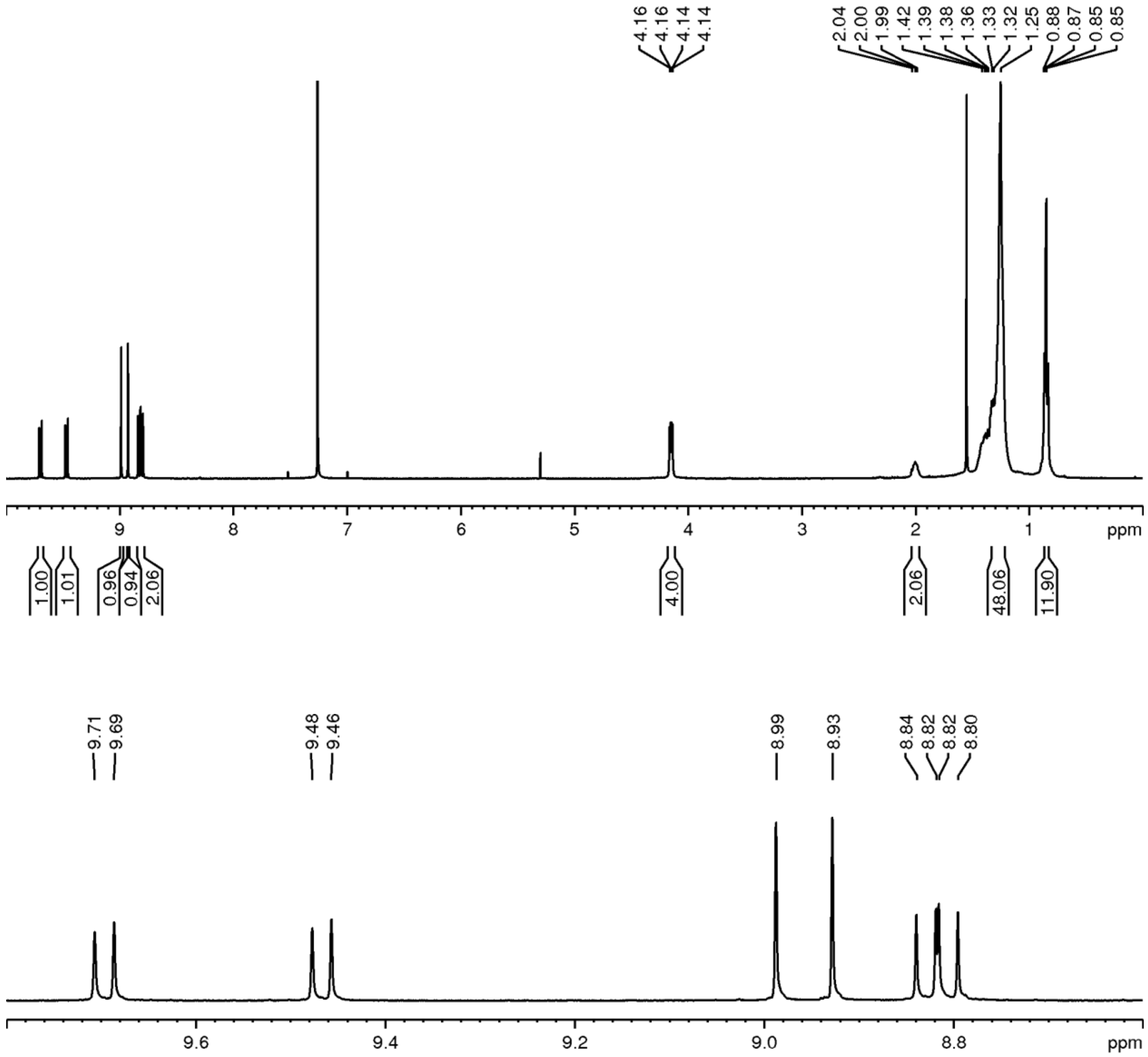

Supplementary Figure 37. NMR spectroscopic characterization. ^{1}H NMR spectrum (400 MHz, 295 K, $CDCl_3$) of PBI **8**.

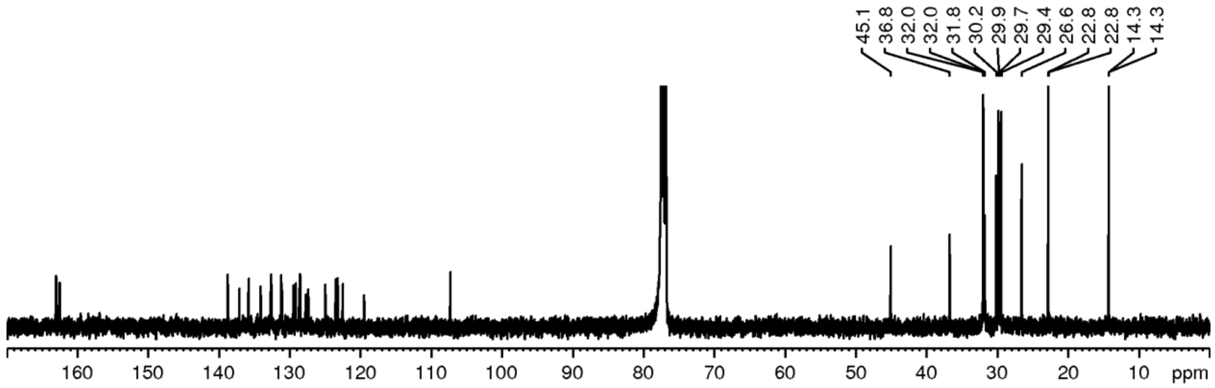

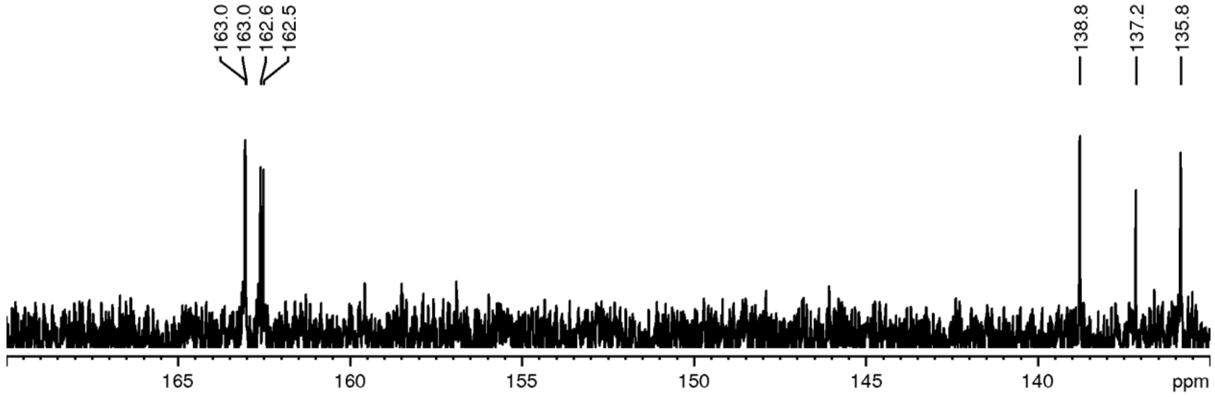

a

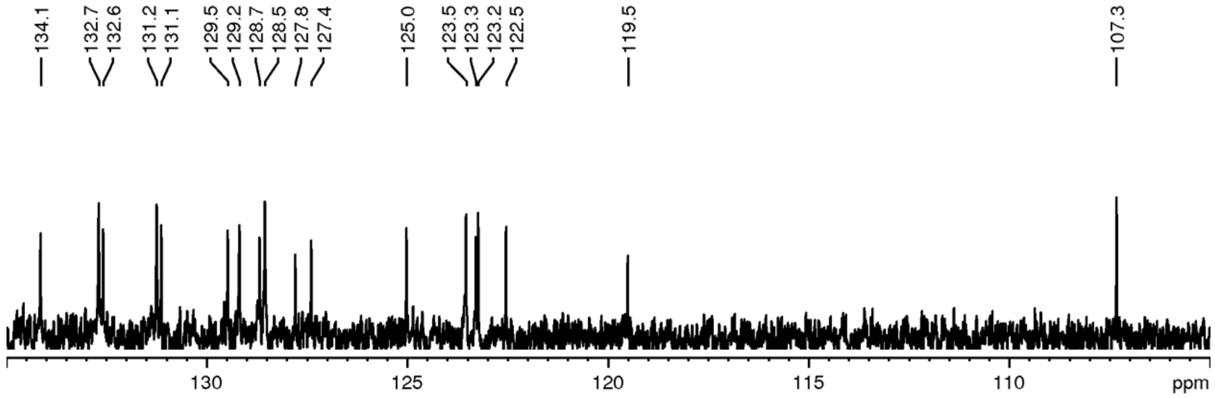

Supplementary Figure 38. NMR spectroscopic characterization. ^{13}C NMR spectrum (101 MHz, 295 K, $CDCl_3$) of PBI **8**.

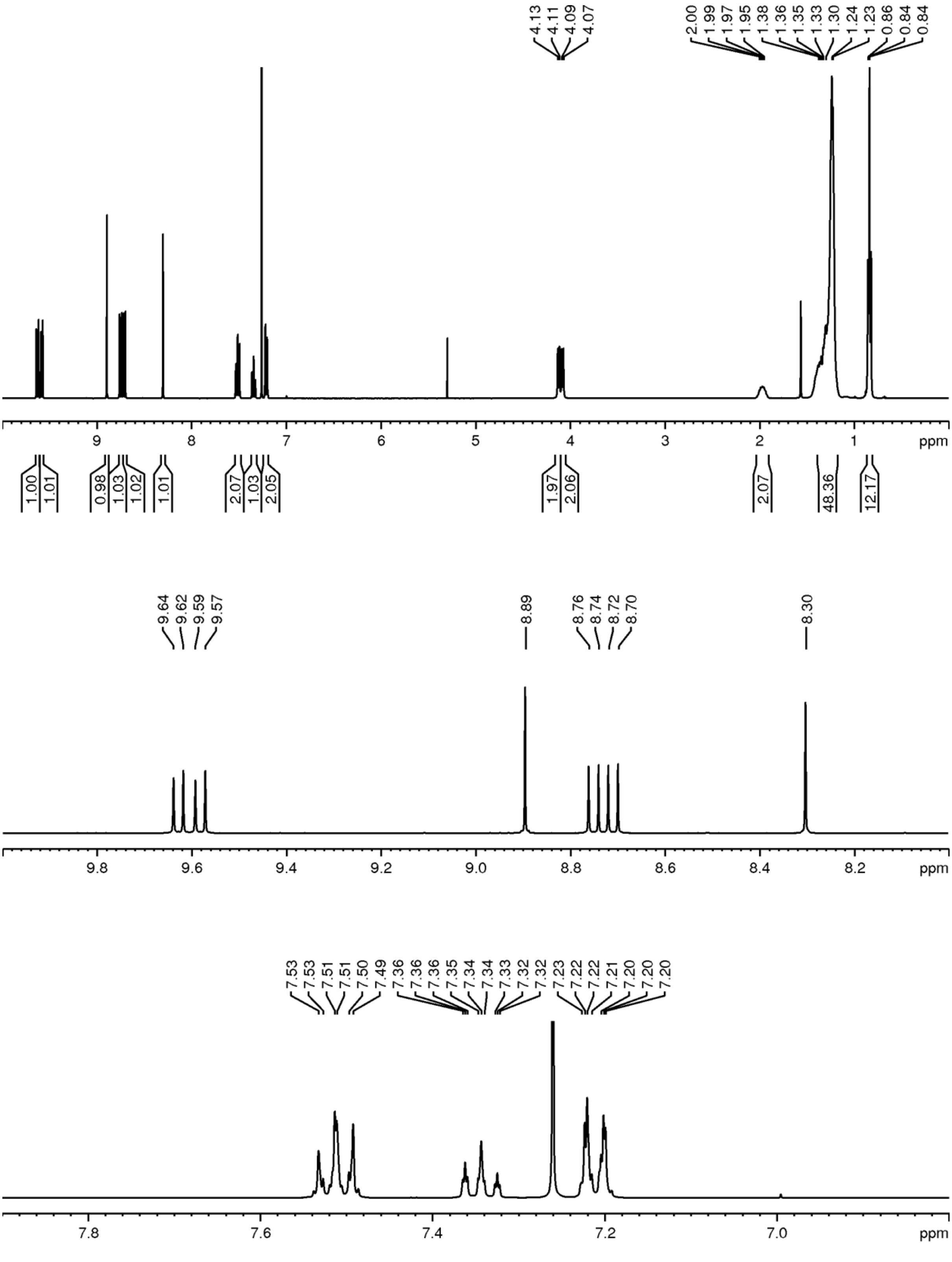

Supplementary Figure 39. NMR spectroscopic characterization. ^1H NMR spectrum (400 MHz, 295 K, $CDCl_3$) of reference molecule **A-PBI**.

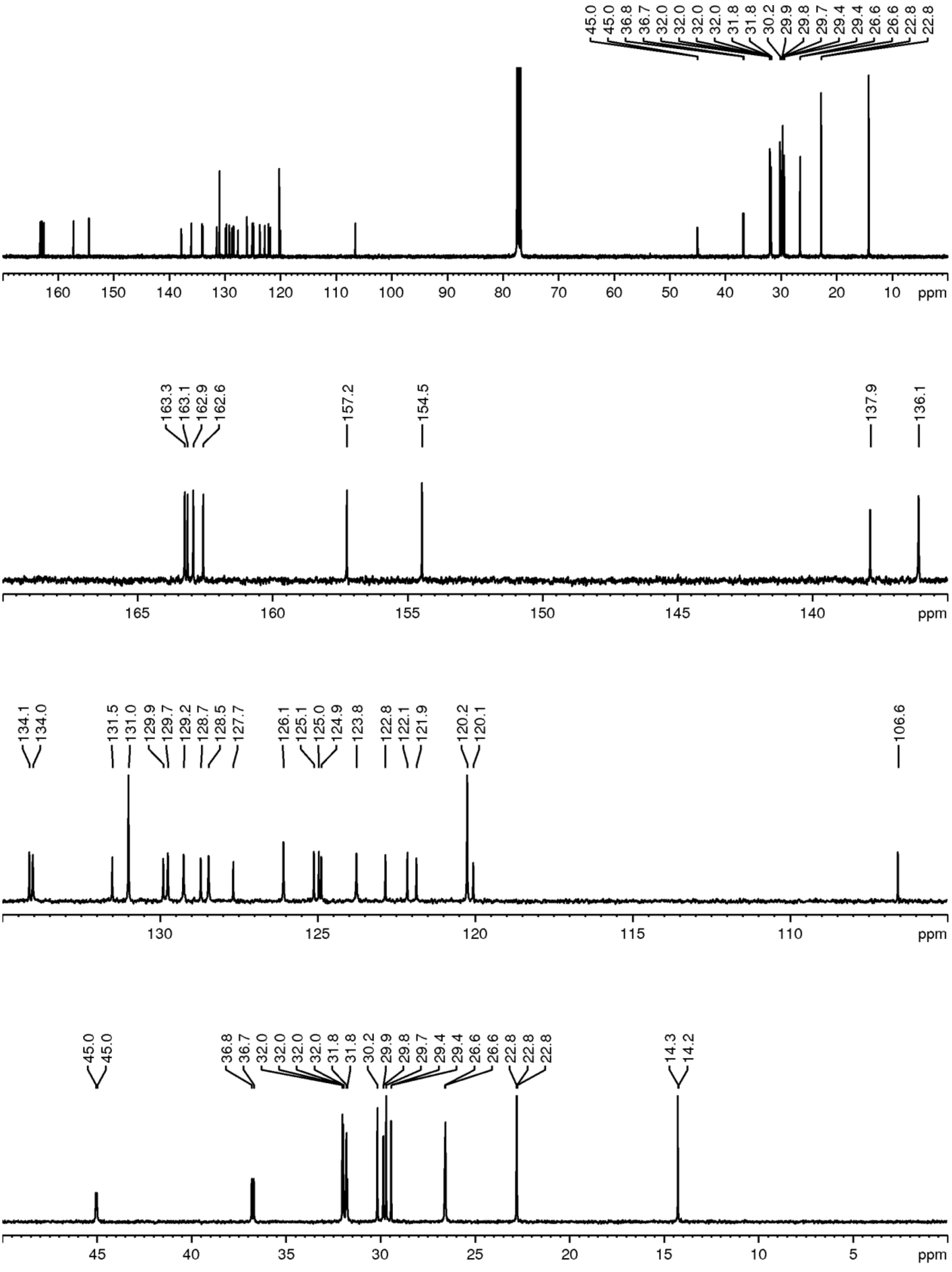

Supplementary Figure 40. NMR spectroscopic characterization. ^{13}C NMR spectrum (101 MHz, 295 K, $CDCl_3$) of reference molecule **A-PBI**.

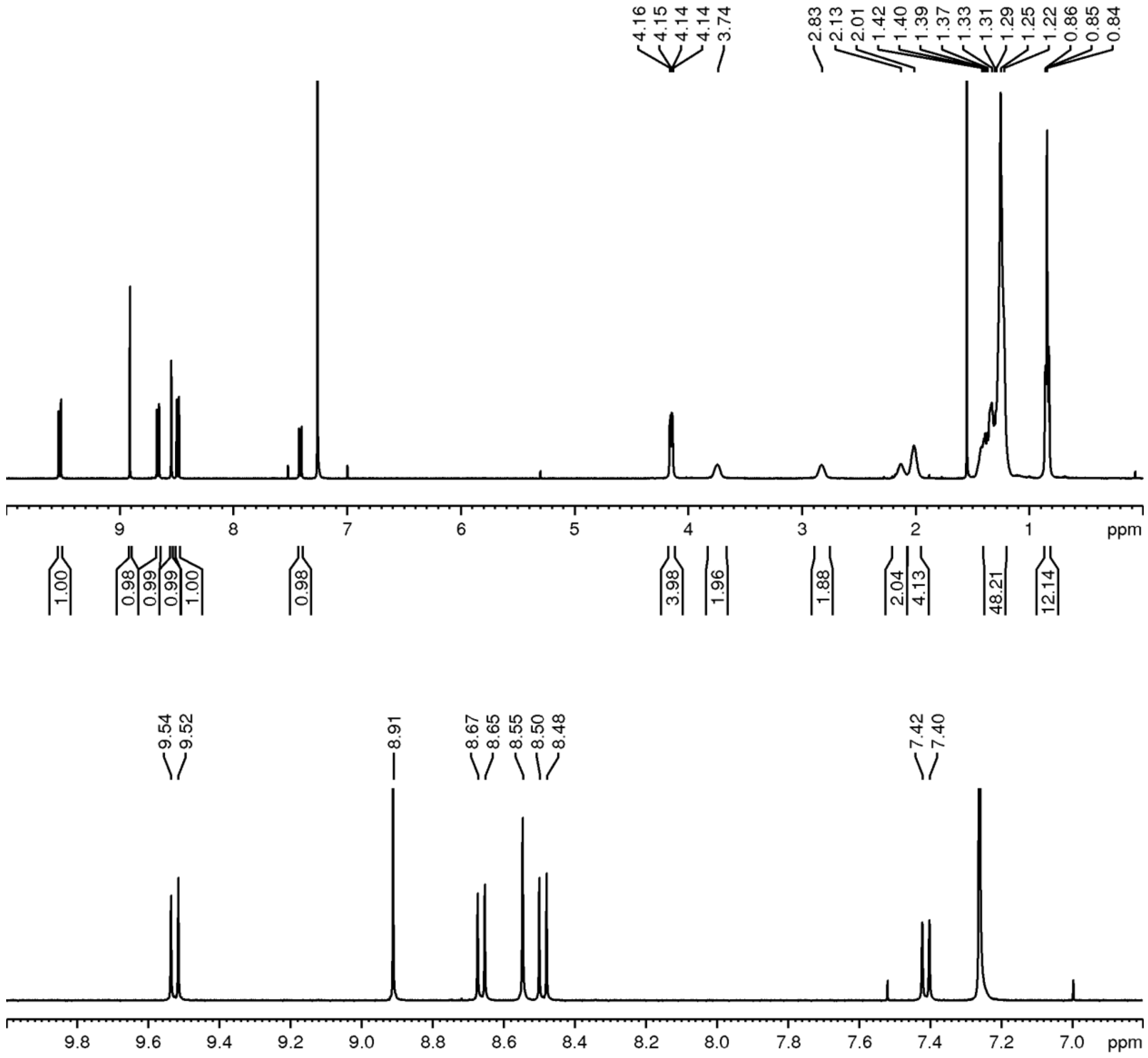

Supplementary Figure 41. NMR spectroscopic characterization. ^{1}H NMR spectrum (400 MHz, 295 K, $CDCl_3$) of PBI **7**.

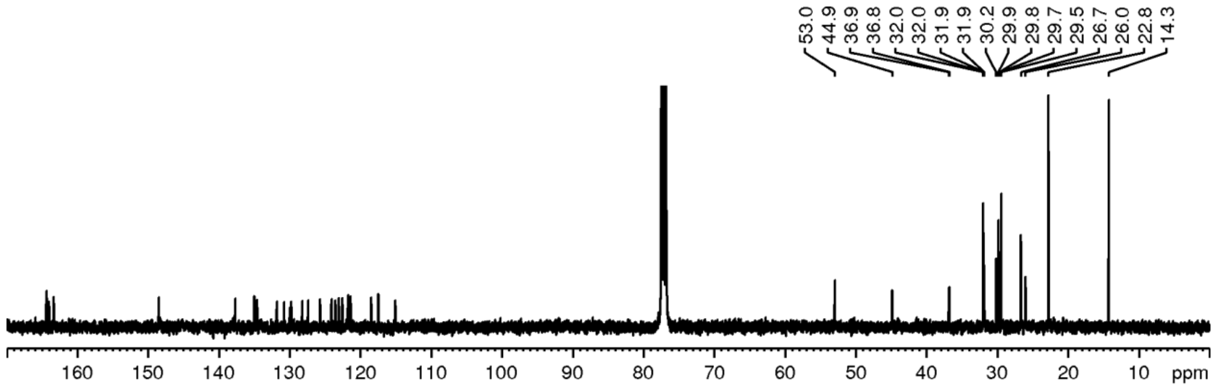

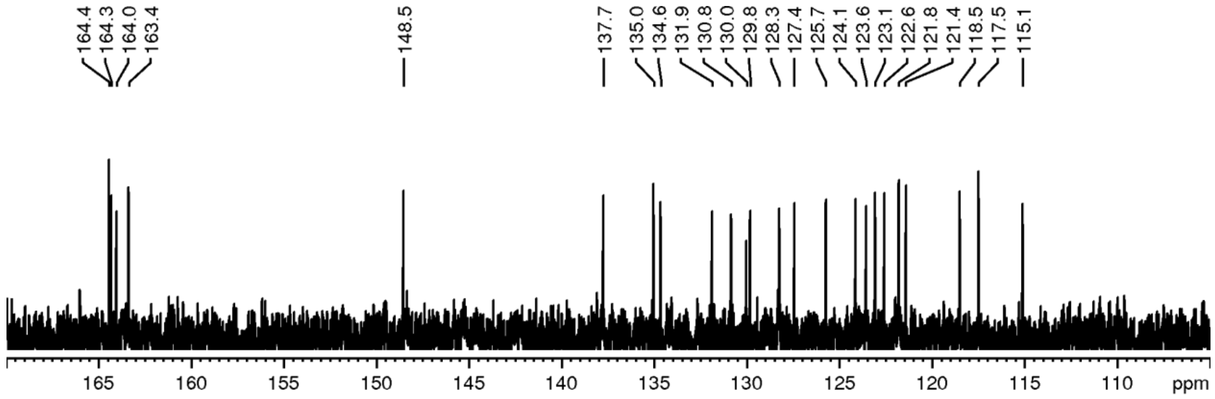

Supplementary Figure 42. NMR spectroscopic characterization. ^{13}C NMR spectrum (101 MHz, 295 K, $CDCl_3$) of PBI **7**.

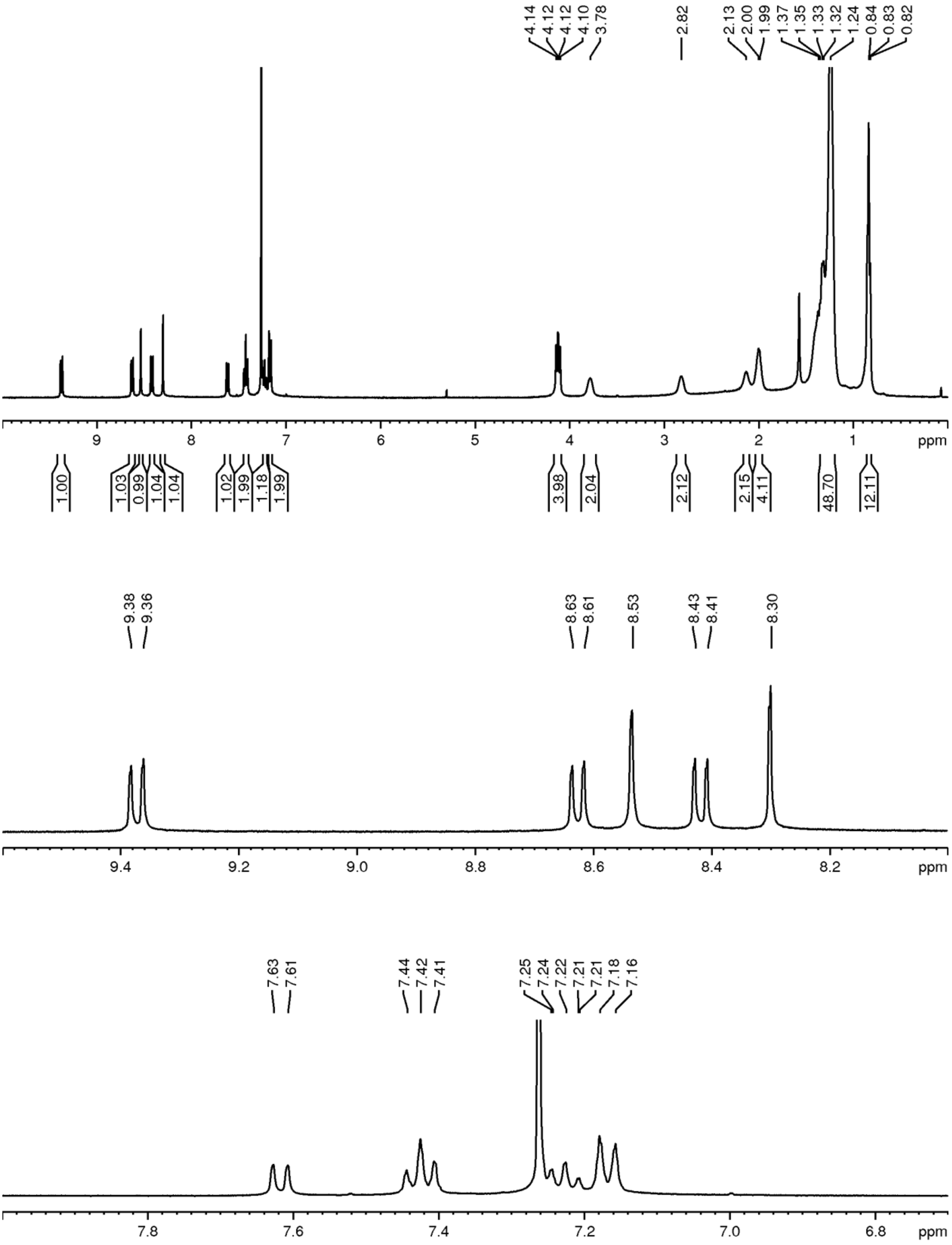

Supplementary Figure 43. NMR spectroscopic characterization. ^{1}H NMR spectrum (400 MHz, 295 K, $CDCl_3$) of reference molecule **D-PBI**.

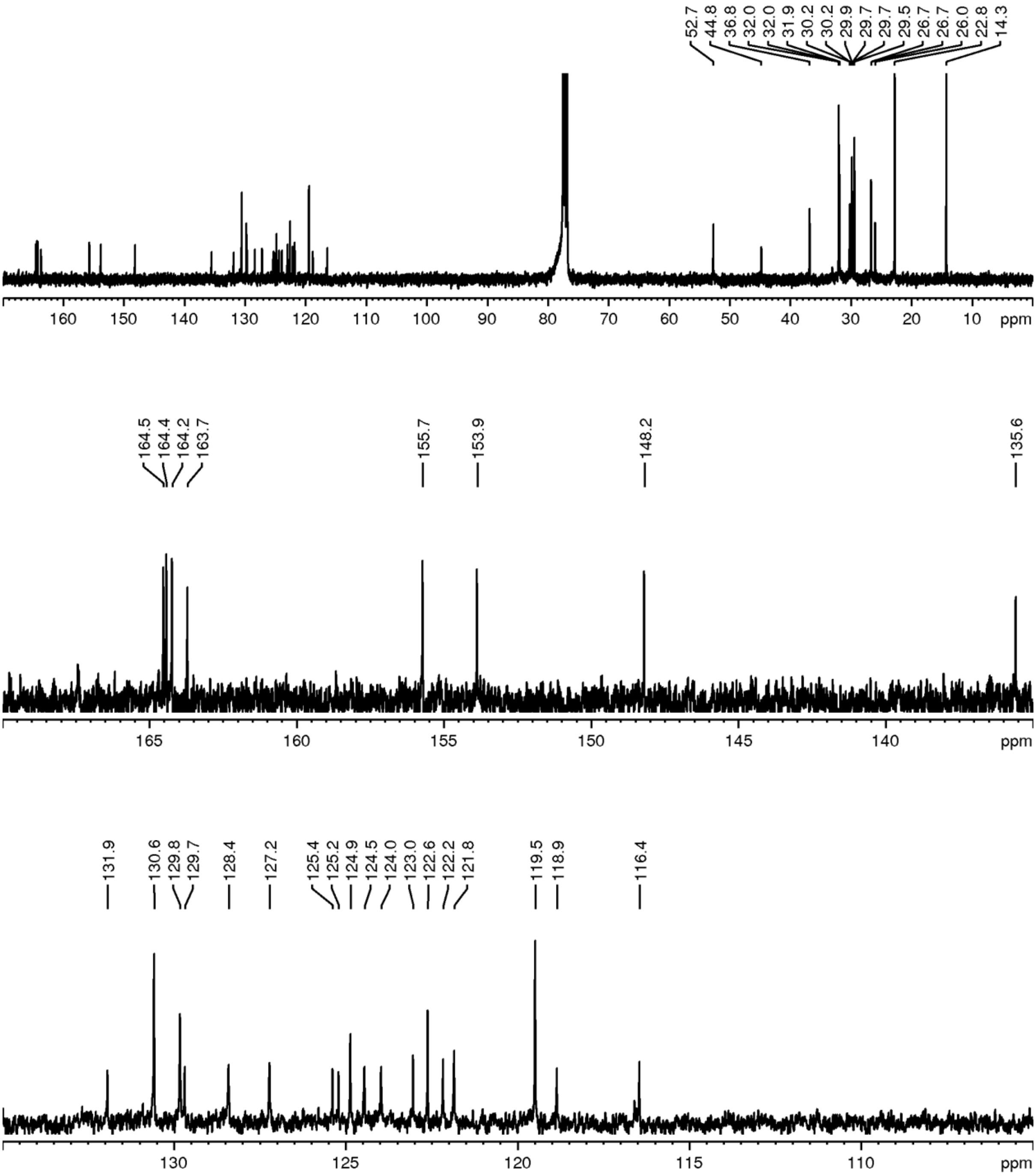

Supplementary Figure 44. NMR spectroscopic characterization. ^{13}C NMR spectrum (101 MHz, 295 K, $CDCl_3$) of reference molecule **D-PBI**.

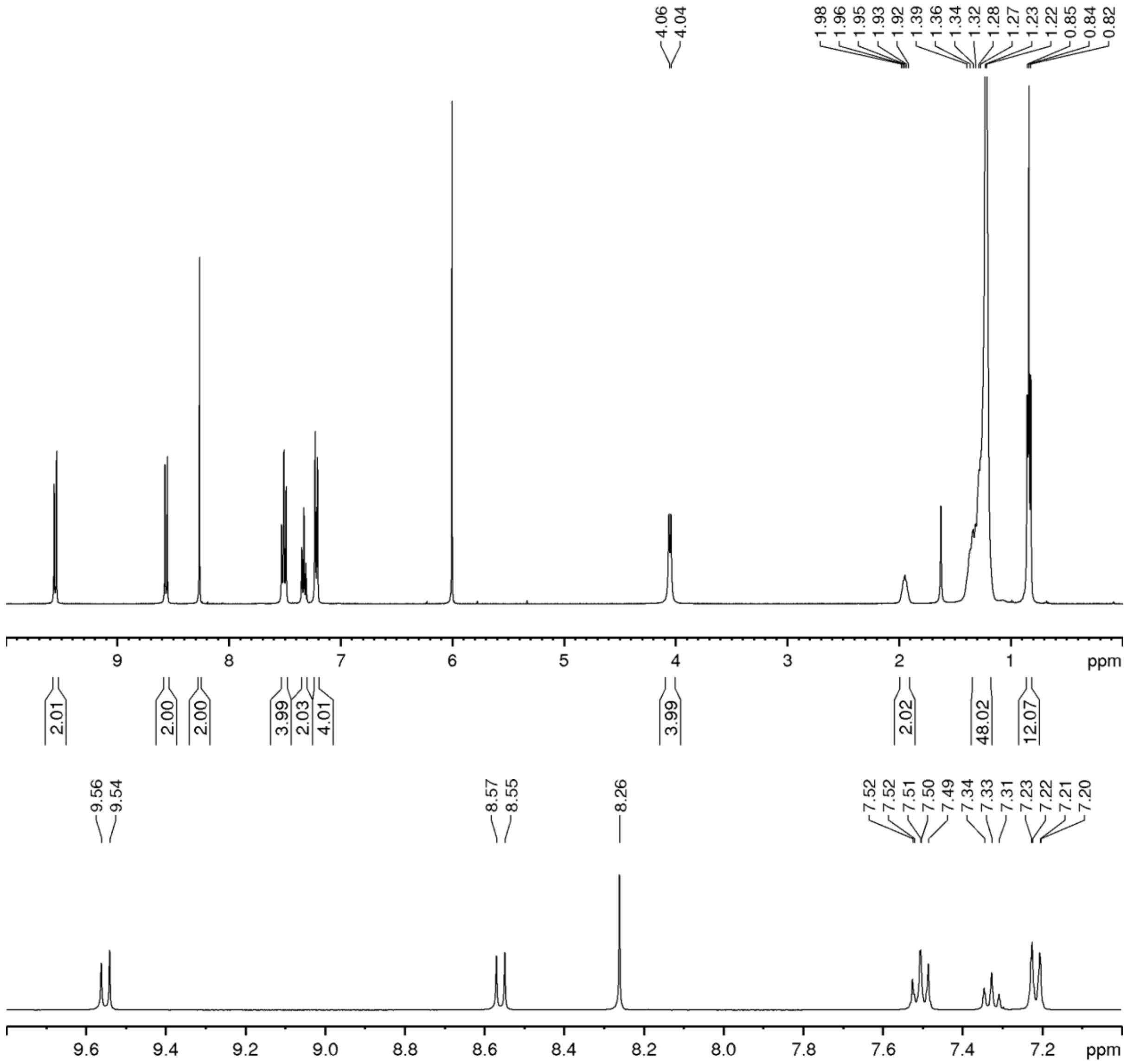

Supplementary Figure 45. NMR spectroscopic characterization. ^1H NMR spectrum (400 MHz, 295 K, TCE-d_2) of reference molecule **PBI-1**.

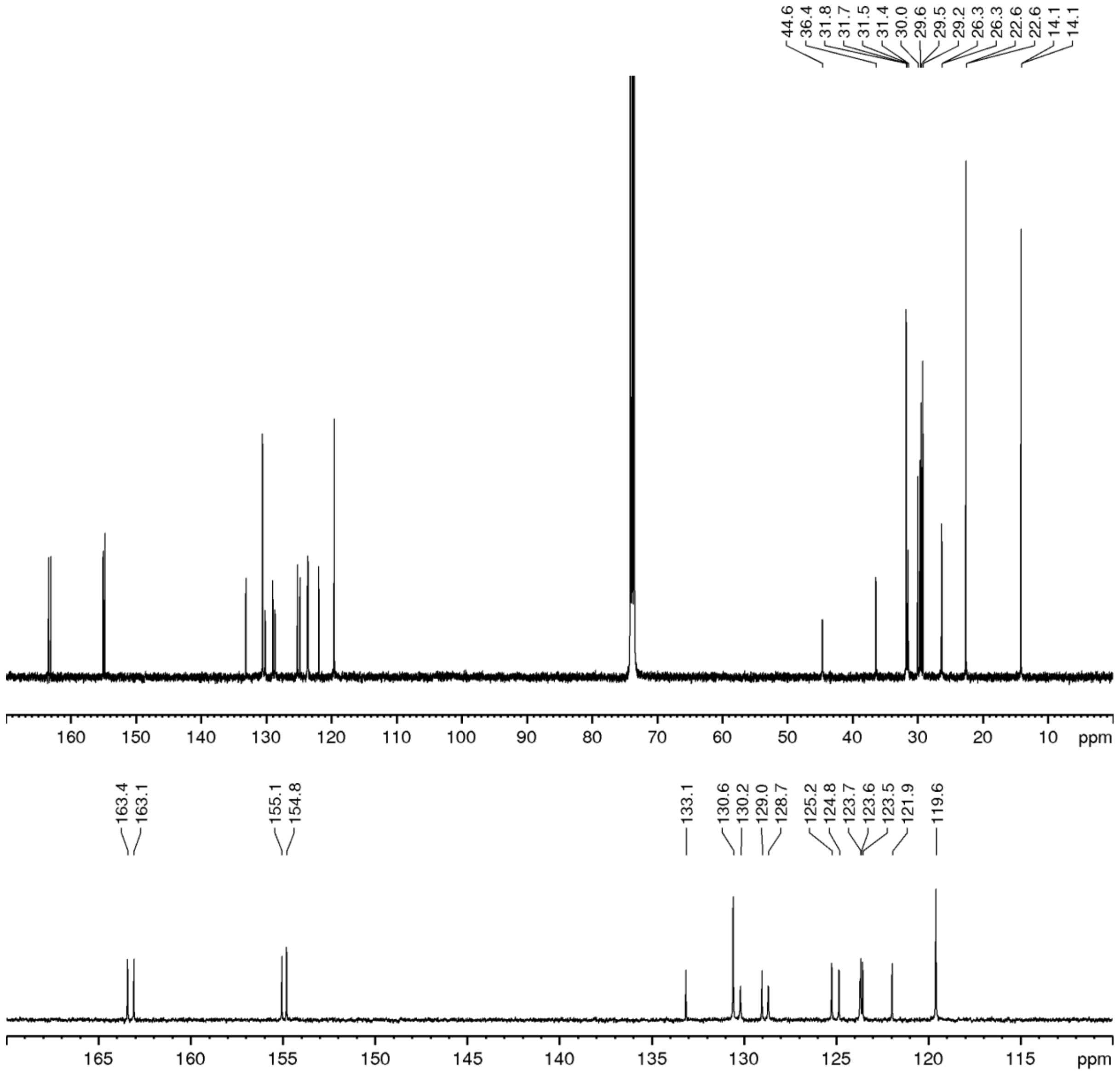

Supplementary Figure 46. NMR spectroscopic characterization. ^{13}C NMR spectrum (101 MHz, 295 K, TCE-d_2) of reference molecule **PBI-1**.

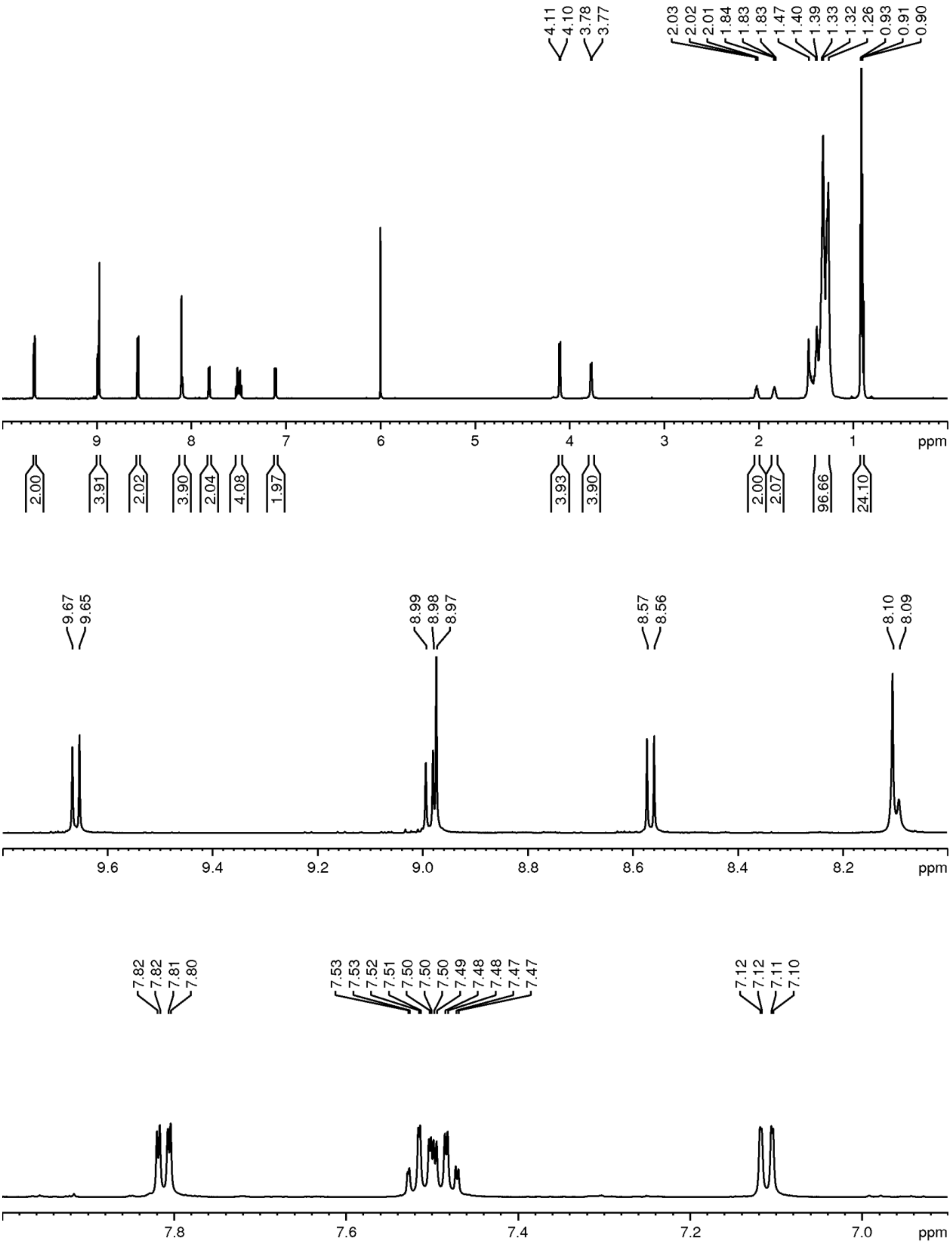

Supplementary Figure 47. NMR spectroscopic characterization. ^{1}H NMR spectrum (600 MHz, 384 K, TCE-d_2) of **Br-PBI2-Br**.

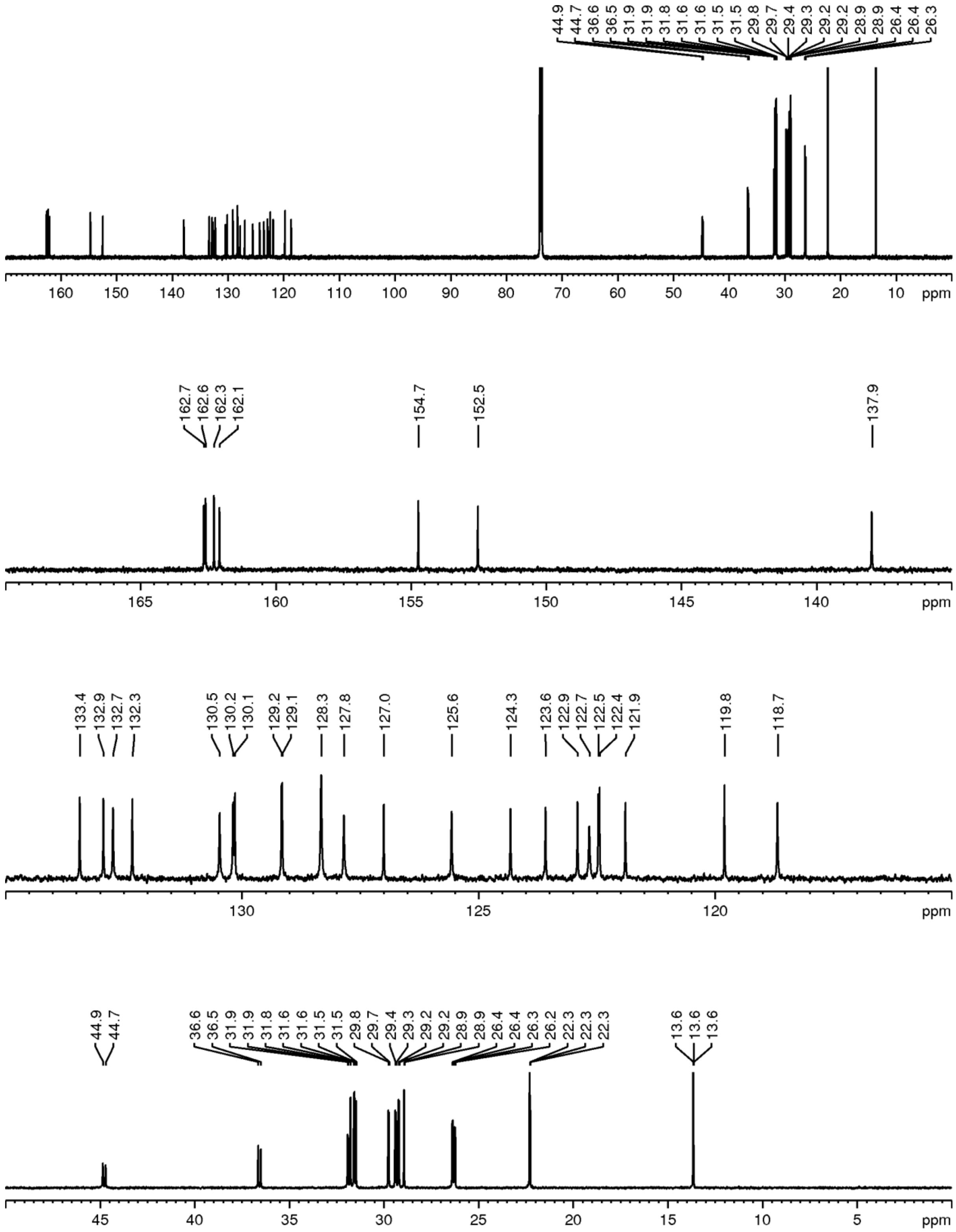

Supplementary Figure 48. NMR spectroscopic characterization. ^{13}C NMR spectrum (151 MHz, 384 K, TCE-d_2) of **Br-PBI2-Br**.

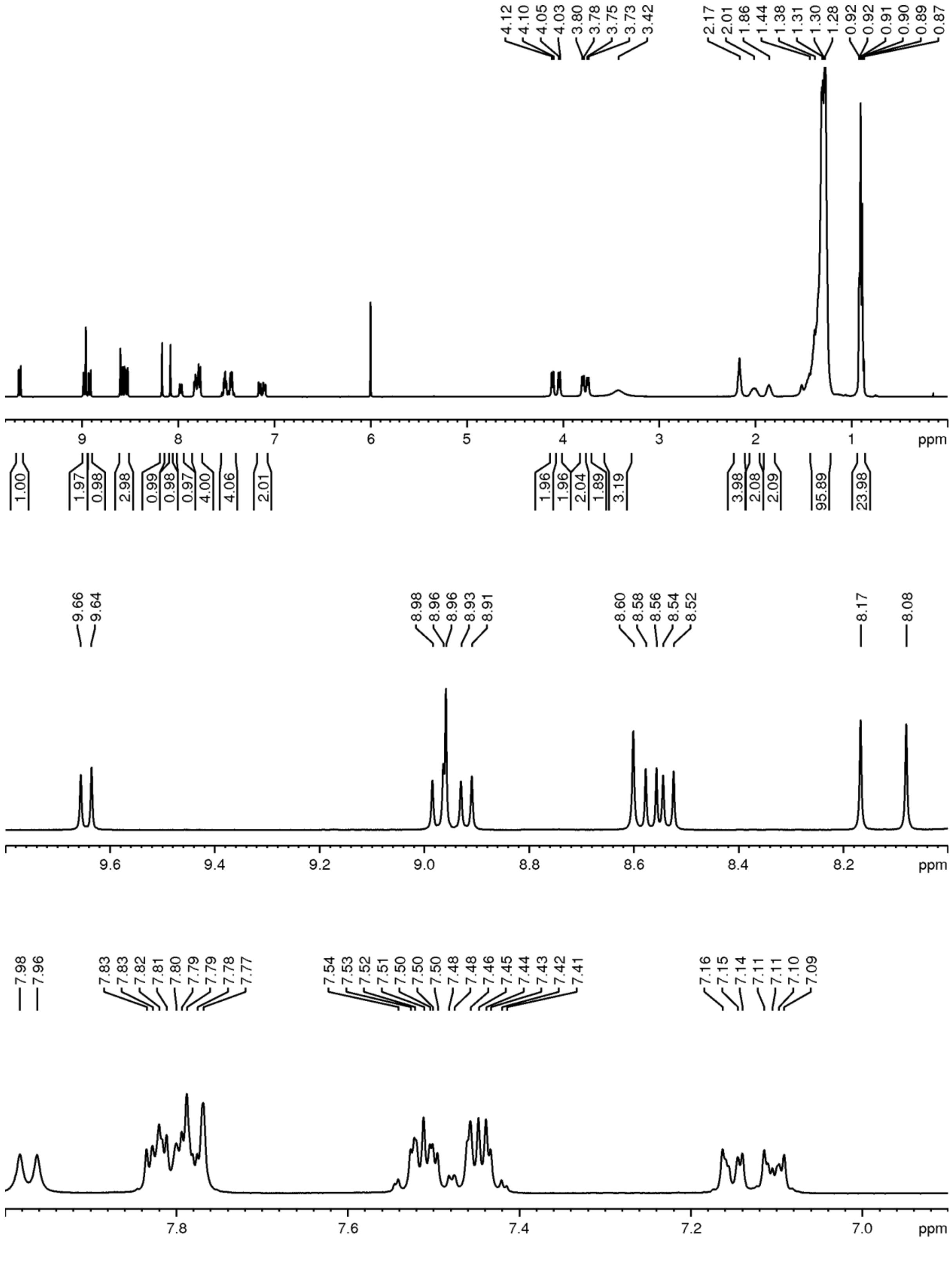

Supplementary Figure 49. NMR spectroscopic characterization. ^{1}H NMR spectrum (600 MHz, 384 K, TCE-d_2) of **D-PBI2-Br.**

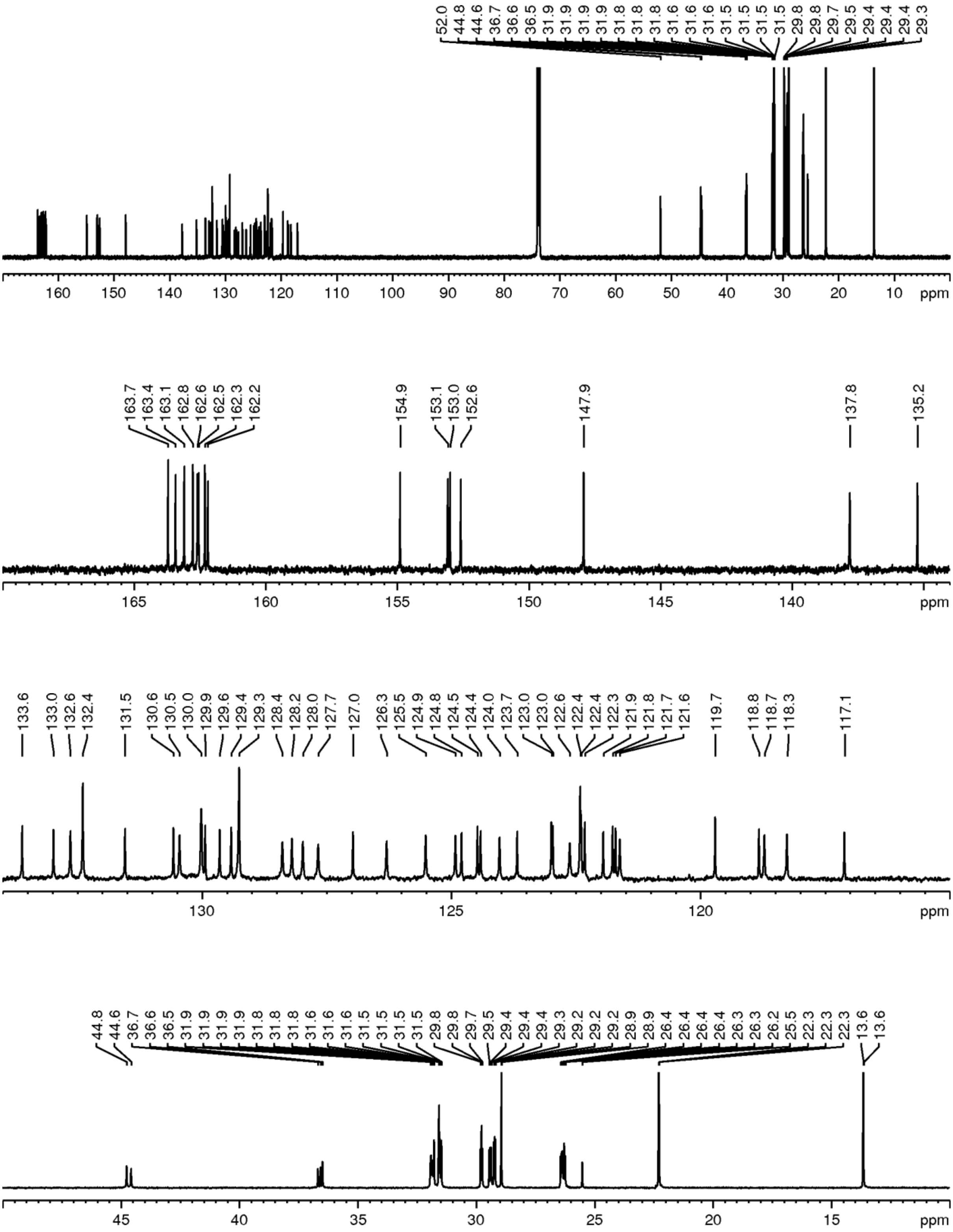

Supplementary Figure 50. NMR spectroscopic characterization. ^{13}C NMR spectrum (151 MHz, 384 K, TCE-d_2) of **D-PBI2-Br**.

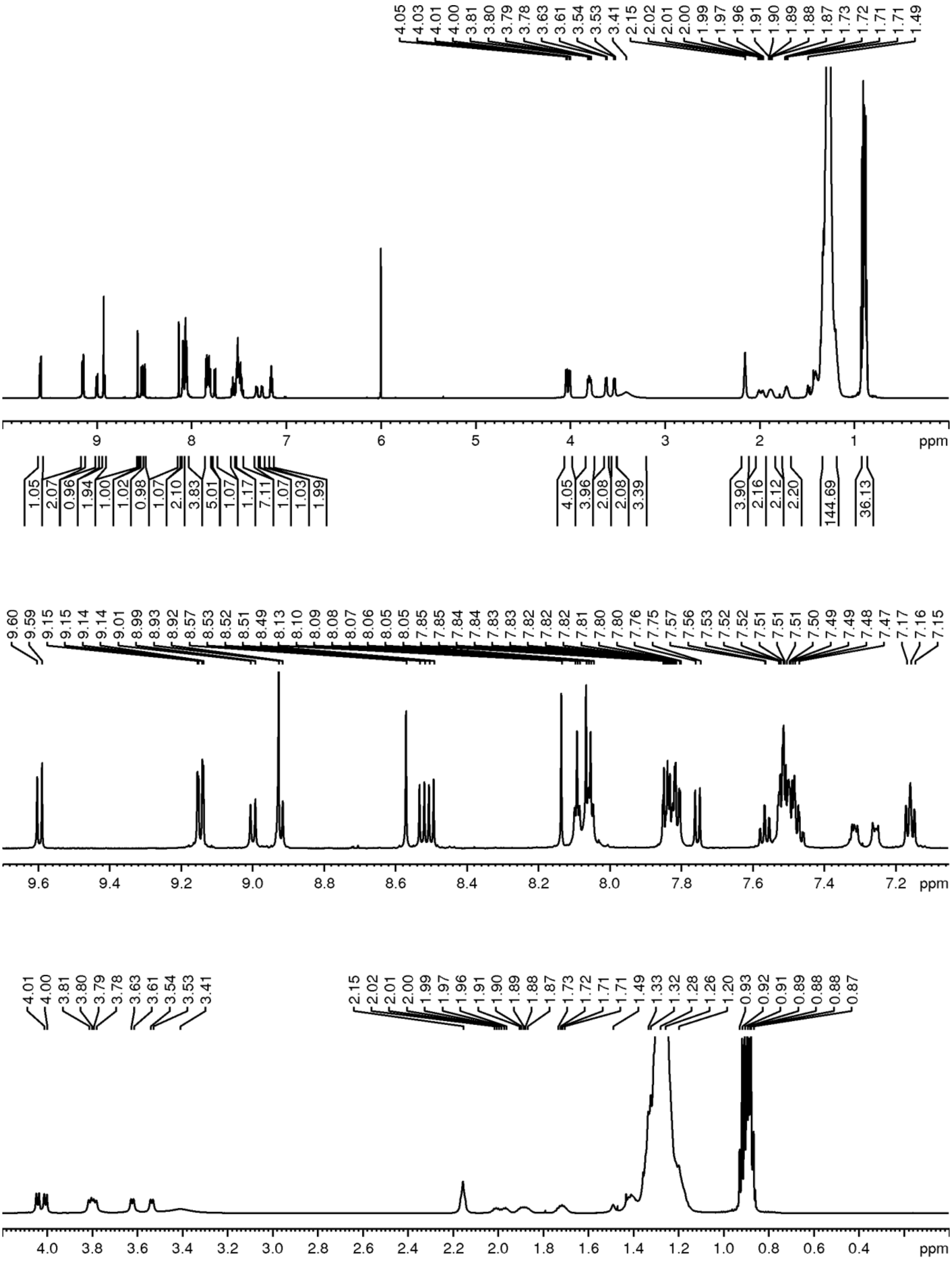

Supplementary Figure 51. NMR spectroscopic characterization. ^{1}H NMR spectrum (600 MHz, 384 K, TCE-d_2) of **D-PBI3-Br**.

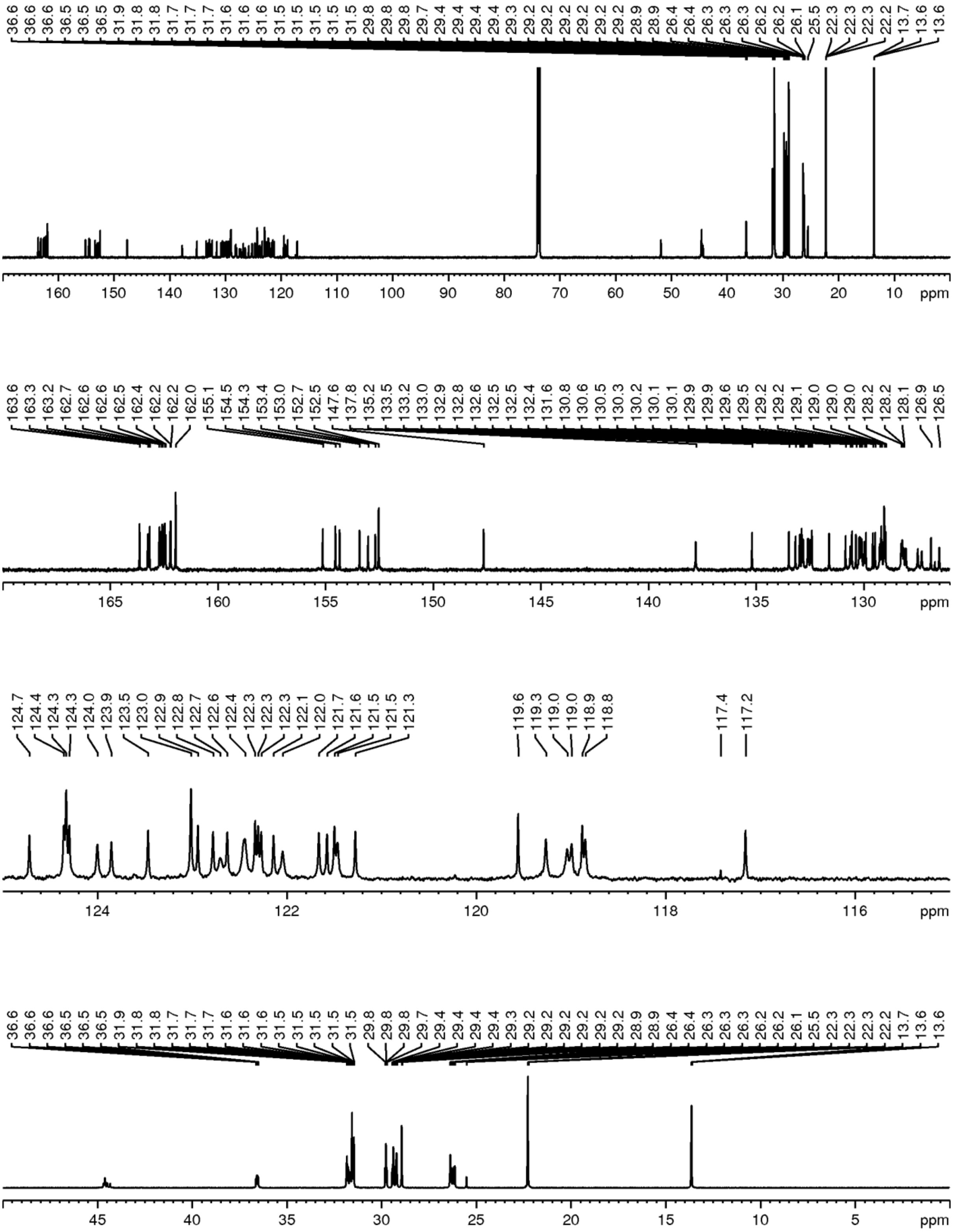

Supplementary Figure 52. NMR spectroscopic characterization. ^{13}C NMR spectrum (151 MHz, 384 K, TCE-d_2) of **D-PBI3-Br**.

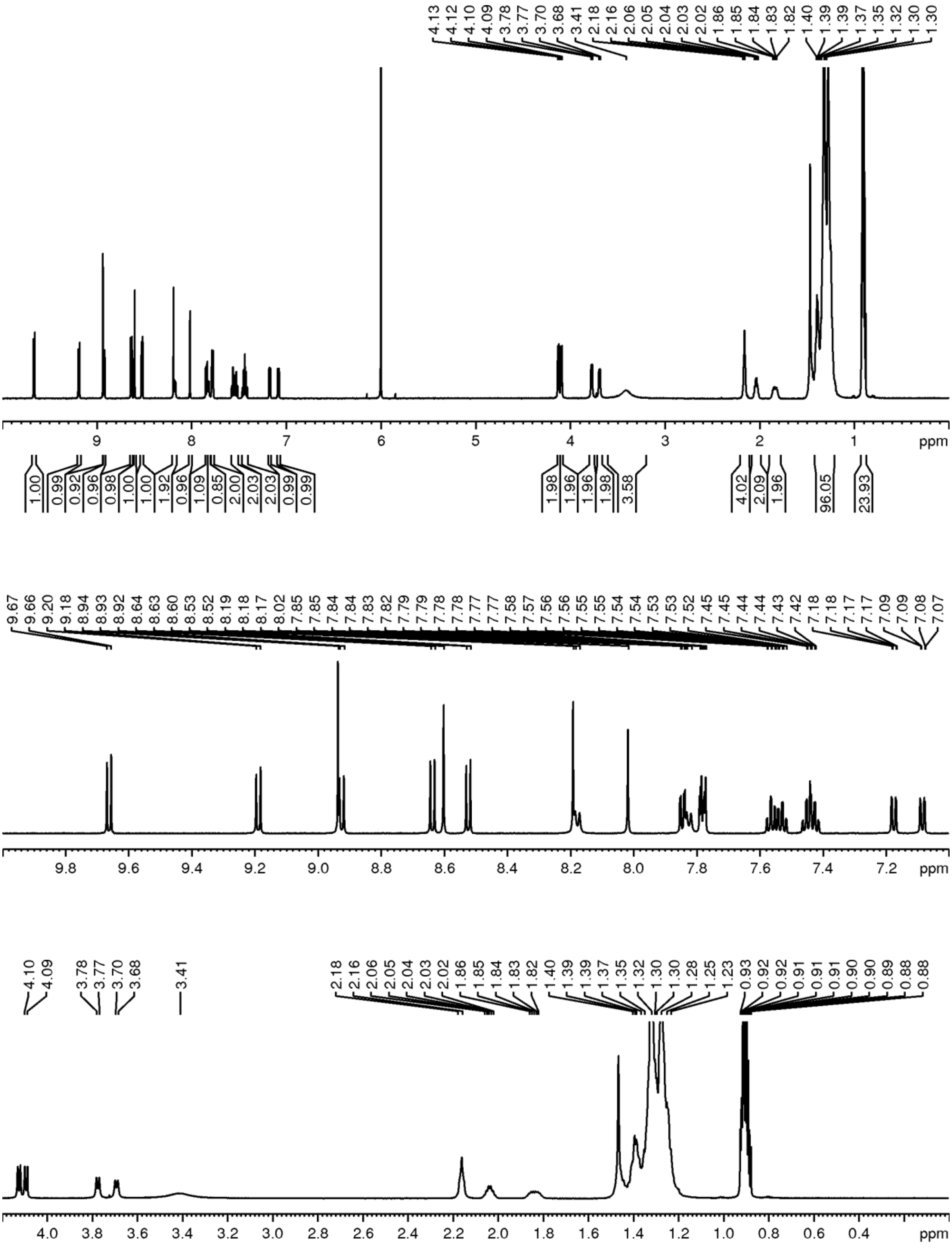

Supplementary Figure 53. NMR spectroscopic characterization. ^{1}H NMR spectrum (600 MHz, 384 K, TCE-d_2) of **DA-PBI2**.

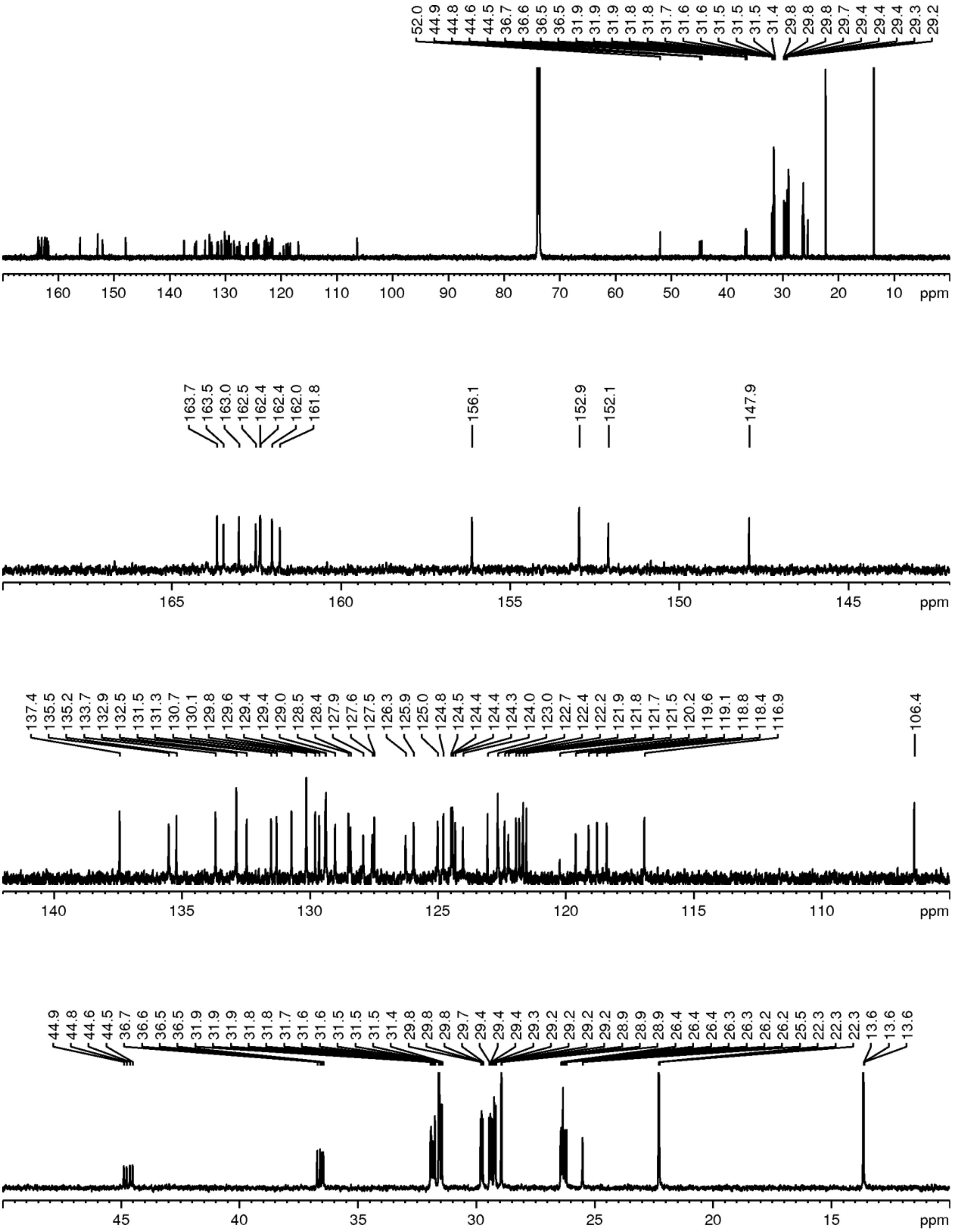

Supplementary Figure 54. NMR spectroscopic characterization. ^{13}C NMR spectrum (151 MHz, 384 K, TCE-d_2) of **DA-PBI2**.

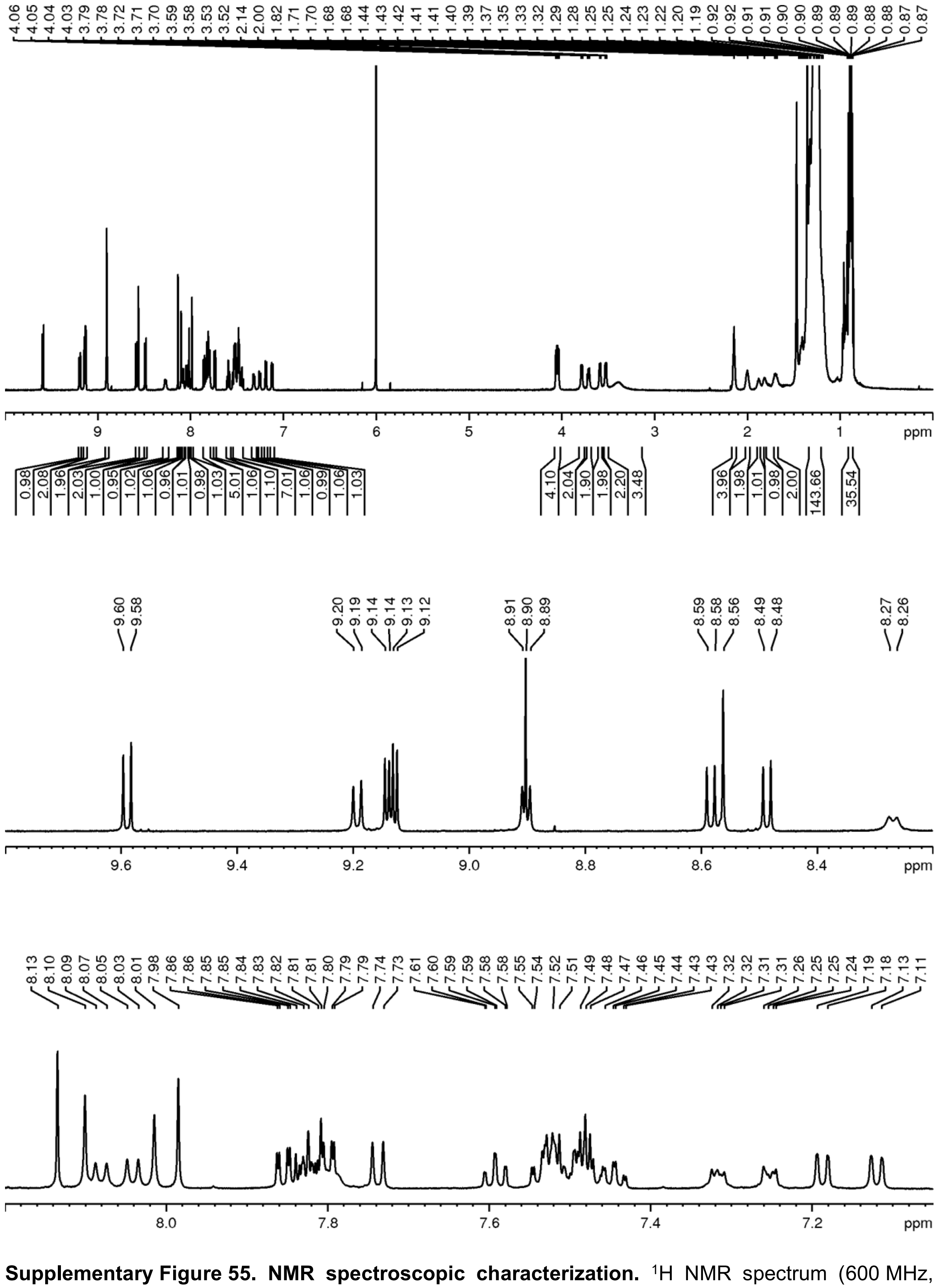

Supplementary Figure 55. NMR spectroscopic characterization. ^{1}H NMR spectrum (600 MHz, 384 K, TCE-d_2) of **DA-PBI3**.

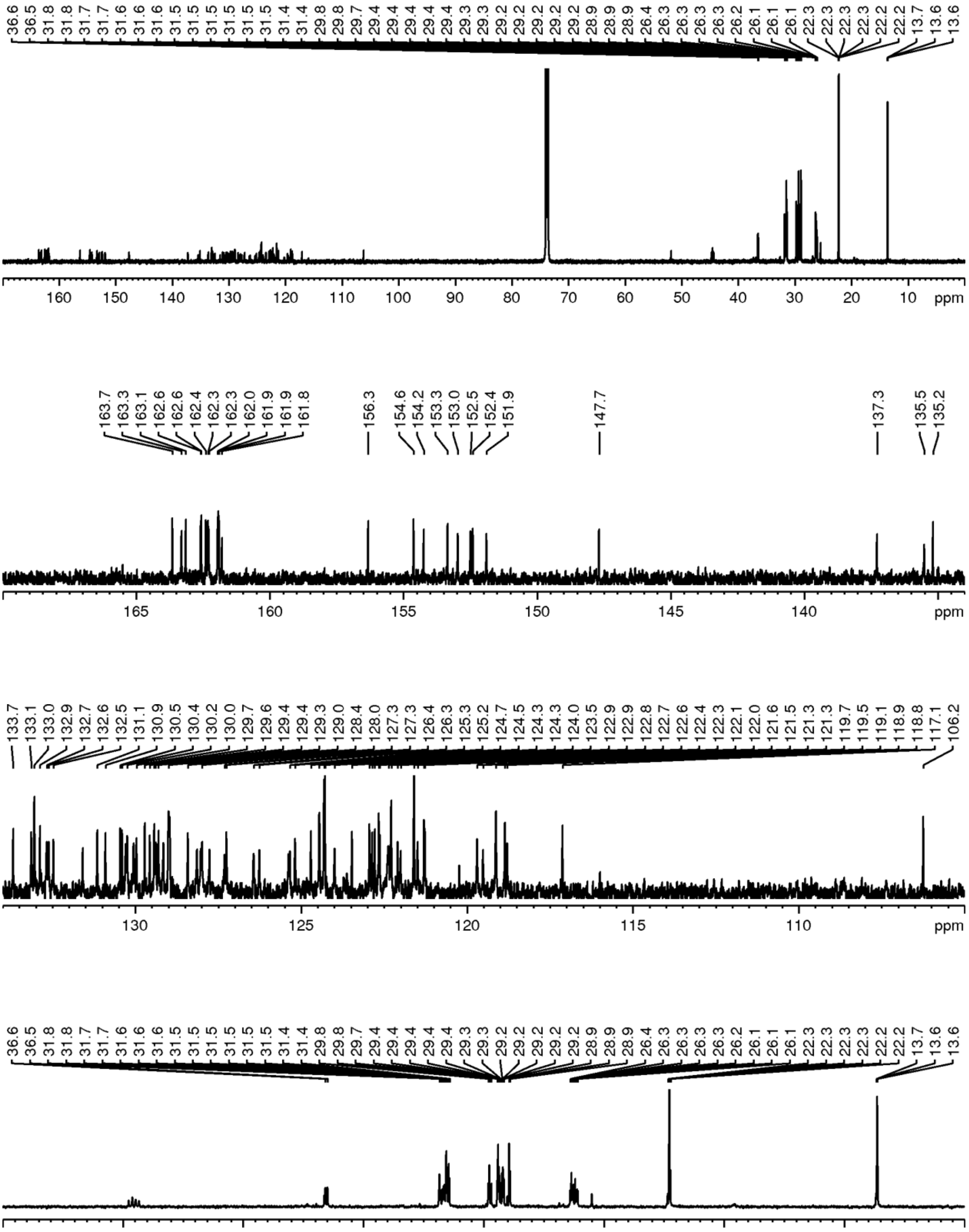

Supplementary Figure 56. NMR spectroscopic characterization. ^{13}C NMR spectrum (151 MHz, 384 K, TCE-d_2) of **DA-PBI3**.

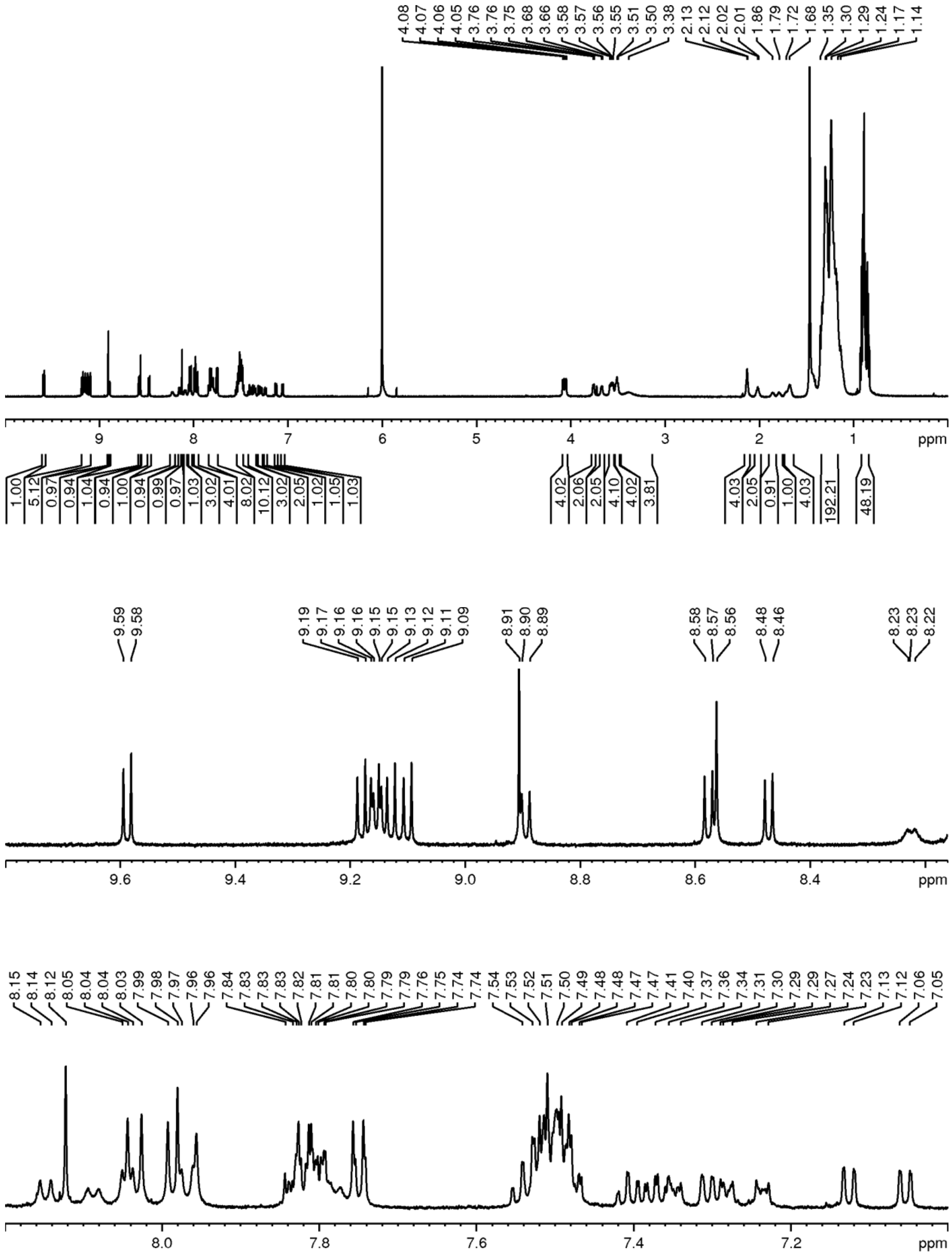

Supplementary Figure 57. NMR spectroscopic characterization. ^{1}H NMR spectrum (600 MHz, 384 K, TCE-d_2) of **DA-PBI4**.

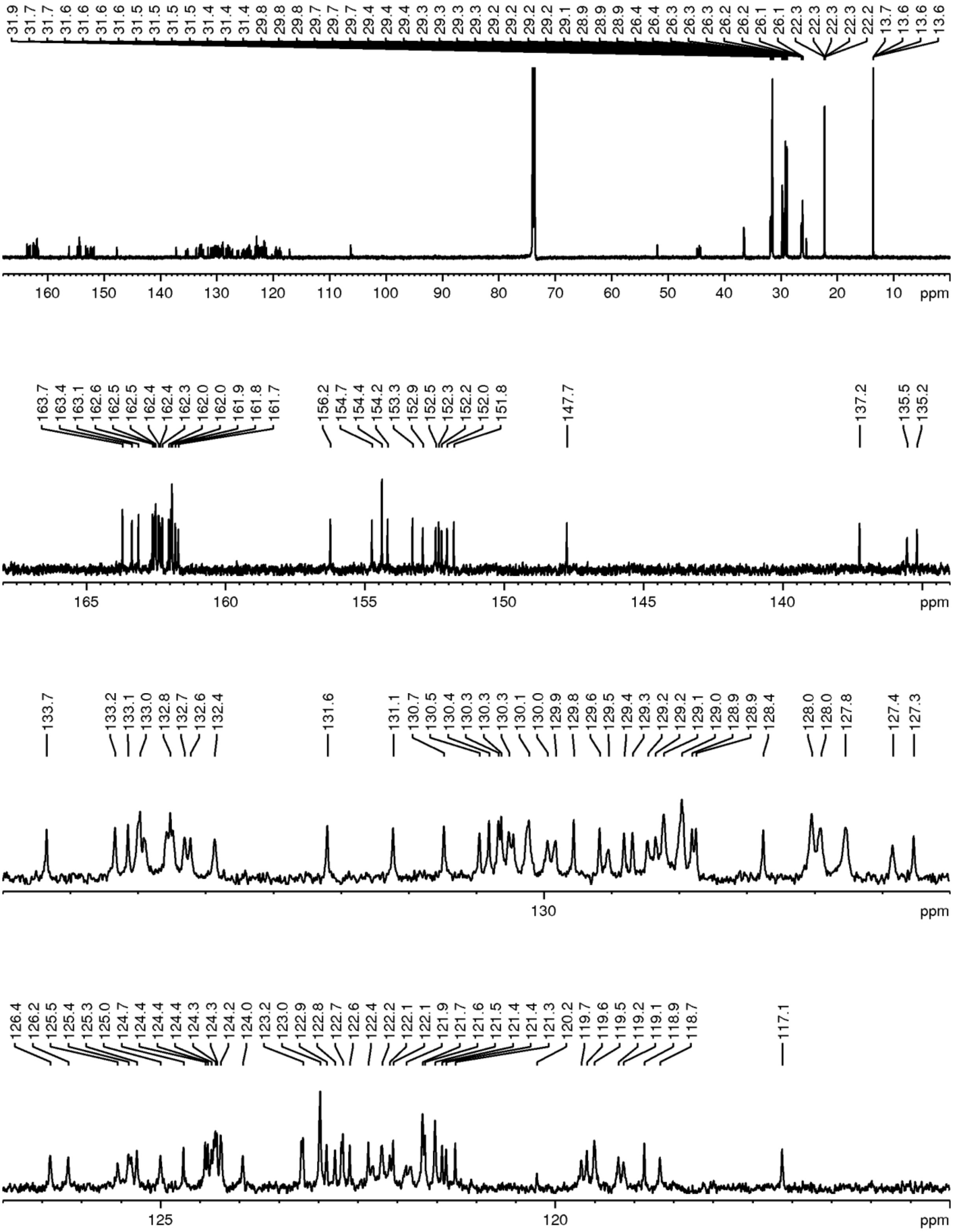

Supplementary Figure 58. NMR spectroscopic characterization. ^{13}C NMR spectrum (151 MHz, 384 K, TCE-d_2) of **DA-PBI4**.

10. Mass Spectrometry

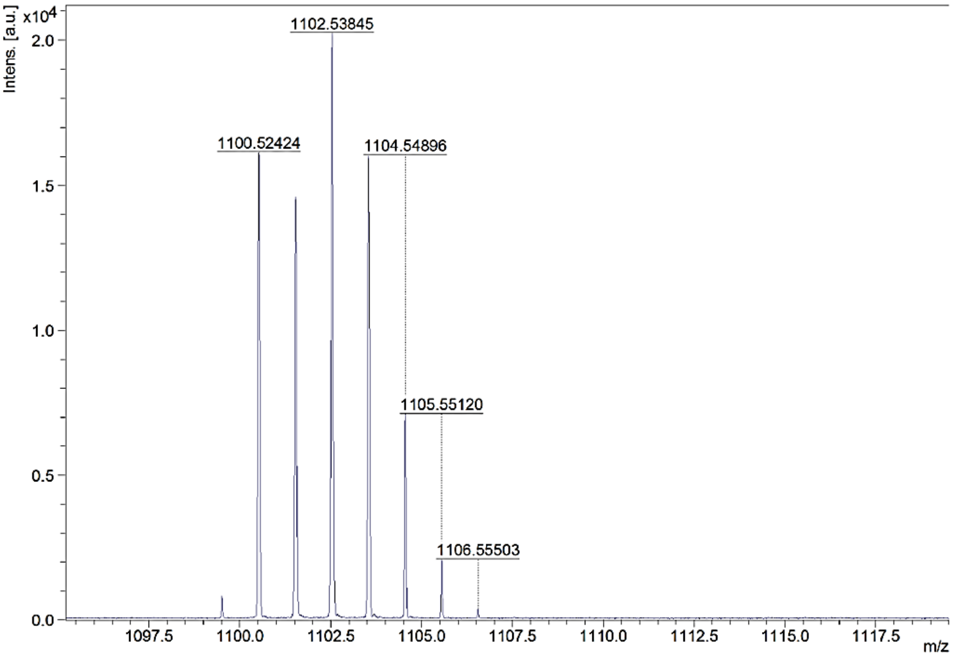

Supplementary Figure 59. Mass spectrometric characterization. HRMS (MALDI-TOF, positive mode, DCTB in chloroform) spectrum of PBI **3**.

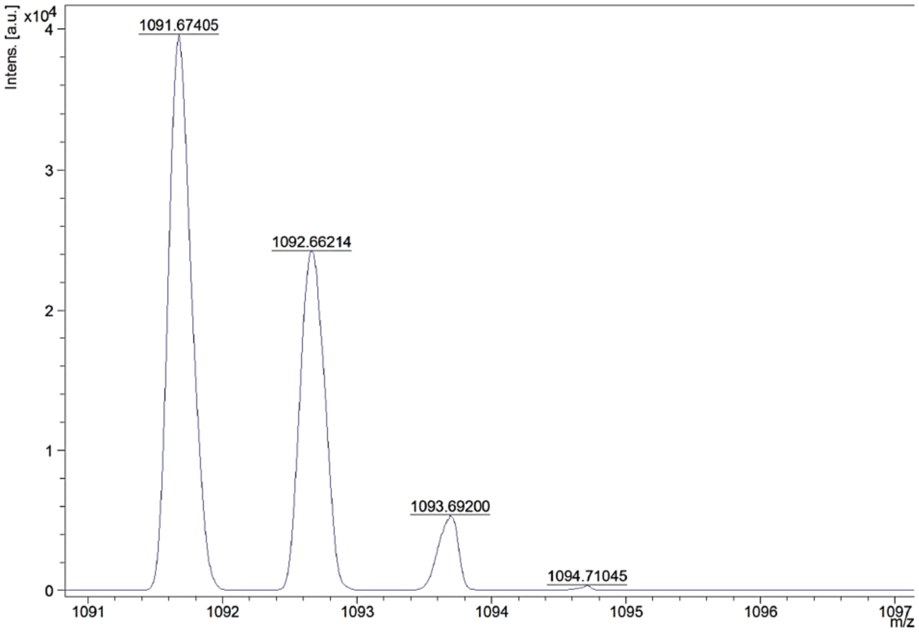

Supplementary Figure 60. Mass spectrometric characterization. HRMS (MALDI-TOF, negative mode, DCTB in chloroform) of PBI **4**.

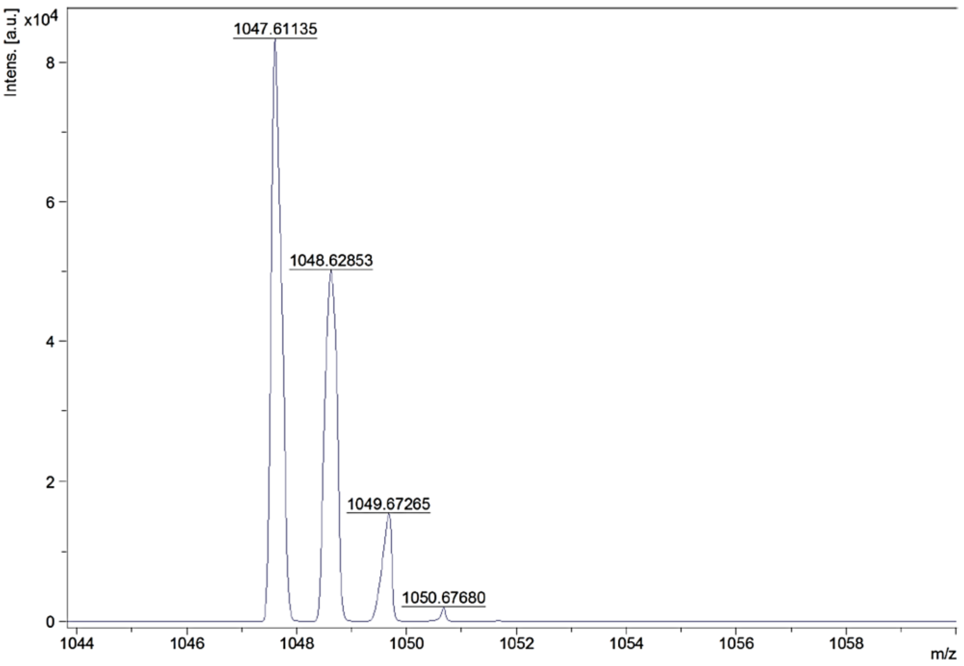

Supplementary Figure 61. Mass spectrometric characterization. HRMS (MALDI-TOF, negative mode, DCTB in chloroform) of PBI **5**.

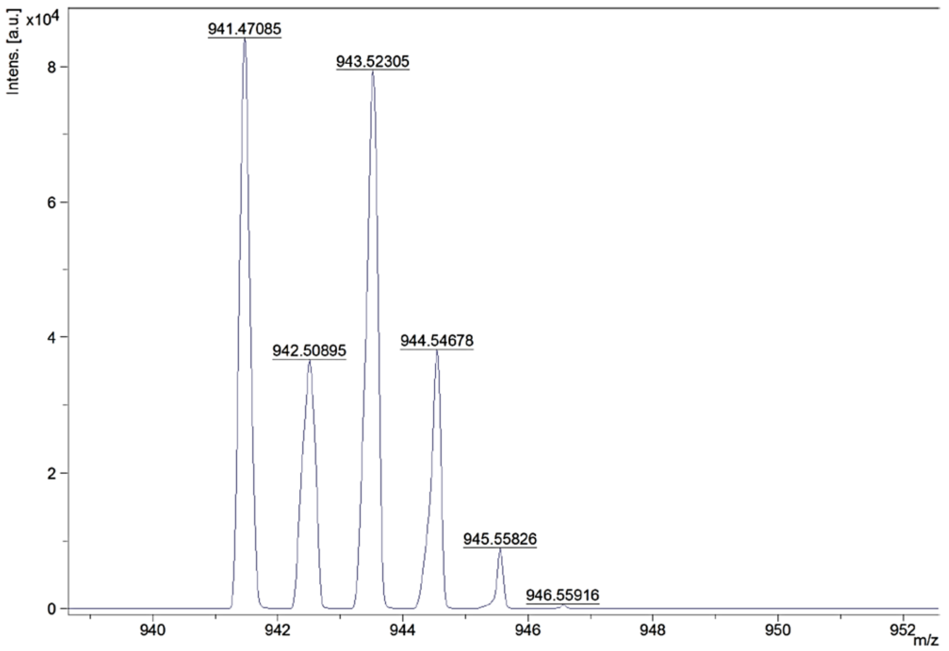

Supplementary Figure 62. Mass spectrometric characterization. HRMS (MALDI-TOF, negative mode, DCTB in chloroform) spectrum of PBI **8**.

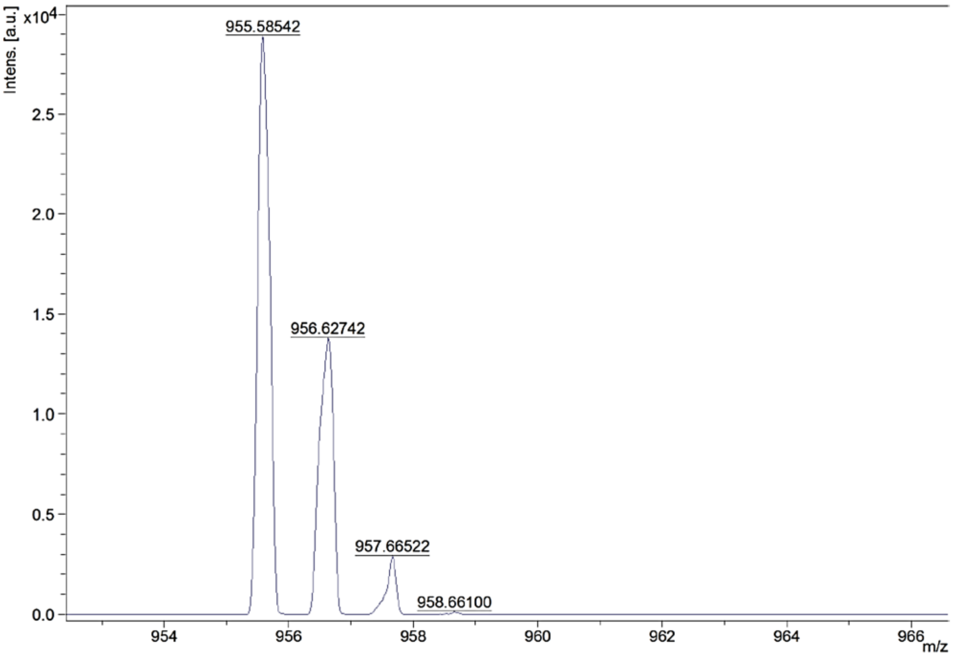

Supplementary Figure 63. Mass spectrometric characterization. HRMS (MALDI-TOF, negative mode, DCTB in chloroform) spectrum of reference molecule **A-PBI**.

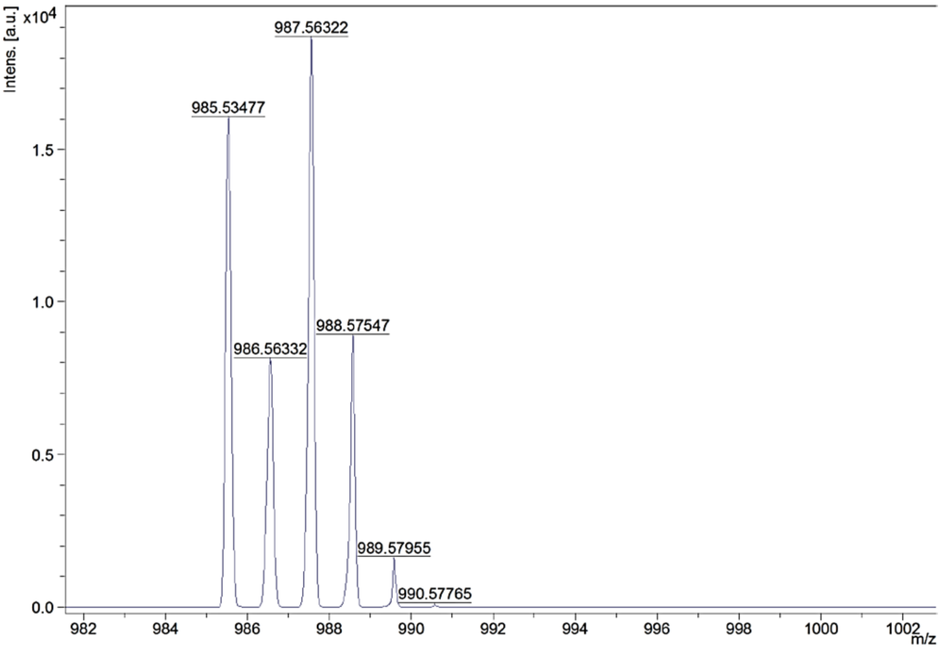

Supplementary Figure 64. Mass spectrometric characterization. HRMS (MALDI-TOF, negative mode, DCTB in chloroform) spectrum of PBI **7**.

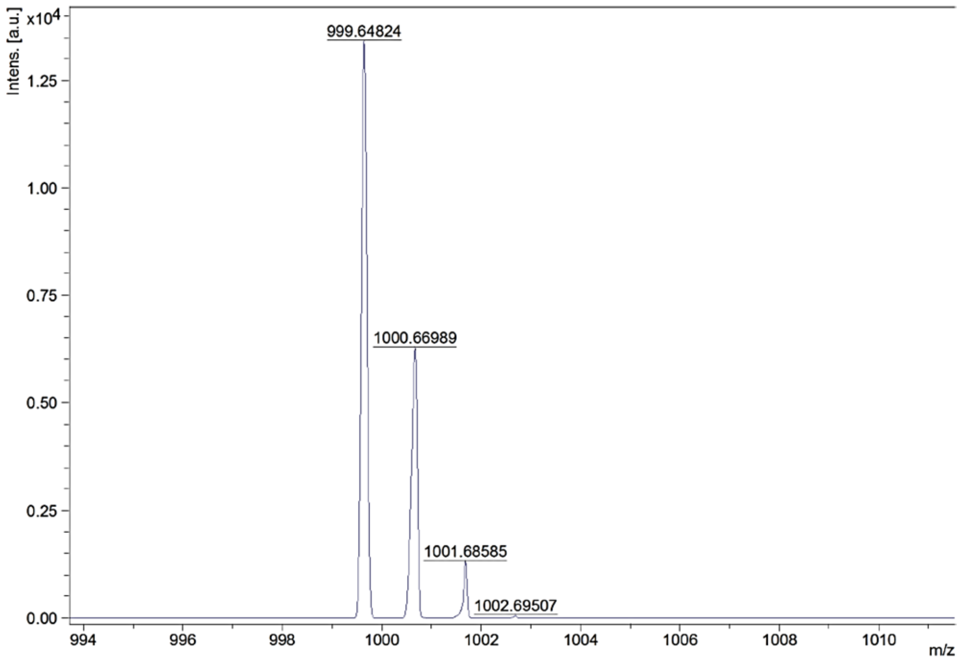

Supplementary Figure 65. Mass spectrometric characterization. HRMS (MALDI-TOF, negative mode, DCTB in chloroform) spectrum of reference molecule **D-PBI**.

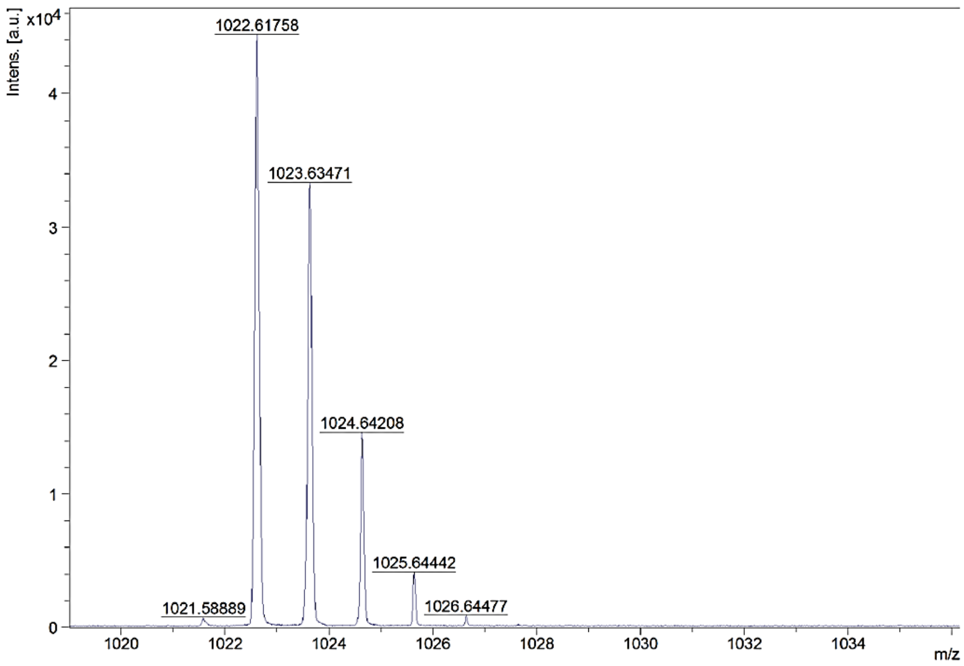

Supplementary Figure 66. Mass spectrometric characterization. HRMS (MALDI-TOF, positive mode, DCTB in chloroform) spectrum of reference molecule **PBI-1**.

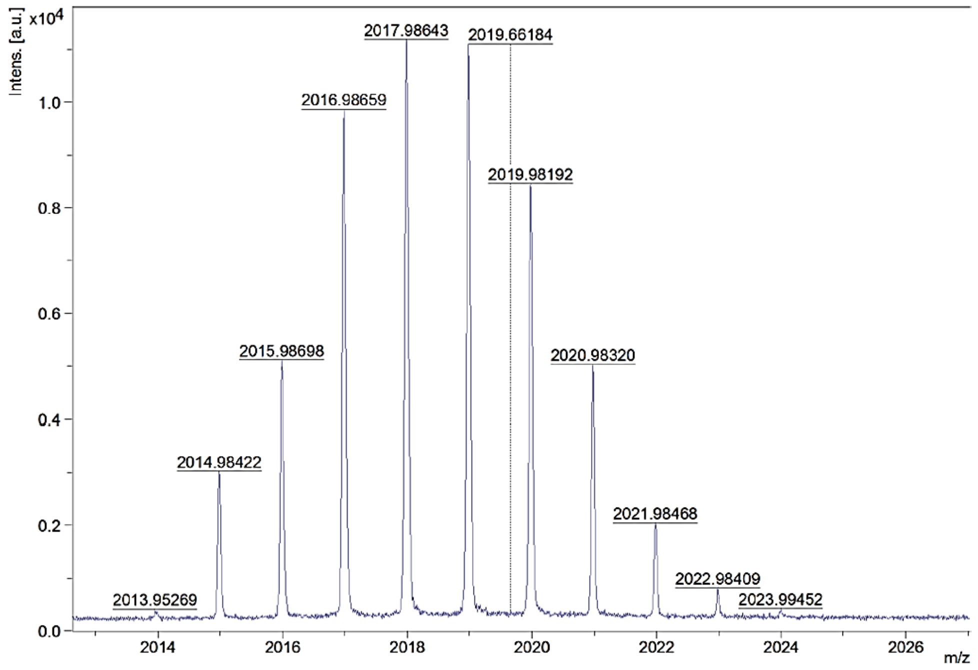

Supplementary Figure 67. Mass spectrometric characterization. HRMS (MALDI-TOF, positive mode, DCTB in chloroform) spectrum of **Br-PBI2-Br**.

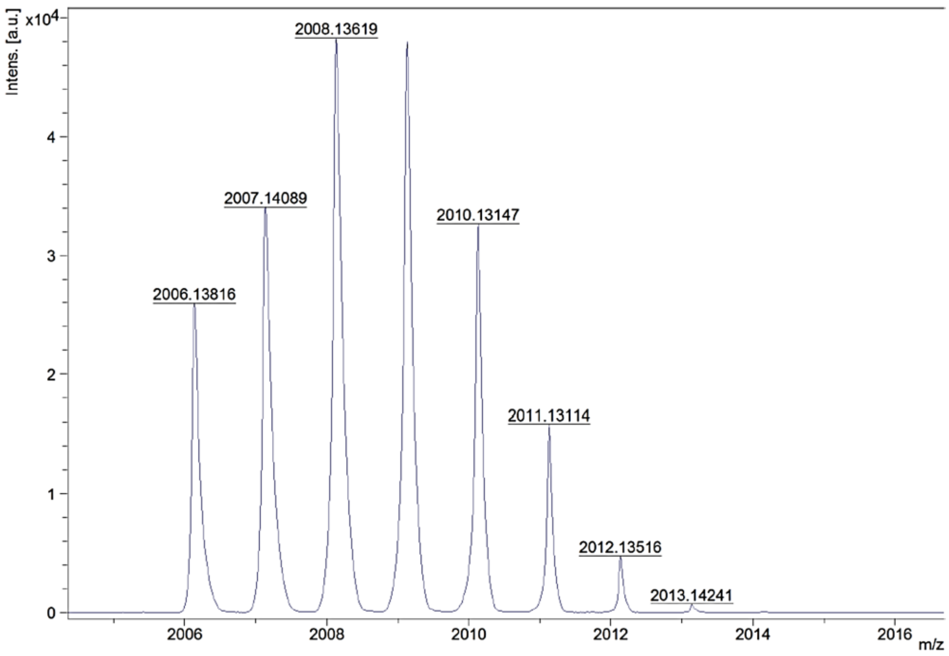

Supplementary Figure 68. Mass spectrometric characterization. HRMS (MALDI-TOF, negative mode, DCTB in chloroform) spectrum of **D-PBI2-Br.**

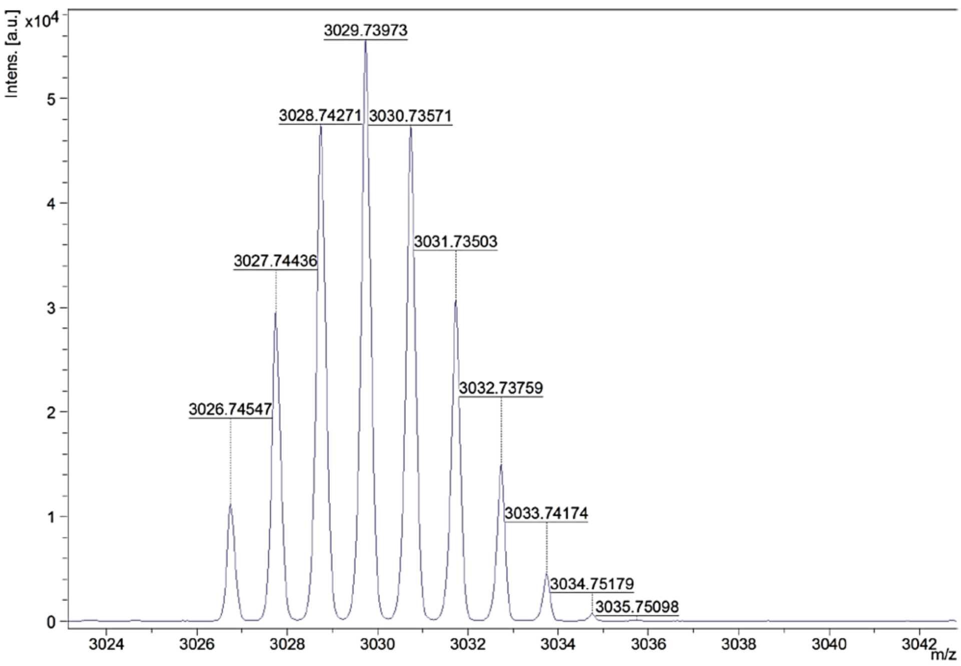

Supplementary Figure 69. Mass spectrometric characterization. HRMS (MALDI-TOF, negative mode, DCTB in chloroform) spectrum of **D-PBI3-Br.**

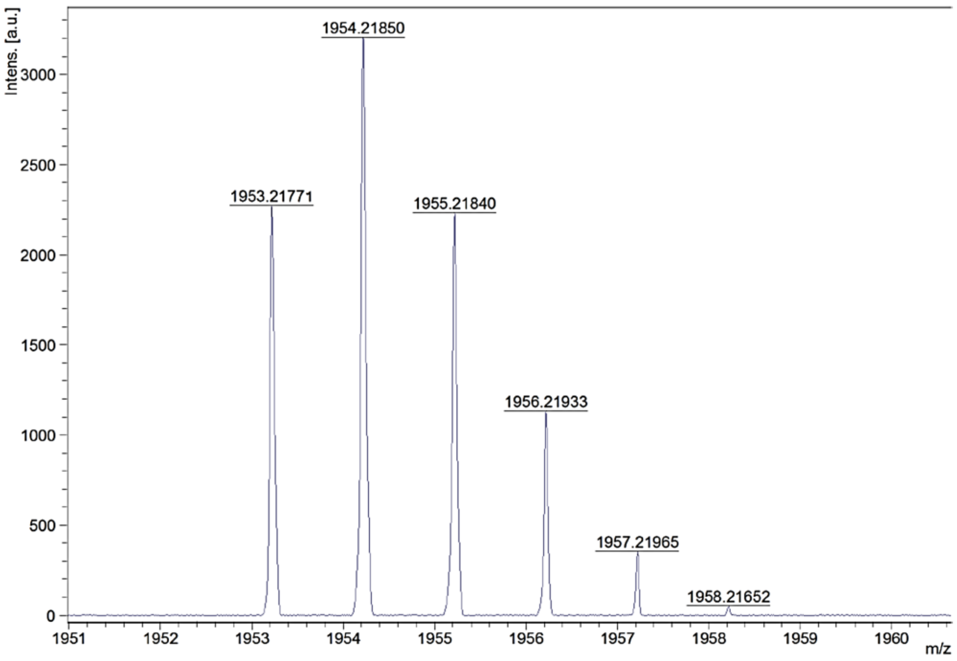

Supplementary Figure 70. Mass spectrometric characterization. HRMS (MALDI-TOF, negative mode, DCTB in chloroform) spectrum of **DA-PBI2**.

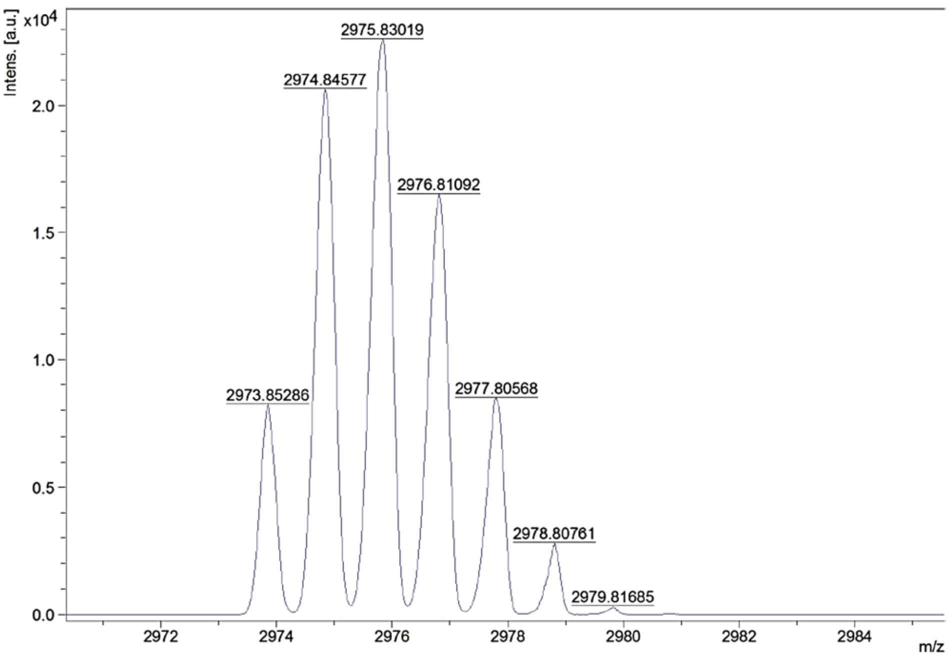

Supplementary Figure 71. Mass spectrometric characterization. HRMS (MALDI-TOF, negative mode, DCTB in chloroform) spectrum of **DA-PBI3**.

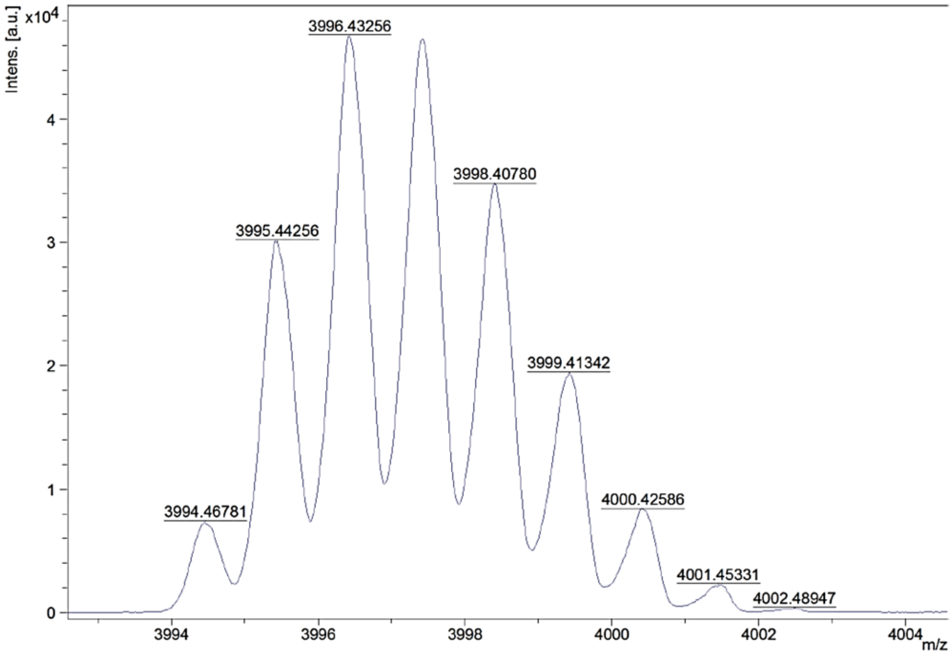

Supplementary Figure 72. Mass spectrometric characterization. HRMS (MALDI-TOF, negative mode, DCTB in chloroform) spectrum of **DA-PBI4**.

11. References

1. Guo, X. *et al.* Thieno[3,4- c]pyrrole-4,6-dione-based polymer semiconductors: Toward high-performance, air-stable organic thin-film transistors. *J. Am. Chem. Soc.* **133**, 13685–13697 (2011).

2. Hong, Y. *et al.* Steering the multiexciton generation in slip-stacked perylene dye array via exciton coupling. *Nat. Commun.* **13**, 4488 (2022).

3. Zhang, S. *et al.* Perylene diimide copolymers with dithienothiophene and dithienopyrrole: Use in n-channel and ambipolar field-effect transistors. *J. Polym. Sci. Part A Polym. Chem.* **51**, 1550–1558 (2013).

4. Prasad, T. E. V., Phanibhushan, A. & Prasad, D. H. L. Densities and Viscosities of Binary Mixtures of m-Cresol with Some Chlorohydrocarbons. *J. Solution Chem.* **34**, 1263–1272 (2005).

5. Baragi, J. G., Aralaguppi, M. I., Aminabhavi, T. M., Kariduraganavar, M. Y. & Kittur, A. S. Density, Viscosity, Refractive Index, and Speed of Sound for Binary Mixtures of Anisole with 2-Chloroethanol, 1,4-Dioxane, Tetrachloroethylene, Tetrachloroethane, DMF, DMSO, and Diethyl Oxalate at (298.15, 303.15, and 308.15) K. *J. Chem. Eng. Data* **50**, 910–916 (2005).

6. Snellenburg, J. J., Laptenok, S. P., Seger, R., Mullen, K. M. & Stokkum, I. H. M. van. Glotaran : A Java -Based Graphical User Interface for the R Package TIMP. *J. Stat. Softw.* **49**, 1–22 (2012).

7. van Stokkum, I. H. M., Larsen, D. S. & van Grondelle, R. Global and target analysis of time-resolved spectra. *Biochim. Biophys. Acta - Bioenerg.* **1657**, 82–104 (2004).

8. Kaufmann, C., Bialas, D., Stolte, M. & Würthner, F. Discrete π-Stacks of Perylene Bisimide Dyes within Folda-Dimers: Insight into Long- and Short-Range Exciton Coupling. *J. Am. Chem. Soc.* **140**, 9986–9995 (2018).

9. Edward, J. T. Molecular volumes and the Stokes-Einstein equation. *J. Chem. Educ.* **47**, 261 (1970).

10. Frisch, M. *et al*. Inc., Wallingford CT,. *Gaussian 16, Revis. C.01* (2016).

11. Chai, J. Da & Head-Gordon, M. Long-range corrected hybrid density functionals with damped atom–atom dispersion corrections. *Phys. Chem. Chem. Phys.* **10**, 6615–6620

(2008).

12. Weigend, F. & Ahlrichs, R. Balanced basis sets of split valence, triple zeta valence and quadruple zeta valence quality for H to Rn: Design and assessment of accuracy. *Phys. Chem. Chem. Phys.* **7**, 3297–3305 (2005).

13. Lu, T. & Chen, F. Multiwfn: A multifunctional wavefunction analyzer. *J. Comput. Chem.* **33**, 580–592 (2012).

14. Chako, N. Q. Absorption of Light in Organic Compounds. *J. Chem. Phys.* **2**, 644–653 (1934).

15. Chang, J. C. Monopole effects on electronic excitation interactions between large molecules. I. Application to energy transfer in chlorophylls. *J. Chem. Phys.* **67**, 3901–3909 (1977).

16. Kenny, E. P. & Kassal, I. Benchmarking Calculations of Excitonic Couplings between Bacteriochlorophylls. *J. Phys. Chem. B* **120**, 25–32 (2016).

17. te Velde, G. *et al.* Chemistry with ADF. *J. Comput. Chem.* **22**, 931–967 (2001).

18. Fonseca Guerra, C., Snijders, J. G., Te Velde, G. & Baerends, E. J. Towards an order-N DFT method. *Theor. Chem. Acc.* **99**, 391–403 (1998).

19. Perdew, J. P. & Burke, K. Generalized gradient approximation for the exchange-correlation hole of a many-electron system. *Phys. Rev. B - Condens. Matter Mater. Phys.* **54**, 16533–16539 (1996).

20. Barbieri, P. L., Fantin, P. A. & Jorge, F. E. Gaussian basis sets of triple and quadruple zeta valence quality for correlated wave functions. *Mol. Phys.* **104**, 2945–2954 (2006).

21. Valeev, E. F., Coropceanu, V., Da Silva Filho, D. A., Salman, S. & Brédas, J. L. Effect of electronic polarization on charge-transport parameters in molecular organic semiconductors. *J. Am. Chem. Soc.* **128**, 9882–9886 (2006).

22. Hestand, N. J. & Spano, F. C. Molecular Aggregate Photophysics beyond the Kasha Model: Novel Design Principles for Organic Materials. *Acc. Chem. Res.* **50**, 341–350 (2017).

23. Scholes, G. D. & Ghiggino, K. P. Electronic Interactions and Interchromophore Excitation Transfer. *J. Phys. Chem. A* **98**, 4580–4590 (1994).

24. Lin, C., Kim, T., Schultz, J. D., Young, R. M. & Wasielewski, M. R. Accelerating symmetry-breaking charge separation in a perylenediimide trimer through a vibronically coherent dimer intermediate. *Nat. Chem. 2022 147* **14**, 786–793 (2022).

25. Martin, R. L. Natural transition orbitals. *J. Chem. Phys.* **118**, 4775–4777 (2003).

26. Hou, Y. *et al.* Charge separation, charge recombination, long-lived charge transfer state formation and intersystem crossing in organic electron donor/acceptor dyads. *J. Mater. Chem. C* **7**, 12048–12074 (2019).

27. Spenst, P., Young, R. M., Wasielewski, M. R. & Würthner, F. Guest and solvent modulated photo-driven charge separation and triplet generation in a perylene bisimide cyclophane. *Chem. Sci.* **7**, 5428–5434 (2016).

28. Hill, J. P. *et al.* A Novel Bis(zinc–porphyrin)–Oxoporphyrinogen Donor–Acceptor Triad: Synthesis, Electrochemical, Computational and Photochemical Studies. *European J. Org. Chem.* **2006**, 595–603 (2006).

29. Fang, S. *et al.* Interchromophore Rotation-Related Ultrafast Charge Separation at Excited States in Head-to-Tail Linked Perylene Diimide Dyads. *J. Phys. Chem. C* **123**, 23306–23311 (2019).

30. Weller, A. Photoinduced Electron Transfer in Solution: Exciplex and Radical Ion Pair Formation Free Enthalpies and their Solvent Dependence. *Zeitschrift für Phys. Chemie* **133**, 93–98 (1982).

31. Renner, R. *et al.* Substituent-dependent absorption and fluorescence properties of perylene bisimide radical anions and dianions. *Mater. Horizons* **9**, 350–359 (2022).
